# Parametric Life Cycle Assessment of Chemical Recycling of Nylon‑6 to Caprolactam

**DOI:** 10.1021/acs.est.5c16101

**Published:** 2026-02-16

**Authors:** Ann-Joelle Minor, Ruben Goldhahn, Caroline Ganzer, Michaël Lejeune, Liisa Rihko-Struckmann, Kai Sundmacher

**Affiliations:** † Chair for Process Systems Engineering, Otto von Guericke University Magdeburg, Universitätsplatz 2, Magdeburg 39106, Germany; ‡ Department Process Systems Engineering, 28307 Max Planck Institute for Dynamics of Complex Technical Systems, Sandtorstrasse 1, Magdeburg 39106, Germany; § Sustainability in Manufacturing and Life Cycle Engineering Research Group, School of Mechanical and Manufacturing Engineering, 7800 The University of New South Wales, Sydney 2052, Australia; ∥ Training Centre for the Global Hydrogen Economy, Australian Research Council, Sydney 2052, Australia

**Keywords:** LCA, process analysis, waste treatment, carbon budget, hybrid recycling approach

## Abstract

Chemical recycling is gaining attention to advance the circular economy. This study presents the first life cycle assessment (LCA) of real-world waste polyamide 6 (PA6) remonomerization to caprolactam, evaluating four depolymerization routes: acidic, hydrothermal, alcoholysis, and alkaline. We established an automated Python-Aspen Plus-LCA workflow, systematically mapping variations in PA6 waste composition and key process parameters onto probability distributions of environmental impacts. Results show that the hydrothermal process has the highest impacts in six of nine categories, while the solvent-free, alkaline NaOH route consistently shows the lowest. Despite lower energy demands, the acidic H_3_PO_4_ process is not environmentally superior to the alcoholysis route. For the hydrothermal route, results are strongly driven by the water-to-feed ratio. However, its global warming potential (GWP) remains above that of fossil-based caprolactam. In contrast, the alcoholysis and acidic processes lower GWP by ∼35%, whereas the NaOH route achieves an ∼80% reduction to 1.46 kg CO_2_-eq/kg caprolactam. Although chemical recycling can mitigate impacts, no process consistently meets the net-zero emission carbon budget needed to limit global warming to 1.5 °C. As the NaOH process comes closest to this target and demonstrates the strongest environmental and economic performance, future research should focus on scaling-up solvent-free chemical recycling.

## Graphical abstract

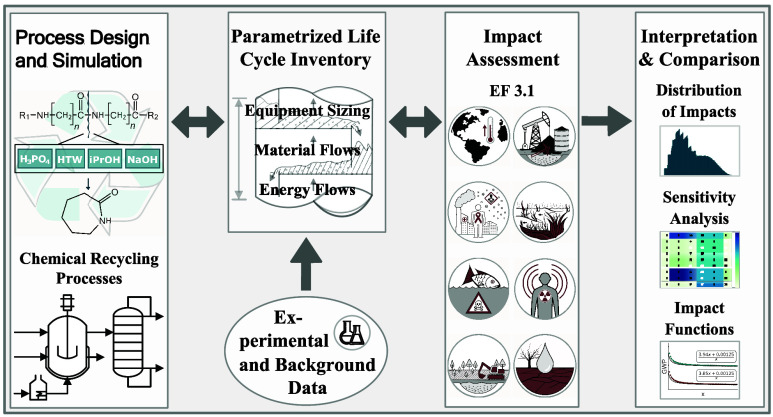

## 1 Introduction

Nylon, e.g., polyamide 6 (PA6), is among the highest-impact synthetic textile globally, with production projected to reach 10.4 million tonnes by 2027.^1,2^ This surge poses significant challenges, including environmental pollution and increased reliance on petroleum-derived resources, which are central to nylon production.^3^ As net-zero emissions targets increase pressure to reduce emissions,^200^ chemical recycling is increasingly investigated.^4,5^ This method overcomes the key limitation of mechanical recycling: property deterioration over multiple cycles.^6–9^ Chemical recycling of PA6, particularly through the “back-to-monomer” or remonomerization process, involves depolymerizing the polymer back to its monomer, ε-caprolactam (CL),^10^ using solvents and/or catalysts under relatively mild conditions.^11,12^ Different chemical remonomerization pathways enable repeated polymerization-depolymerization cycles, aligning with circular economy principles.^4,13,14^

Building on these documented pathways, our recent study^4^ analyzed four main depolymerization processes of PA6: acidic hydrolysis using H_3_PO_4_ combined with steam stripping, hydrothermal depolymerization under near-critical conditions using high-temperature water (HTW), solvent-free, alkaline depolymerization with NaOH, and alcoholysis via iPrOH. For those, we conducted a techno-economic assessment (TEA) and found that chemical recycling of PA6 can be economically viable, with the alkaline NaOH process emerging as the most cost-effective.^4^ Despite past and ongoing efforts in chemical recycling, only a minor fraction of PA6 is chemically recycled.^15,16^ To improve this, research should quantify both environmental and economic benefits to support industrial adoption through comparative assessments.^16^

Life cycle assessment (LCA) has become an essential decision-making tool for evaluating the environmental impacts of polymer end-of-life options,^17,18^ with significant methodological advancements reflected in a 160% increase in plastic recycling studies from 2010 to 2019.^19–21^ Numerous studies have assessed mechanical and chemical recycling as well as incineration with energy recovery,^22–24^ showing that all options involve environmental trade-offs and that no single technology outperforms across all impact categories.^25^

While these comparisons provide valuable insights, chemical recycling is widely recognized as a complement to, rather than a competitor with, mechanical recycling.^19^ Consequently, Davidson et al.^19^ recommend that LCA should focus on assessing various chemical recycling techniques, both individually and comparatively, instead of contrasting them with mechanical recycling. Furthermore, most LCA studies on plastic recycling focus on waste treatment rather than on product production,^25–27^ even though significant climate benefits are achieved mainly when plastics are chemically recycled to value-added products.^28^ Accordingly, the literature lacks environmental comparisons of different chemical recycling techniques and assessments from a product rather than a waste perspective.

To our knowledge, no study has yet provided an environmental assessment of distinct PA6 chemical recycling pathways, benchmarked against each other and against conventional fossil-based CL production, or quantified their absolute climate performance relative to a 1.5 °C-compatible carbon budget. This gap is compounded by the lack of LCA data sets for CL production itself and the scarcity of studies that consider varying, real-world waste compositions and prepurification scenarios.

Here, we address these deficiencies by conducting the first comparative LCA of four PA6 depolymerization routes, acidic, hydrothermal, alkaline, and alcoholysis, explicitly incorporating real-world PA6 waste compositions and prepurification options. Our hybrid approach, in line with the waste hierarchy, integrates physical (dissolution-based) and chemical recycling.^12^ Given their technology readiness level (TRL) 3–5 maturity, we conduct a parametric, comparative, attributional LCA that assesses the environmental impacts of producing 1 kg of recycled CL, compares routes, identifies key process bottlenecks and uncertainties, and quantifies how different feedstock scenarios and prepurification data sets influence impact results. By integrating detailed process modeling with automated, scenario-based Monte Carlo simulations, we not only report mean impacts but also determine uncertainty ranges, probability distributions, process sensitivities, and surrogate impact functions for each route. In addition, we relate our environmental results to our previous TEA,^4^ bridging the gap between financial and environmental trade-offs. Ultimately, we benchmark the recycling pathways against fossil-based CL, PA6 incineration and an absolute, 1.5 °C-consistent carbon budget allocated to the plastics sector.

## 2 Methods

### 2.1 Workflow

For this study, we automated LCA calculations by integrating Aspen Plus v12.0 with Python v3.9.18,^29,30^ establishing a fully connected workflow for simulation-driven environmental assessment. This integrated approach enables a direct, dynamic connection between Python and Aspen Plus, allowing automated extraction and control of key process variables, such as mass and energy flows, temperatures, and heat duties, and supporting the rapid assessment of environmental impacts.^31,32^ Using the extracted inventories from the process simulation, we conducted LCA simulations with the open-source lca-algebraic Python library, which builds on the Brightway2 ecosystem.^33–35^ This enables fast symbolic LCA computations, facilitating advanced Monte Carlo simulations and statistical analyses.^36,37^ An exemplary case study explaining and demonstrating this workflow is provided online.^38^

### 2.2 Process Design and Simulation of the Chemical Recycling Processes

All processes were modeled under harmonized and equivalent conditions. Process parameters, including PA6 input flow rates, reactor volumes, and reaction agent ratios, were set based on literature-reported values for each pathway to maintain consistency across routes. Component databases, equilibrium data retrieval, and kinetic assumptions align with our prior work.^4^ Further details on thermodynamic modeling, kinetic modeling, heat integration, utility selection, and further assumptions can be found in Supporting Information Sections A.3 and C.3.

### 2.3 Life Cycle Assessment

This study follows the ISO 14040 and 14044 standards for life cycle assessment (LCA), covering all four phases: goal and scope definition, inventory analysis, impact assessment, and interpretation.^39,40^

#### 2.3.1 Goal and Scope Definition

The methodological choices regarding the goal and scope definition are justified in detail in Supporting Information Section E1. The goal of this LCA is to

- Compare the environmental impacts of producing CL from PA6 via four remonomerization pathways;
- Identify key process bottlenecks and uncertainties, including the influence of variable waste composition, sorting, and prepurification scenarios on overall results;
- Benchmark each recycling pathway against fossil-based CL production, PA6 incineration, and a 1.5 °C-compatible carbon budget;
- Support decision-making, provided differences in impacts and uncertainties are sufficiently pronounced.

Given that this study quantifies the lifecycle impacts of CL production, rather than system-wide changes from substituting virgin fossil-based CL, we used an attributional LCA approach.^41,42^ A consequential LCA would require extensive market and industrial production data for CL, which is currently unavailable in existing LCA databases. The functional unit is defined as 1 kg of recycled CL produced in Germany with a purity of at least 99.9 wt %. The scope covers the process from PA6 waste prior to collection and sorting (gate) to the production of CL (factory gate), with Germany as the geographical scope.

##### 2.3.1.1 Allocation Approach

Attributional LCA uses allocation by partitioning to address multifunctional processes, such as those that generate by-products or treat waste while simultaneously generating valuable product streams.^42,43^ Hence, allocation rules must determine how much of the impacts from the initial life cycle, which generates the waste to be recycled, are attributed to the recycling process. While some studies argue that recycled materials should not inherit the impacts of their initial (often fossil-based) production, others emphasize that recycling is only possible because of that first life cycle. Due to the lack of reliable industrial pricing data for PA6 waste and the absence of a justified basis for distributing burdens between the primary and recycling life cycles, approaches involving economic or physical allocation are not suitable for this study. We therefore selected the cutoff approach,^44^ in which recycled PA6 is considered burden-free at the point where it enters the recycling life cycle, consistent with the polluter-pays principle.^45^ This aligns with Ekvall et al.,^46^ who compared 12 allocation approaches and found that the cutoff method, one of the most widely used recycling studies,^41,44,47^ is explicit, well-justified, and relevant for decision-making.

##### 2.3.1.2 System Boundaries

Building on this, the system boundaries define where the primary life cycle ends and the recycling life cycle begins. While some studies attribute the impacts of waste collection and sorting to the first life cycle,^48^ most assign them to the recycling life cycle.^21,41,44,45^ In our study, we define system boundaries to encompass not only the chemical depolymerization process but also the upstream steps required to prepare PA6 waste for recycling, including collection, sorting and prepurification. This ensures consistency with the cutoff principle^45,49^ and Environmental Product Declaration (EPD) recommendations,^46^ allowing for a fair and transparent comparison across the four chemical recycling pathways, whose differences in CL yields directly affect the required PA6 input for the production of 1 kg of recycled CL, and therefore the associated upstream burdens. This choice also supports a consistent comparison with linear end-of-life pathways (e.g., incineration), because chemical recycling typically requires more intensive sorting.

Figure 1 provides an overview of the system boundaries, covering all stages from the transportation (collection), sorting, and prepurification of PA6 waste to its chemical recycling to CL, ending at the factory gate. The figure also summarizes how uncertainty was handled in the model (see Section 2.3.4). Alternative background data sets were treated as discrete parameters. Discrete parameters and selected continuous parameters were varied in the sensitivity analysis and sampled in the Monte Carlo uncertainty analysis to derive uncertainty bounds around the base case.

**Figure 1:**
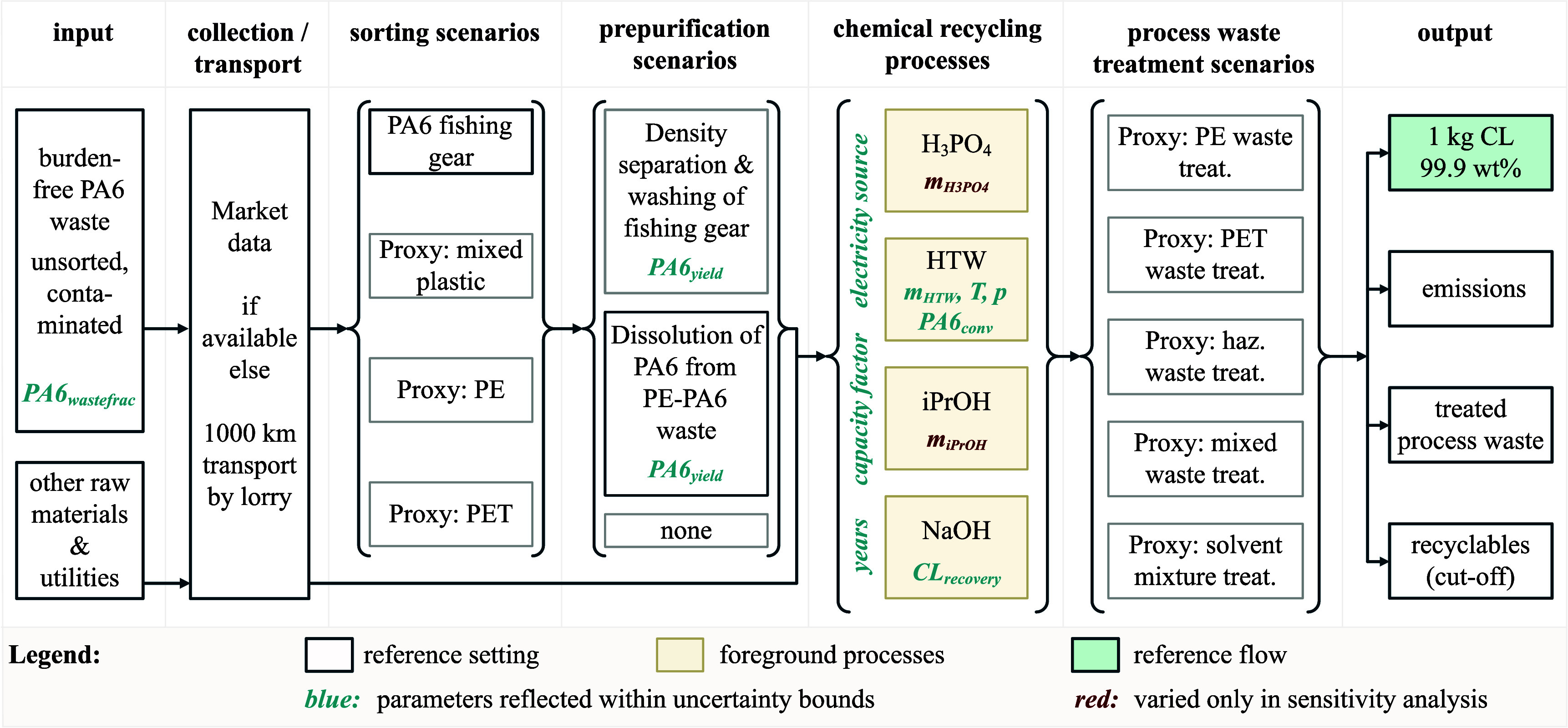
Simplified representation of the system boundaries and modeling framework. The scheme includes all relevant stages from contaminated PA6 waste to 1 kg recycled CL. Background process options (discrete parameters) are integrated into the Monte Carlo uncertainty analysis and sensitivity analysis. Reference data sets (base case) are marked by a blue frame. Continuous process parameters shown in blue are sampled in the Monte Carlo uncertainty analysis and sensitivity analysis, whereas red parameters represent case study variations considered only in the sensitivity analysis.

To reflect the variability in real-world PA6 waste streams, we model a flexible waste treatment cascade incorporating multiple plausible sorting and purification routes from the literature, as well as a variable fraction of PA6 in the mixed waste (parameter PA6_wastefrac_: 0.3–0.9). This improves LCA comparability relative to typical single-composition approaches.^19^ The reference data set for sorting is based on Schneider et al.,^50^ describing the sorting and shredding of fishing gear containing PA6. After sorting, additional purification may be required depending on feedstock quality. For this, we include two PA6-specific data sets: dissolution, using inventories from Costamagna et al.,^18^ and density-based separation and washing, based on industrial data reported by Schneider et al.^50^ PA6 wastes can contain pigments, fillers (e.g., CaCO_3_, glass fiber), flame retardants, and other additives such as stabilizers or plasticizers. In selective PA6 dissolution, these nontarget constituents, scaling with the non-PA6 share of the feed (PA6_wastefrac_), remain in the insoluble fraction and are removed before depolymerization as a single lumped reject stream sent to waste treatment (parameter waste treat.). According to literature, these stages yield PA6 fractions pure enough to substitute for virgin material,^50–53^ with minor PA6 losses accounted for by the PA6 yield parameter (PA6_yield_). Further details on all background scenarios are provided in Supporting Information Section B.2.

The purified PA6 waste stream enters the simulated chemical recycling processes (Section 3.1). Since all processes share the same downstream use of CL (e.g., PA6 production or disposal), extending the boundary beyond the factory gate would not provide additional insights. However, process waste generated during chemical recycling and its treatment (e.g., incineration) are included within the system boundaries, in accordance with the polluter-pays principle.^45^ The choice of the incineration data set depends on the process: for the solvent-free alkaline and acidic hydrolysis routes, hazardous waste treatment of PA6 and residual NaOH or H_3_PO_4_ is assumed. For the HTW and iPrOH processes, variable proxies for plastic waste incineration (mixed plastic waste, PE, and PET) are applied in the Monte Carlo simulation, with mixed plastic waste used as the reference. Incineration with energy recovery introduces multifunctionality, which is often addressed through substitution.^54^ However, in this study, we do not apply system credits for avoided impacts, such as energy recovery from incinerated waste streams, as such credits can lead to double-counting of recycling benefits, are inconsistent with the attributional LCA objective, and do not align with the cutoff approach.^20,41^

##### 2.3.1.3 Parameters

Detailed definitions, reference settings, and variation ranges for all parameters are provided in Supporting Information Section B.1. The main general parameters (highlighted in Figure 1) include plant lifetime, capacity factor, and electricity source (German electricity mix or wind-based). Waste-specific parameters comprise the PA6 fraction in the input waste stream, the type of collection and sorting system, prepurification status (i.e., whether a prepurification step is included or not), the prepurification method (dissolution vs density separation), and purification yield (the mass of purified PA6 obtained per mass of input waste).

Chemical recycling processes are modeled using their respective optimal conditions from the literature, including parameters such as temperature (T), conversion (PA6_conv_), pressure (p), reaction agent amount (m), and CL recovery. For the iPrOH and H_3_PO_4_ processes, the type of reaction agent does not vary in the literature, but we assessed its influence in the sensitivity analysis to identify potential bottlenecks.

#### 2.3.2 Inventory Analysis

The foreground chemical recycling processes, as detailed in Section 3.1, are illustrated with comprehensive process schemes, equipment lists, stream summaries, and utility requirements in Supporting Information Section B.3. The background system, described in Section 2.3.1.2, is sourced either from the ecoinvent v3.9.1 database (“cutoff by classification”) or relevant literature data sets.^26,54^ Due to the absence of equipment-specific data sets in ecoinvent, material and energy requirements for process equipment were estimated based on detailed equipment design and supplemented with procedures for key construction materials from Castellanos-Beltran et al.^55^ Detailed calculations are provided in Supporting Information Section C.1, and full inventory tables are presented in Supporting Information Section C.3.

#### 2.3.3 Impact Assessment

For this study, we chose the Environmental Footprint (EF) impact assessment method 3.1 as it provides a comprehensive, standardized LCA framework developed by the European Commission.^56^ The results are given in relation to the functional unit (1 kg of recycled CL). Following Zampori and Pant^57^ and applications,^58,59^ we normalized all EF 3.1 midpoints and selected nine categories whose combined contribution accounted for at least 80% of the normalized total for all processes, thereby covering multiple environmental dimensions.^59^ The selected impact categories are described in Table 1. Expanded descriptions are provided in Supporting Information Section E.2.2, and the selection procedure is detailed in Section E.2.3.

**Table 1:** Selected EF 3.1 Impact Categories with Abbreviations, Units, and Short Descriptions.^56^

name	abbrev. & unit	description
Global warming potential	GWP [kg CO_2_-eq]	aggregates greenhouse-gas emissions to CO_2_-equivalents using GWP100, which compares the time-integrated radiative forcing over 100 years of a 1-kg pulse emission of each gas to that of CO_2_.
freshwater ecotoxicity	FET [CTUe]	expresses the potential for emissions to exert toxic pressure on freshwater ecosystems, expressed as the potentially affected fraction of species integrated over time and volume per kilogram emitted.
freshwater eutrophication potential	EP [kg P-eq]	expresses the degree to which nutrient emissions in Europe reach inland waters and drive eutrophication, reported as phosphorus-equivalents per kilogram emitted.
carcinogenic human toxicity	HTC [CTUh]	expresses the potential increase in population cancer cases per unit mass emitted under generic, nonsite-specific conditions.
noncarcinogenic human toxicity	HTNC [CTUh]	expresses the potential increase in population noncancer cases per kilogram emitted under generic, nonsite-specific conditions.
Ionizing radiation on human health	IR [kBq U235-eq]	expresses the potential human-health impact of nonaccidental radionuclide releases as exposure efficiency relative to uranium-235 under nonsite-specific exposure assumptions.
Land use	LU [-]	expresses the soil-function deficit from land occupation and transformation via a dimensionless soil-quality index aggregating biotic production, erosion resistance, mechanical filtration, and groundwater recharge relative to a natural reference.
Resource use, minerals and metals^a^	ADP [kg Sb-eq]	also called abiotic depletion potential, expresses the long-term depletion potential of mineral and metal extraction using characterization factors that scale with annual production relative to ultimate reserves, reported as antimony-equivalents.
Water use^a^	WDP [m^3^ world-eq deprived]	also called water deprivation potential, expresses how consumptive freshwater use contributes to local water scarcity, expressed as consumed m^3^ multiplied by a dimensionless regional scarcity factor relative to a world-average basin.

a The results of this environmental impact indicator shall be used with care as the uncertainties of the results are high and as there is limited experience with the indicator.

#### 2.3.4 Interpretation

##### 2.3.4.1 Uncertainty and Sensitivity Analysis

As recommended by ISO 14040 ^39^, we conducted a sensitivity analysis to assess the robustness of our findings and quantify the uncertainty margins for key parameters (shown in Figure 1). Using the lca-algebraic Python package, we systematically evaluated the influence of discrete (different data sets) and continuous parameters (process variables) by varying them individually and simultaneously.^34^ We used one-at-a-time parameter variation to assess the maximum deviation from a fixed reference state (Section 3.3), and applied Monte Carlo simulations to provide uncertainty ranges (error bars) and probability distributions for each impact category.

##### 2.3.4.2 Limitations

The study’s limitations include the scarcity of background data for the PA6 waste market, collection and sorting systems, and the variability of waste compositions. As described in Section 2.3.4.1, this limitation is fully addressed by reporting uncertainty bars, ensuring that calculations are applicable to a range of real-world waste streams, rather than being limited to a single waste type.

Another limitation is that the required PA6 purification level may differ between the chemical recycling pathways. As a conservative estimate, our base case reference scenario includes all necessary prepurification steps required to purify PA6 prior to chemical recycling. To capture the possibility that some chemical recycling pathways may tolerate more contaminated waste while others require extensive purification, we vary both the inclusion/exclusion and the prepurification methods to generate the uncertainty bars. As a result, the model may yield broader uncertainty ranges, but this ultimately strengthens the robustness and generalizability of the assessment.

We acknowledge potential codissolution of certain additives or colorants during dissolution. We do not model additive-specific chemistry, catalyst poisoning, or decomposition products. These gaps may shift absolute burdens but are unlikely to change relative route rankings.

The different reported chemical recycling process conditions are also addressed through the parameter analysis. By varying the parameters based on literature-reported conditions, we capture this uncertainty, ensuring that experimental discrepancies are accounted for. However, we note that unit operations are sized by engineering heuristics and separations and purities reflect design targets, not plant measurements.

For the CL product, a conservative purity of 99.9 wt % is assumed, given limited evidence on additive carry-over and potential decomposition products. Achieving purities above 99.9 wt % typically requires additional ion-exchange, crystallization, and washing steps.^60,61^ If future data on the waste composition and reactor outlet streams become available, these additional purification steps could be incorporated. However, they are not considered relevant at this comparative stage.

One limitation of applying LCIA methods is the difficulty of interpreting trade-offs between different impact categories. To address this, we report and discuss all selected impact categories in the results, while also considering differences in their methodological robustness (see Supporting Information Section E.2.2). Lower-robustness categories (HTC, HTNC, LU, ADP, WDP)^62^ are interpreted with caution. To evaluate the robustness of our conclusions with respect to the choice of LCIA method, we additionally report corresponding impact estimates from ReCiPe 2016 and TRACI v2.1, confirming the qualitative ranking of chemical recycling routes (see Supporting Information Section D.2).

##### 2.3.4.3 Absolute Benchmarking of Chemical Recycling Routes Against Fossil-Based Caprolactam and Climate Targets

To move beyond conventional relative comparisons and contextualize the environmental performance of each pathway, we compared the GWP of each route to fossil-based CL and benchmarked results against an absolute carbon budget under a 1.5 °C scenario. Following the approach used in comparable studies, we applied the concept of the absolute sustainability ratio (ASR), which evaluates environmental impacts from our LCA relative to a defined environmental space.^63,64^ An ASR >1 indicates that CL production exceeds its allocated carbon budget, while ASR ≤1 suggests that production aligns with the 1.5 °C scenario.

The starting point is the annualized global carbon budget (CB­(t)), as reported by Hjalsted et al.^63^ who derived it from the IPCC Sixth Assessment Report,^65^ which defines the maximum cumulative CO_2_ emissions allowable to limit global warming to 1.5 °C by 2100. We then systematically downscaled this annual carbon budget in three key steps to define our environmental space: the annual carbon budget specifically allocated to CL production for PA6 polymerization (CB_CLforPA6_(t)) (eq 1).

First, 1.1% of CB­(t) was allocated to the plastic industry, reflecting its estimated share of global emissions according to Bachmann et al.^66^ Second, the share of PA6 was determined based on its predicted annual production volume (M_PA6_(t)) relative to the total predicted plastic production volume ( $\sum_{i}M_{Plastics}\left(t\right)$ ). Third, this annual carbon budget for PA6 production (CB_PA6_(t)) was further refined to reflect only the portion allocated to CL production, which is specifically used for PA6 polymerization, resulting in the final CB_CLforPA6_(t), using an emission-based allocation factor (β_GWP_) and a conversion factor (α_CLtoPA6_). The detailed calculation procedure, including values, references, and assumptions, can be found in Supporting Information Section E.3.

1 $$CB_{CLforPA6}\left(t\right)=CB\left(t\right)\cdot 0.011\cdot \frac{M_{PA6}\left(t\right)}{\sum_{i}M_{Plastics}\left(t\right)}\cdot \frac{\beta_{GWP}}{\alpha_{CLtoPA6}}$$

Building on this, ASR_CLforPA6_(t) (eq 2) was used to assess whether the annual GWP of CL production from PA6 depolymerization (GWP_CL,annual_(t)) remained within its allocated annual carbon budget under the +1.5 °C scenario.

2 $$\text{AS}R_{CLforPA6}\left(t\right)=\frac{{GWP}_{CL,annual}\left(t\right)}{CB_{CLforPA6}\left(t\right)}$$

## 3 Results and Discussion

### 3.1 Foreground System: Modeled Chemical Recycling Processes

Figure 2 shows the foreground systems simulated in Aspen Plus based on our previous work with additional detail and literature-based adaptations.^4,29,67^

**Figure 2:**
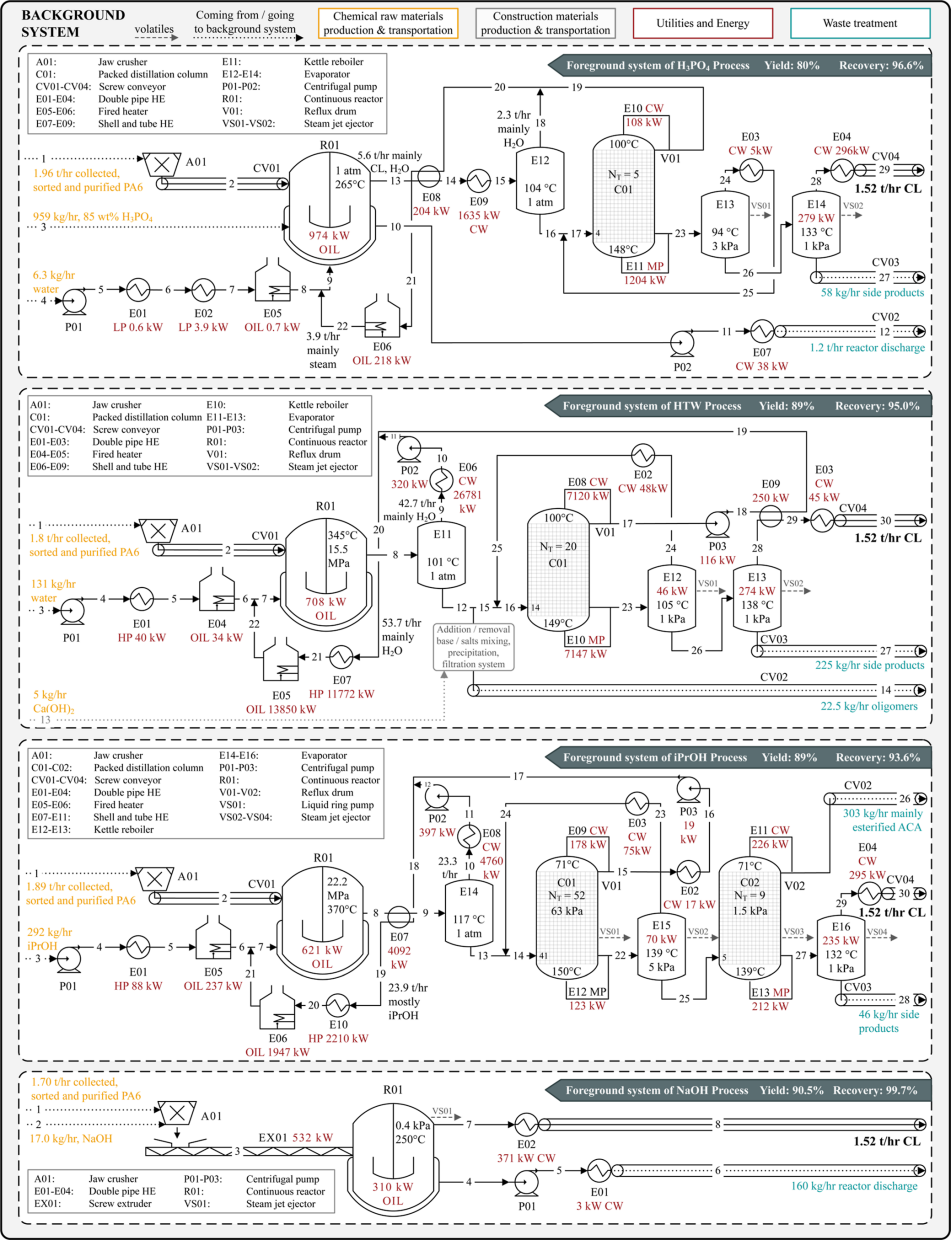
Schematic process flow diagrams of the four simulated remonomerization processes, including system boundaries and connections to background systems.

It can be seen that the purified PA6 stream enters the foreground system for shredding and subsequent depolymerization, where CL is produced based on literature-reported yields for each pathway (Supporting Information Section B.1). As detailed in Sections 2.3.1 and 2.3.4, PA6 waste collection, sorting, and purification are modeled as background processes, with pure PA6 assumed as input to chemical recycling in line with the waste hierarchy and literature showing that available prepurification steps (e.g., dissolution or density separation) can yield near-virgin quality material.^50–52^

Purification is challenging due to CL’s high boiling point and heat sensitivity, requiring temperatures below 150 °C to prevent unwanted repolymerization.^68,69^ Maintaining low distillation pressures while preventing CL crystallization adds further complexity.^60,70^ For the HTW and iPrOH processes, CL purification includes solvent recovery, typically achieved by flash evaporation immediately after the reactor.^67,71^ For the NaOH and H_3_PO_4_ processes, waste streams containing their respective reaction agents are generated. In HTW-based depolymerization, where dimers, trimers, oligomers, and side products remain in solution, a flash operation partly leads to oligomer precipitation.^4,67^ Aminocaproic acid (ACA) and other side products can be simultaneously removed by introducing calcium hydroxide to form salts.^72^ This additional step is unnecessary in acidic hydrolysis, as ACA is directly separated in the reactor via salt formation with phosphoric acid.^5^ In the alcoholysis process, the formation of ACA is not expected, as an ACA derivative is recovered as an ester intermediate.^73^

Following flash evaporation, it is important to keep water content in a range of 0.5–6% by weight in the bottom stream of the distillation at atmospheric pressure.^60,70^ Under these conditions, the distillation temperature at the column top remains above CL’s melting point, while the bottom temperature stays below 150 °C. To further remove residual water (HTW process) or iPrOH, an additional vacuum evaporator is required to recycle the light components back to the distillation column.^67^ The distillate from both the column and the evaporator, primarily water (HTW process) or iPrOH, can be recycled, provided no low-boiling side products are present. In the iPrOH process, a further distillation step is required to separate the esterified compound. This study excludes the removal of additional low-boiling components, acidic impurities in the H_3_PO_4_ process, and the need for purge streams due to low relevance and limited data of potential side products. In the final stage of all three processes, CL is distilled to purify it from high-boiling compounds retained in solution.^74^ To maintain the solubility of oligomers, a controlled amount of CL is retained in the bottom stream.^74^

The solventless process using NaOH, identified in our previous TEA as the most promising process, facilitates direct CL formation through chain-end backbiting (intramolecular cyclization).^4,75^ Consequently, direct vacuum distillation of CL from the reaction mixture produces a CL product stream that meets the purity requirements (>99.9 wt %), as recently confirmed experimentally.^76^

### 3.2 Contributions to Impact Categories for Each Process

Figure 3 illustrates the relative impact contributions of various utilities, raw materials and auxiliary systems in the selected environmental impact categories for the chemical recycling processes at the reference base case conditions.

**Figure 3:**
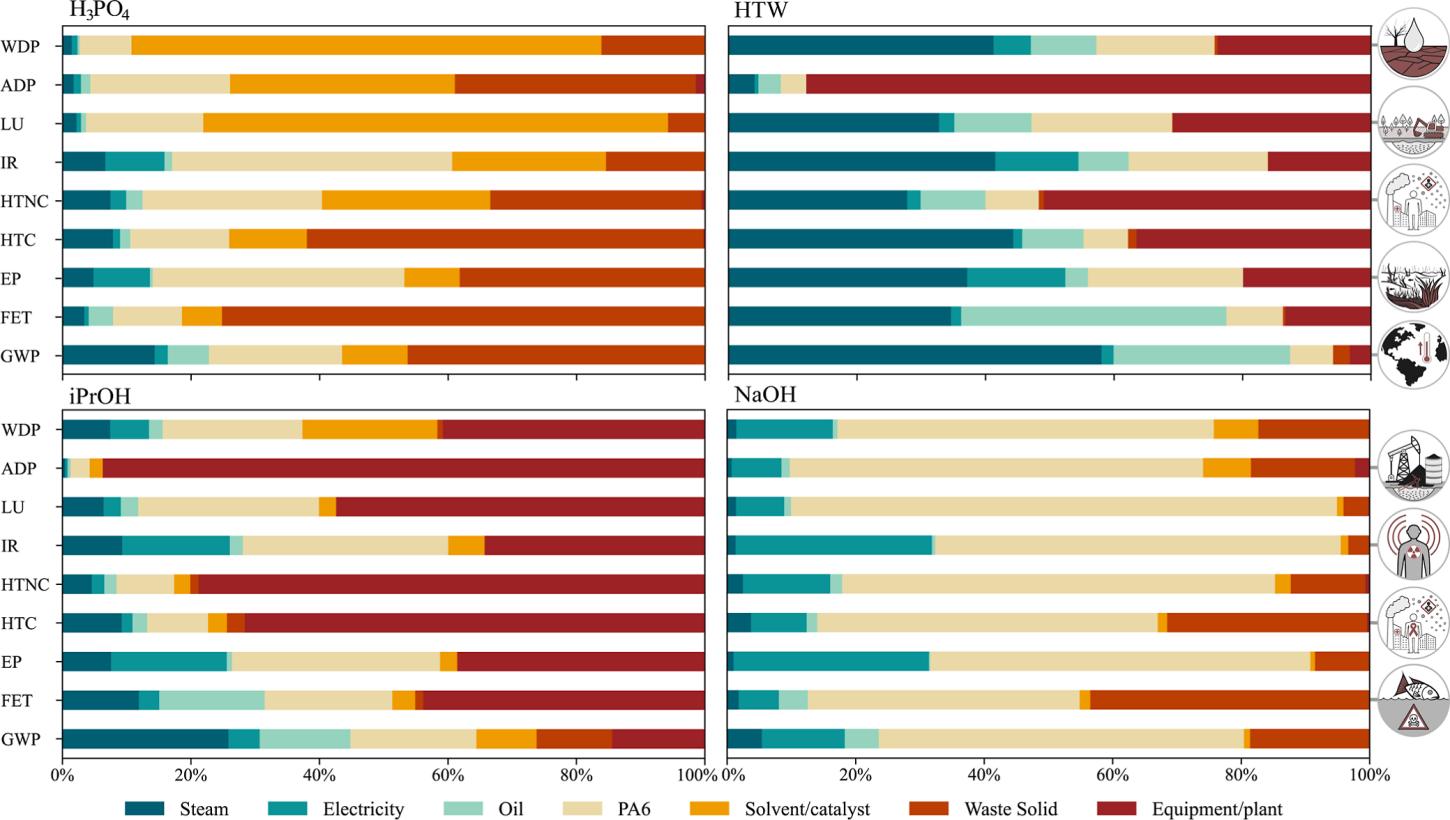
Percentage contributions of steam (for heat), electricity, oil, collected/sorted/pre-treated PA6 waste, solvent/catalyst use, waste treatment, and plant/equipment/auxiliary systems to nine environmental impact categories, scaled to 100%, for the four chemical recycling routes to CL: global warming potential (GWP), freshwater ecotoxicity (FET), freshwater eutrophication potential (EP), carcinogenic human toxicity (HTC), noncarcinogenic human toxicity (HTNC), ionizing radiation (IR), land use (LU), abiotic depletion potential (ADP), and water deprivation potential (WDP).

The iPrOH and HTW processes exhibit high utility-related impacts across categories due to their elevated temperatures, pressures, and large streamflow rates, leading to remarkable energy demands and the need for more robust equipment. Consequently, their considerable equipment and plant material requirements are particularly pronounced in the ADP and HTNC categories, with these contributions accounting for more than 80% of the ADP impacts. This is due to the extensive use of steel, aluminum, and rare metals required for electronic controls and wiring, as well as the release of toxic byproducts during their extraction, production, and maintenance.^77–79^

For the H_3_PO_4_ and NaOH processes, hazardous waste treatment is a contributor across all impact categories, with particularly high impacts in the H_3_PO_4_ process. As addressed in our recent work,^5^ H_3_PO_4_ reacts with ACA and base impurities, forming a nonrecoverable salt complex.^80^ Its consumption exceeds catalytic amounts, increasing both raw material demand and waste generation. Consequently, waste treatment contributes more significantly to the overall impacts of the H_3_PO_4_ process compared to the NaOH process. This is particularly evident in FET, where hazardous waste incineration byproducts pose increased risks to freshwater ecosystems.^81^

PA6 waste contributes noticeably to raw material impacts, primarily due to prepurification steps rather than sorting. This effect is particularly pronounced in the NaOH process, where the overall impacts of other variables are small. As expected, the HTW process shows no visible solvent contribution (water), whereas solvent use is essential in the other processes. In the H_3_PO_4_ process, the production of H_3_PO_4_ significantly contributes to WDP due to its high water demand for washing, cooling, and wastewater management, and to LU due to the extensive, long-term land occupation and soil function loss associated with phosphate rock mining and the stacking of phosphogypsum waste generated during acid production.^82,83^

As expected, utilities are generally major contributors to GWP. Even in the less energy-intensive alkaline process, utilities account for more than 20% of GWP due to combustion-related emissions and the coal-based share of the German electricity grid.^84,85^

Overall, consistent with our economic results,^4^ utilities and equipment dominate the impacts in the alcoholysis and hydrothermal processes, waste treatment is significant for the acidic hydrolysis process, and the PA6 feedstock is the key contributor in the solvent-free NaOH process.

### 3.3 Parameter Analysis

Figure 4 presents the parameter analysis, described in Section 2.3.4.2, showing the extent to which each parameter influences impact categories within its relevant valid range (see Supporting Information Section B.1). For H_3_PO_4_ and iPrOH, the variation in reagent amounts reflects case scenarios rather than true uncertainties (see red parameters in Figure 1).

**Figure 4:**
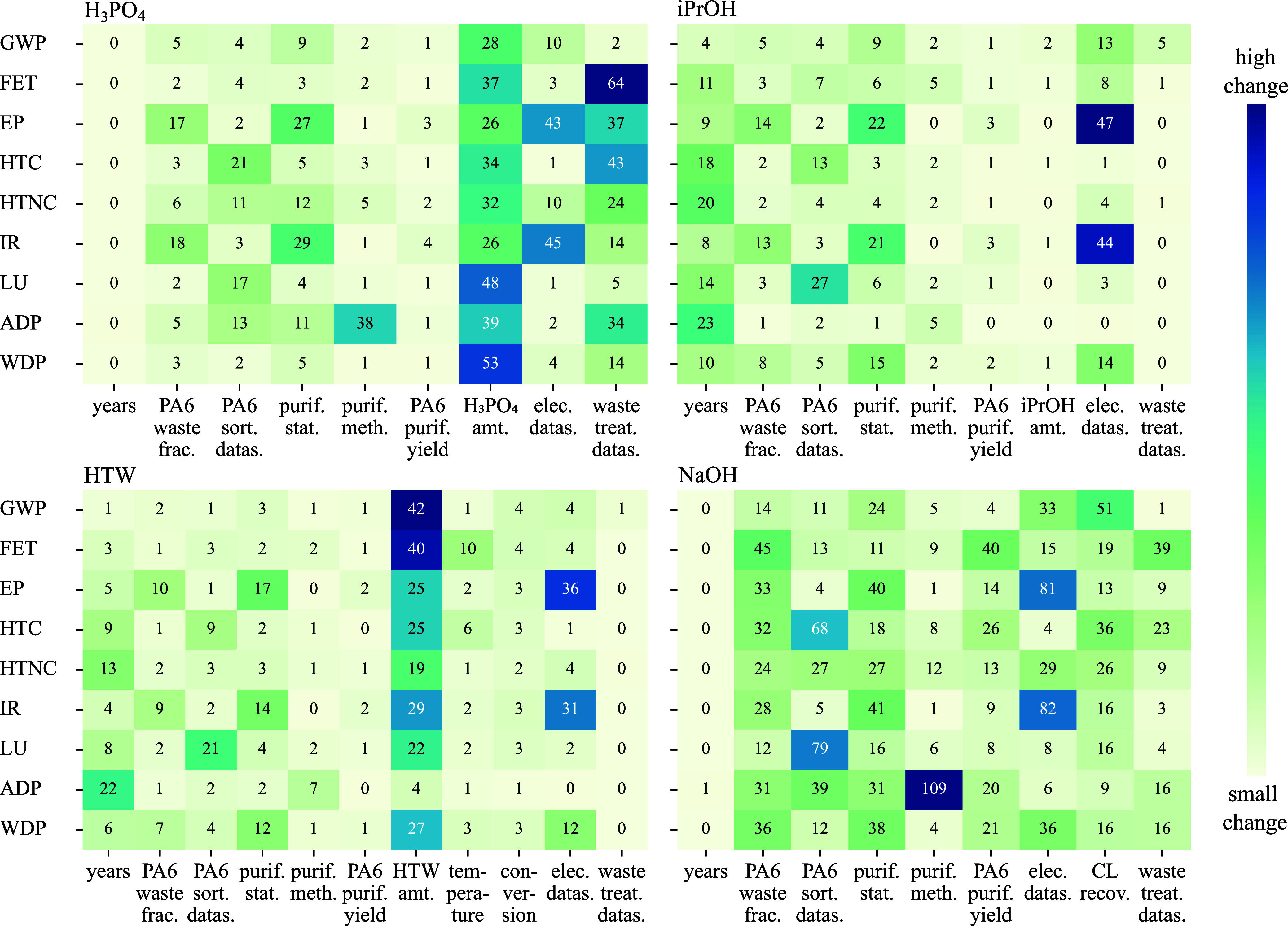
Parameter analysis of key process parameters for each chemical recycling pathway, evaluated across nine environmental impact categories: global warming potential (GWP), freshwater ecotoxicity (FET), freshwater eutrophication potential (EP), carcinogenic human toxicity (HTC), noncarcinogenic human toxicity (HTNC), ionizing radiation (IR), land use (LU), abiotic depletion potential (ADP), and water deprivation potential (WDP). The analysis shows the maximum percentage deviation in impacts when each parameter is individually varied from the reference state to its most extreme value within its valid range or to an alternative case scenario.

#### 3.3.1 Pretreatment of PA6 Waste

Unexpectedly, the amount of mass processed, reflected automatically through the parameter of PA6 content in the incoming waste stream (PA6 waste frac.), has only a medium influence, with burden increase for the H_3_PO_4_, HTW, and iPrOH processes staying below 20%. As expected, given the contribution of PA6 waste, its composition has a greater influence on the NaOH process, but variations are still mostly within 24–36% for six categories, and only 14% for GWP.

Regarding the choice of sorting data set (PA6 sort. datas.), there is a minor influence on most impact categories. However, for the NaOH process, this parameter shows higher sensitivities, particularly for LU, HTC, and ADP. In contrast, for the HTW and iPrOH processes, the effect remains below 7% for all categories except LU and HTC.

Including or excluding prepurification of PA6 waste (purif. stat.) has only a moderate effect on results for most categories, with EP and IR being most affected for all processes. The high electricity demand of the dissolution process amplifies EP through nutrient emissions from coal extraction and combustion.^86^ It also increases IR due to impacts from the nuclear energy lifecycle and the release of naturally occurring radioactive materials during coal extraction.^86^

In contrast, the choice of prepurification method (density separation or dissolution: PA6 purif. meth.) has a negligible impact across all categories for the iPrOH and HTW processes. For the NaOH and H_3_PO_4_ processes, it significantly affects only the ADP category, due to substantial NaCl use in density separation. This effect is more pronounced in these processes because their process material and equipment contributions to ADP are relatively small (see Section 3.2).

In conclusion, although the prepurification system for PA6 waste has only a limited influence in most processes, its impact is still substantial enough not to be overlooked. This contrasts with other literature, which states that prepurification has less impact on environmental performance and can often be excluded from system boundaries.^50^ However, while prepurification itself is not negligible, the specific choice of sorting data set and prepurification method has only a small effect on most impact categories, suggesting that our overall results are robust to these modeling choices.

#### 3.3.2 Process Waste Treatment

For the NaOH and H_3_PO_4_ processes, varying the process waste treatment data set (rather than fixing it as hazardous waste treatment) demonstrates the possible influence of milder reaction agents as a case study and does not reflect a real uncertainty. As shown, switching from hazardous waste to mixed plastic waste incineration would significantly reduce impacts in FET, EP, HTC, and ADP, especially in the acidic process.

Variations in the waste treatment data set of the alcoholysis and hydrothermal processes, where three different plastic incineration proxies (mixed plastic waste, PE, and PET) were used, have a negligible effect, with impact variations staying below 5%. The results suggest that, unless hazardous waste treatment is involved, the specific choice of proxy for PA6 incineration does not significantly influence the outcomes.

#### 3.3.3 Main Parameters Affecting the Acidic Process

The primary factors influencing the impacts of the acidic process are the quantity of H_3_PO_4_ used and the waste treatment option, both varied as deliberate scenario choices rather than treated as real uncertainties. Reducing the H_3_PO_4_ amount would lower waste generation, utility demand, and raw material use, thereby substantially decreasing WDP and LU. Interestingly, these findings align with our previous TEA, which recommended reducing waste treatment and raw material use by recovering the acid and preventing salt formation with ACA.^4,5^ The present study introduces a new insight: substituting H_3_PO_4_ with a less harmful acidic reagent could substantially reduce most impacts as the production of H_3_PO_4_ drives a large share of the overall burdens. However, no alternative process has been reported that achieves this reactant agent substitution while maintaining process functionality. Given its potential for direct CL recovery, future research should explore alternative acidic reagents or catalysts to enhance both environmental and economic performance by eliminating hazardous waste treatment and harmful production impacts.

#### 3.3.4 Main Parameters Affecting the Alkaline Process

Due to the generally low impacts of the NaOH process, the relative influence of individual parameters is more pronounced. The NaOH process enables direct CL recovery, yet this parameter has a substantial impact: GWP can increase by up to 51%. This outcome is expected, since any unrecovered CL must be offset by additional PA6 input, which is the primary contributor to the environmental burden of this process (see Section 3.2).

#### 3.3.5 Main Parameters Affecting the Alcoholysis and Hydrothermal Processes

Figure 4 also highlights that in the iPrOH and HTW processes, the electricity source and operating years are critical parameters due to higher pressures and increased material requirements for equipment, as described in Section 3.2. Notably, the strong influence of plant lifetime, a new insight compared to our TEA, indicates that the widespread LCA practice of assuming a fixed chemical plant construction data set from ecoinvent can lead to major inaccuracies, particularly for energy- and material-intensive processes.

The widely documented HTW process has varying literature-reported solvent-to-feed ratios, reactor temperatures, and PA6 conversion rates, all of which affect multiple impact categories. Notably, reducing the water-to-feed ratio from 30 ^87^ to 11 ^88^ significantly decreases utility demands and equipment sizes, leading to substantial reductions across all impact categories, with GWP decreasing by up to 42%. This reinforces the importance of incorporating a range of process conditions from the literature in LCA studies. In contrast, despite an iPrOH-to-PA6 waste input ratio of more than 13:1, the amount of iPrOH has a negligible effect across all categories, indicating limited potential for further improvement.

In summary, these results demonstrate that while certain parameters (such as prepurification, sorting, and plant equipment production) are often overlooked in LCA, they can meaningfully affect selected impact categories. Accurately capturing process- and parameter-specific sensitivities is therefore critical for robust environmental assessments of PA6 recycling technologies.

### 3.4 Comparison of the Processes

#### 3.4.1 Is Any Chemical Recycling Pathway Consistently Superior Across All Impact Categories and Uncertainties?

To assess whether robust conclusions can be drawn despite all variables and uncertainties, Figure 5 presents the environmental impact categories for each chemical recycling pathway, normalized to the pathway with the highest impact in each category. Error bars reflect the uncertainty of each process, as derived from the Monte Carlo simulation described in Sections 2.3.1.2 and 2.3.4.2. The complete uncertainty analysis, including impact probability distributions and violin plots for each impact category and process, is provided in Supporting Information Section D.

**Figure 5:**
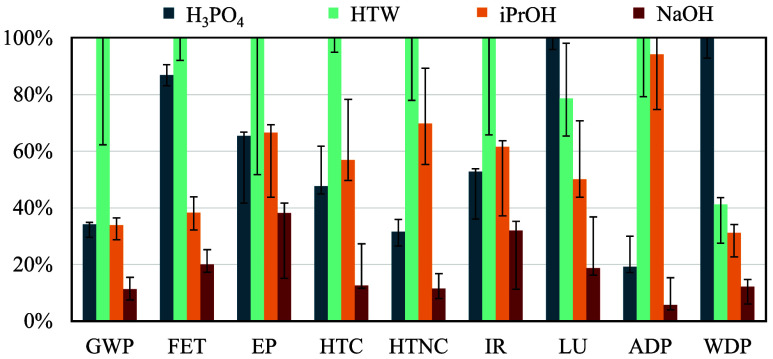
Environmental impact categories-global warming potential (GWP), freshwater ecotoxicity (FET), freshwater eutrophication potential (EP), carcinogenic human toxicity (HTC), noncarcinogenic human toxicity (HTNC), ionizing radiation (IR), land use (LU), abiotic depletion potential (ADP), and water deprivation potential (WDP)-for each chemical recycling pathway, normalized to the pathway with the highest impact in each category. Error bars indicate uncertainties derived from the Monte Carlo analysis.

While overlaps exist across many impact categories, several clear conclusions can be drawn. The hydrothermal HTW process tends to exhibit the highest impacts across some categories, even under the best conditions, such as the exclusion of PA6 prepurification, the lowest solvent-to-feed ratio, wind-based electricity supply, the highest conversion rates, and the lowest reactor temperatures. In contrast, the NaOH process consistently shows the lowest environmental burdens, even under the least favorable conditions, aligning with our economic results.^4^

Some findings, however, are unexpected. Based on much lower energy requirements of the acidic process, it would be anticipated that the H_3_PO_4_ process would have lower GWP than the iPrOH pathway. Yet, the LCA shows that iPrOH and H_3_PO_4_ have comparable impacts in GWP and two other categories (EP and IR). Conversely, although the alcoholysis process shows a much better cost and profitability profile in the TEA,^4^ it has significantly higher impacts in three categories (HTC, HTNC, and ADP), driven not by utility usage, but by elevated equipment and plant material requirements. While the human toxicity categories are considered highly relevant,^89^ they are also among the least robust EF 3.1 indicators because they are strongly influenced by modeled long-term emissions from landfills and mining deposits.^90^

Unexpectedly, the H_3_PO_4_ process has the greatest impact in the LU and WDP categories. These impacts are not caused by hazardous waste treatment, as suggested by the TEA,^4^ but mainly by the environmental burden of H_3_PO_4_ production itself. This confirms that H_3_PO_4_ production not only contributes significantly within its process (Sections 3.2 and 3.3) but also drives these categories in the comparative assessment. However, while the water- and land use indicators are considered environmentally relevant,^89^ they are not highly robust, which is similar for the HTC and HTNC indicators.^90^ Consequently, considering the divergent performance across impact categories and the uncertainties associated with these indicators, no clear distinction can be made between the H_3_PO_4_ and iPrOH processes.

#### 3.4.2 Can Chemical Recycling Outperform Fossil-Based Monomer Production or Plastic Waste Incineration?

Since GWP is a key indicator for assessing climate change mitigation potential,^40^ Figure 6 presents the absolute GWP for the four chemical recycling processes to 1 kg recycled CL, for 1 kg of fossil-based CL, as well as for the incineration of the route-specific amount of PA6. The GWP for conventional fossil-based CL is based on Hong et al.,^91^ who detailed the environmental impacts of the linear production route: the oxidation of cyclohexane to cyclohexanone, its conversion to cyclohexanone oxime using hydroxylamine, and the subsequent Beckmann rearrangement to produce CL. The Supporting Information Section D.5 also provides surrogate equations, enabling GWP estimation for different parameter values, such as variable feedstock composition or solvent amounts across the four chemical recycling processes.

**Figure 6:**
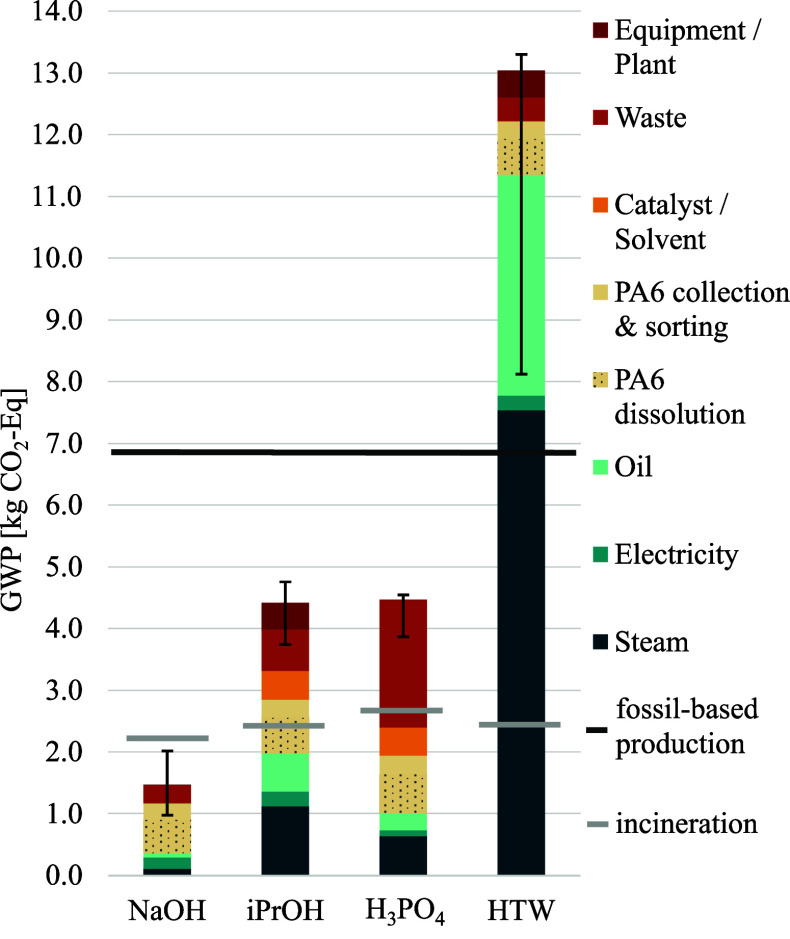
Absolute global warming potential (GWP) contributions of the four chemical recycling pathways. The GWP of fossil-based production of 1 kg of CL and the impact of incinerating the route-specific PA6 amount are also shown.

The results indicate that the NaOH, H_3_PO_4,_ and iPrOH chemical recycling processes have lower GWP than fossil-based CL production from benzene, even when accounting for uncertainties. Even for the upper uncertainty bound, the NaOH process remains below one-third of the GWP of fossil-based CL production. Similarly, the H_3_PO_4_ and iPrOH processes exhibit GWP values that are about 35% lower than those of the fossil-based route. These findings support the conclusions of Hong et al.,^91^ who recommended improving the environmental performance of CL production by recovering CL from nylon filament yarn. They also align with Meys et al.,^92^ who show that both mechanical and chemical recycling significantly lower greenhouse gas emissions compared to the conventional linear carbon production pathway.

The only exception is the chemical recycling process using HTW, which exceeds the GWP of fossil-based CL production even under its best-case scenario. With utilities accounting for over 85% of its GWP, achieving environmental competitiveness would likely require more extensive heat integration. However, the findings from the NaOH process indicate that operating at lower temperatures, reducing reaction agent-to-feed ratios, and integrating separation within the reaction zone are even more critical factors for minimizing environmental impacts.

Incineration of the amount of PA6 required to produce 1 kg of CL of the respective process does not yield any valuable chemical product, making direct attributional comparison with chemical recycling unfavorable. Yet, remarkably, the NaOH process still achieves a lower GWP than incineration. This aligns with studies showing that substituting virgin plastics with recycled plastics results in greater greenhouse gas emission reduction than incineration.^19,93^ Since the GWP for combining virgin production with incineration is higher than that of three chemical recycling processes, the results strongly suggest that substituting fossil-based CL with chemical recycling can significantly reduce emissions. To account for inefficiencies, our ongoing work explores an integrated approach combining biobased and fossil-based production, chemical and mechanical recycling within a circular PA6 framework.^94^

### 3.5 Can Chemical Recycling of PA6 Meet the Absolute Carbon Budget?

The previous section demonstrates that chemical recycling has the potential to significantly reduce environmental impacts compared to conventional fossil-based CL production, emphasizing its role in the transition to a circular economy for nylons. However, when contextualized within a carbon budget specifically allocated to CL production for PA6 polymerization under a 1.5 °C scenario, assuming 100% of PA6 is chemically recycled, none of the chemical recycling pathways achieve absolute environmental sustainability across 2025, 2030, and 2050 (see Supporting Information Section D.6). For 2025 and 2030, the ASR for the most favorable chemical recycling process ranges from 1.21 to 2.50 and from 1.93 to 3.98, respectively. Although Bachmann et al.^66^ employ a planetary boundaries approach while we apply a carbon budget framework, both studies agree that achieving absolute environmental sustainability remains challenging.

As the NaOH process comes closest to meeting the carbon budget, future research should prioritize the scale-up of solvent-free depolymerization routes that integrate reaction and monomer purification, a direction also pursued in recent advances.^95,96^ Since this process is sensitive to both PA6 waste prepurification and process waste management, lowering feedstock purity requirements, replacing NaOH with a recyclable catalyst, and improving CL recovery could significantly reduce impacts. Ultimately, only processes that achieve GWP values below 0.61 and 0.38 kg CO_2_-eq per kg CL for 2025 and 2030, respectively, will be compatible with the carbon budget, setting a clear threshold. This underscores the urgent need for coordinated research, technological innovation, and policy action in both chemical recycling and lower-temperature pathways such as solvent-based dissolution,^52,53,97^ to achieve truly circular and climate-compatible polyamide value chains.

## Supplementary Material



## Notes

Supporting Information Section A compiles the scalar and temperature-dependent property data and binary interaction parameters; Section B details the process models, background systems, and sensitivity/uncertainty parametrization; Section C reports the full inventory calculations (equipment, utilities, and LCI tables); Section D provides additional results (LCIA cross-method comparisons, uncertainty distributions/violin plots, surrogate equations, and carbon-budget evaluation); Section E documents the underlying methodology and assumptions; Section F lists abbreviations and nomenclature (PDF)
