# Supplementary material for: Parametric Life Cycle Assessment of Chemical Recycling of Nylon‑6 to Caprolactam
Source: Environ Sci Technol. 2026 Feb 16;60(8):6770–83. doi: 10.1021/acs.est.5c16101 (PMC12961928; doi:10.1021/acs.est.5c16101)
Supplement: Supplementary file 1 [file es5c16101_si_001.pdf]

SUPPORTING INFORMATION FOR:

Parametric Life Cycle Assessment of Chemical Recycling of Nylon 6 to Caprolactam

Ann-Joelle Minor[a,b*], Ruben Goldhahn[b], Caroline Ganzer[b], Michaël Lejeune[c,d], Liisa Rihko-Struckmann[b*], Kai Sundmacher[a,b]

[a] Chair for Process Systems Engineering, Otto von Guericke University Magdeburg, Universitätsplatz 2, 39106 Magdeburg, Germany

[b] Department Process Systems Engineering, Max Planck Institute for Dynamics of Complex Technical Systems, Sandtorstraße 1, 39106 Magdeburg, Germany

[c] Sustainability in Manufacturing and Life Cycle Engineering Research Group, School of Mechanical and Manufacturing Engineering, The University of New South Wales, Sydney 2052, Australia

[d] Australian Research Council, Training Centre for the Global Hydrogen Economy, Sydney 2052, Australia

***Corresponding Authors**

Ann-Joelle Minor – Otto von Guericke University Magdeburg, Chair for Process Systems Engineering, Universitätsplatz 2, Magdeburg 39106, Germany; orcid.org/0000-0001-5673-8221; Email: aminor@mpi-magdeburg.mpg.de

Liisa Rihko-Struckmann – Max Planck Institute for Dynamics of Complex Technical Systems, Department Process Systems Engineering, Sandstorstr. 1, 39106 Magdeburg, Germany; orcid.org/0000-0003-0222-7236; Email: rihko@mpi-magdeburg.mpg.de

Number of Pages: 84

Number of Tables: 41

Number of Figures: 26

Number of References: 63

Table of Contents

TABLE SUMMARY 4

FIGURE SUMMARY 6

A PROPERTY & THERMODYNAMIC DATA COLLECTION 9

A.1 SCALAR PROPERTIES 9
A.2 TEMPERATURE-DEPENDENT PROPERTIES 9
A.2.1 Experimental Data 9
A.2.2 Polynomials and Parameters 10
A.3 BINARY INTERACTION PARAMETERS 11

B PROCESS AND PARAMETER DESCRIPTIONS 12

B.1 VARIED PARAMETERS FOR SENSITIVITY ANALYSIS, DISTRIBUTIONS AND UNCERTAINTY RANGES 12
B.2 BACKGROUND SYSTEMS 13
B.2.1 Assumed Sorting Systems 13
B.2.1.1 Reference - Sorting of Fishing Gear containing PA6 13
B.2.1.2 Proxy - Sorting of Mixed Plastic Waste 14
B.2.1.3 Proxy - Sorting of PE and PET 14
B.2.2 Assumed Pre-Purification Systems 14
B.2.2.1 Density Separation and Washing of Fishing Gear containing PA6 14
B.2.2.2 Reference - Dissolution of PA6/PE Packaging Waste 15
B.2.3 Proxy - Incineration of Process Waste 15
B.3 FOREGROUND SYSTEM – CHEMICAL RECYCLING PROCESSES 16
B.3.1 Technology Readiness Level 16
B.3.2 Process Flow Schemes, Stream Summaries and Equipment Summaries 17
B.3.2.1 H_3PO_4 Process 17
B.3.2.2 HTW Process 20
B.3.2.3 iPrOH Process 23
B.3.2.4 NaOH Process 26

C INVENTORY ANALYSIS 27

C.1 EQUIPMENT 27
C.1.1 Jaw Crusher 27
C.1.2 Reactor 27
C.1.3 Distillation Column Vessel and Reflux Drum 28
C.1.4 Filter 31
C.1.5 Heat Exchanger 31
C.1.6 Pumps 32
C.1.7 Screw Conveyor 32
C.1.8 Extruder 32
C.1.9 Vacuum System 33
C.1.10 Piping, Instrumentation and Control 33
C.1.11 Chemical Factory 33
C.1.12 Raw Materials 33
C.2 UTILITY CALCULATIONS 33
C.2.1 Equipment Utility Requirements 33
C.2.1.1 Steam Jet Ejector 33
C.2.1.2 Liquid Ring Pump 34
C.2.1.3 Jaw Crusher 34
C.2.1.4 Screw Conveyor 34
C.2.1.5 Reactor Turbine Agitator 34

C.2.1.6 Extruder 34
C.2.2 Conditions 34
C.2.2.1 Cooling Water 34
C.2.2.2 Steam 35
C.2.2.3 Fuel Oil No2 35
C.2.2.4 Dowtherm A 35
C.3 INVENTORY TABLES 36
C.3.1 Background System - Collection and Sorting of PA6 Waste 36
C.3.2 Background System - Prepurification of PA6 Waste 37
C.3.3 Foreground System - Chemical Recycling of Collected, Sorted and Pre-Purified PA6 Waste 38
C.3.3.1 H_3PO_4 Process 38
C.3.3.2 HTW Process 40
C.3.3.3 iPrOH Process 42
C.3.3.4 NaOH Process 43
D RESULTS AND INTERPRETATIONS 45
D.1 ENVIRONMENTAL IMPACTS 45
D.2 IMPACTS FOR DIFFERENT LCIA METHODS: EF 3.1, RECIPE 2016, TRACI 2.1 46
D.3 PROBABILITY DISTRIBUTIONS 48
D.4 VIOLIN CHARTS 52
D.5 SURROGATE GWP EQUATIONS AS FUNCTIONS OF KEY PARAMETERS 56
D.5.1 H_3PO_4 Process 56
D.5.2 HTW Process 59
D.5.3 iPrOH Process 62
D.5.4 NaOH Process 64
D.6 ABSOLUTE EVALUATION OF GWP AGAINST A CARBON BUDGET 67
E METHODOLOGY 69
E.1 VARIABLE MIXED PLASTIC WASTE MODELING 69
E.2 LIFE CYCLE ASSESSMENT 69
E.2.1 Goal and Scope Definition 69
E.2.2 Description of Impact Categories 71
E.2.3 Choice of Impact Categories 74
E.3 ABSOLUTE EVALUATION OF GWP AGAINST A CARBON BUDGET 75
E.3.1 General Approach 75
E.3.2 Assumptions 75
E.3.3 Formulas 76
E.4 PROCESS LEVEL 78
E.4.1 Near-Optimal Temperature Approaches, Pressure Drops and Temperature Changes 78
E.4.2 Heat Transfer Coefficients 78
F ABBREVIATIONS & NOMENCLATURE 79
REFERENCES **81**

Table Summary

Table A 1: Scalar properties of PA6 taken from the Polymer Handbook [2]. 9
Table A 2: Liquid and solid heat capacity data of PA6 sourced from [2,3]. 9
Table A 3: parameters for the polynomial of ideal gas heat capacity 10
Table A 4: parameters for the DIPPR equation for liquid heat capacity 10
Table A 5: parameters for the DIPPR equation for solid heat capacity 10
Table A 6: NRTL parameters for the components and their source. The parameters D_{IJ}, E_{IJ}, E_{JI}, F_{IJ}, and F_{JI} are 0 and the unit for temperature is in Kelvin. 11
Table A 7: Equipment dimension calculation of horizontal vessels such as reactors according to Seader et al. [20,22]. 27
Table A 8: Equipment dimension calculation of the distillation columns [20,22]. 28
Table A 9: Equipment dimension calculation of the reflux drum [20,22]. 30
Table A 10: Heat exchanger dimension calculation and material requirement calculation [20,22]. 31

Table B 1: Overview of the parameters, including their type, reference setting, and description of each parameter. 12
Table B 2: Varying investigated reaction temperatures, pressures and times with different optimum conditions reported by the literature for the HTW process 13
Table B 3: stream table of the H_3PO_4 process including information on phases, temperatures, pressures, mass flows, mass fractions and enthalpy flows of the streams. 17
Table B 4: stream table of the HTW process including information on phases, temperatures, pressures, mass flows, mass fractions and enthalpy flows of the streams. 20
Table B 5: stream table of the iPrOH process including information on phases, temperatures, pressures, mass flows, mass fractions and enthalpy flows of the streams. 23
Table B 6: stream table of the NaOH process including information on phases, temperatures, pressures, mass flows, mass fractions and enthalpy flows of the streams. 26
Table B 7: Characteristics of cooling water that is taken as utility in this project 34
Table B 8: Steam classes that are taken as utilities for this project, the absolute pressure, temperature and evaporation enthalpy 35
Table B 9: Characteristics of fuel oil No.2 that is taken as utility in this project for process temperatures until 300°C [23] 35
Table B 10: Characteristics of Dowtherm A that is taken as utility in this project for process temperatures until 350°C 35

Table C 1: Equipment dimension and material requirement calculation of the extruder. 32
Table C 2: REFERENCE SWITCH ACTIVITY - Inventory table listing activities, locations, quantities, and units for 1 kg of collected and sorted PA6 waste, based on the dataset for sorting of PA6-containing derelict fishing gear, as reported by Schneider et al. (2023) [5], with reference status of a PA6 waste fraction of 0.5. This activity serves as a SWITCH ACTIVITY input in the chemical recycling processes, where it is varied as an alternative scenario to other sorting options and serves as the base case. Activities subject to variation in the sensitivity analysis and Monte Carlo uncertainty analysis are highlighted in italics as SWITCH ACTIVITIES. 36
Table C 3: SWITCH ACTIVITY - Inventory table showing the activities, locations, quantities and units for 1kg of collected and sorted PA6 waste accorting to the dataset of mixed plastic waste sorting described by of Haupt et al. (2018) [6], with reference status of a PA6 waste fraction of 0.5. This activity itself serves as a SWITCH ACTIVITY input in the chemical recycling processes, where it is varied as an alternative scenario to the other sorting options. Activities subject to variation in the sensitivity analysis and Monte Carlo uncertainty analysis are highlighted in italics as SWITCH ACTIVITIES. 36
Table C 4: REFERENCE ACTIVITY - Inventory table showing the activities, locations, quantities and units for 1kg of collected, sorted, and purified PA6 by the dissolution process described by Costamagna et al. (2023) [7], with reference status of a PA6 waste fraction of 0.5 and a PA6 waste yield of 0.9. This activity itself serves as a SWITCH ACTIVITY input in the chemical recycling processes, where it is varied as an alternative scenario to the other purification options. Activities subject to variation in the sensitivity analysis and Monte Carlo uncertainty analysis are highlighted in italics as SWITCH ACTIVITIES. 37

Table C 5: SWITCH ACTIVITY - Inventory table listing activities, locations, quantities, and units for 1 kg of collected, sorted, and purified PA6 waste, based on the dataset for density separation and washing of PA6-containing derelict fishing gear, as reported by Schneider et al. (2023) [5], with reference status of a PA6 waste fraction of 0.5 and a PA6 waste yield of 0.9. This activity serves as a SWITCH ACTIVITY input in the chemical recycling processes, where it is varied as an alternative scenario to other purification options and serves as the base case. Activities subject to variation in the sensitivity analysis and Monte Carlo uncertainty analysis are highlighted in italics as SWITCH ACTIVITIES. 38
Table C 6: Inventory table showing the activities, locations, quantities and units for the production of 1kg CL by depolymerization of PA6 using H_3PO_4. The shown quantities are based on the reference calculation but altered within the sensitivity analysis according to changes of continuous parameters given in Section B.1. Activities subject to variation in the sensitivity analysis and Monte Carlo uncertainty analysis are highlighted in italics as SWITCH ACTIVITIES. 38
Table C 7: Inventory table showing the activities, locations, quantities and units for the production of 1kg CL by depolymerization of PA6 using HTW. The shown quantities are based on the reference calculation but altered within the sensitivity analysis according to changes of continuous parameters given in Section B.1. Activities subject to variation in the sensitivity analysis and Monte Carlo uncertainty analysis are highlighted in italics as SWITCH ACTIVITIES. 40
Table C 8: Inventory table showing the activities, locations, quantities and units for the production of 1kg CL by depolymerization of PA6 using iPrOH. The shown quantities are based on the reference calculation but altered within the sensitivity analysis according to changes of continuous parameters given in Section B.1. Activities subject to variation in the sensitivity analysis and Monte Carlo uncertainty analysis are highlighted in italics as SWITCH ACTIVITIES. 42
Table C 9: Inventory table showing the activities, locations, quantities and units for the production of 1kg CL by depolymerization of PA6 using NaOH. The shown quantities are based on the reference calculation but altered within the sensitivity analysis according to changes of continuous parameters given in Section B.1. Activities subject to variation in the sensitivity analysis and Monte Carlo uncertainty analysis are highlighted in italics as SWITCH ACTIVITIES. 43

Table D 1: Environmental impacts (reference case) for the four chemical recycling pathways for Nylon 6 to caprolactam, shown across all impact categories. 45
Table D 2: Environmental impacts (lower bound) for the four chemical recycling pathways for Nylon 6 to caprolactam, shown across all impact categories. 45
Table D 3: Environmental impacts (upper bound) for the four chemical recycling pathways for Nylon 6 to caprolactam, shown across all impact categories. 45
Table D 4: Midpoint LCIA results for the four PA6 to CL routes across EF 3.1, ReCiPe 2016 (H), and TRACI 2.1. For each EF 3.1 category, the most closely corresponding ReCiPe/TRACI midpoint category is reported. If no direct counterpart exists, the cell is left blank. Values are per functional unit under a harmonized system boundary while units may differ by method. 46
Table D 5: Annual GWP emissions of the four different chemical recycling pathways, calculated based on predicted PA6 production volumes, assuming that all produced PA6 will ultimately enter as waste for the chemical recycling. 67
Table D 6: Absolute Sustainability Ratios (ASR) for the four different chemical recycling pathways across various years. 67
Table D 7: GWP targets required to achieve an ASR of 1 across various years. 68
Table D 8: Overview of the calculation procedure, including the parameters and variables used, along with their meanings, units, values, and the corresponding references from which the data was sourced. 76

Table E 1: Explanations, arguments, and justifications for the methodological choices, including the LCA approach, scope and system boundaries. 69
Table E 2: mid-point indicators with abbreviations, units, descriptions, and robustness classes 71
Table E 3: Some impact categories that were excluded and reason for their exclusion 74
Table E 4: Mass yield of CL produced per unit of PA6 input for the four different chemical recycling pathways. 77

Figure Summary

Figure B 1: Schematic of the freshwater sink-float separation step for PA6 purification, as described in Schneider et al. (2023). 15
Figure B 2: schematic process flow diagram of the H_3PO_4 process. 17
Figure B 3: schematic process flow diagram of the HTW process. 20
Figure B 4: schematic process flow diagram of the iPrOH process. 23
Figure B 5: schematic process flow diagram of the NaOH process. 26

Figure D 1: Distributions of impact category results for the simulated process, showing mean (μ), median, standard deviation (σ), coefficient of variation (σ/μ), and 5th (p5) and 95th (p95) percentiles across climate change, ecotoxicity, eutrophication, human toxicity (carcinogenic and non-carcinogenic), ionizing radiation, land use, material resource depletion, and water use indicators for the depolymerization of PA6 using H_3PO_4 based on varying the following parameters outlined in section B.1: operating years, electricity source, PA6 waste fraction, PA6 sorting dataset, prepurification inclusion or exclusion, PA6 prepurification dataset, PA6 prepurification yield. 48
Figure D 2: Distributions of impact category results for the simulated process, showing mean (μ), median, standard deviation (σ), coefficient of variation (σ/μ), and 5th (p5) and 95th (p95) percentiles across climate change, ecotoxicity, eutrophication, human toxicity (carcinogenic and non-carcinogenic), ionizing radiation, land use, material resource depletion, and water use indicators for the depolymerization of PA6 using HTW based on varying the following parameters outlined in section C.4: operating years, electricity source, PA6 waste fraction, PA6 sorting dataset, prepurification inclusion or exclusion, PA6 prepurification dataset, PA6 prepurification yield, water amount, temperature, conversion, waste treatment dataset. 49
Figure D 3 Distributions of impact category results for the simulated process, showing mean (μ), median, standard deviation (σ), coefficient of variation (σ/μ), and 5th (p5) and 95th (p95) percentiles across climate change, ecotoxicity, eutrophication, human toxicity (carcinogenic and non-carcinogenic), ionizing radiation, land use, material resource depletion, and water use indicators for the depolymerization of PA6 using iPrOH based on varying the following parameters outlined in section B.1: operating years, electricity source, PA6 waste fraction, PA6 sorting dataset, prepurification inclusion or exclusion, PA6 prepurification dataset, PA6 prepurification yield, waste treatment dataset. 50
Figure D 4: Distributions of impact category results for the simulated process, showing mean (μ), median, standard deviation (σ), coefficient of variation (σ/μ), and 5th (p5) and 95th (p95) percentiles across climate change, ecotoxicity, eutrophication, human toxicity (carcinogenic and non-carcinogenic), ionizing radiation, land use, material resource depletion, and water use indicators for the depolymerization of PA6 using NaOH based on varying the following parameters outlined in section B.1: operating years, electricity source, PA6 waste fraction, PA6 sorting dataset, prepurification inclusion or exclusion, PA6 prepurification dataset, PA6 prepurification yield. 51
Figure D 5: Violin plot of impact category results for the simulated process, showing mean (μ), median, standard deviation (σ), coefficient of variation (σ/μ), and 5th (p5) and 95th (p95) percentiles across climate change, ecotoxicity, eutrophication, human toxicity (carcinogenic and non-carcinogenic), ionizing radiation, land use, material resource depletion, and water use indicators for the depolymerization of PA6 using H_3PO_4 based on varying the following parameters outlined in section B.1: operating years, electricity source, PA6 waste fraction, PA6 sorting dataset, prepurification inclusion or exclusion, PA6 prepurification dataset, PA6 prepurification yield. 52
Figure D 6: Violin plot of impact category results for the simulated process, showing mean (μ), median, standard deviation (σ), coefficient of variation (σ/μ), and 5th (p5) and 95th (p95) percentiles across climate change, ecotoxicity, eutrophication, human toxicity (carcinogenic and non-carcinogenic), ionizing radiation, land use, material resource depletion, and water use indicators for the depolymerization of PA6 using HTW based on varying the following parameters outlined in section C.4: operating years, electricity source, PA6 waste fraction, PA6 sorting dataset, prepurification inclusion or exclusion, PA6 prepurification dataset, PA6 prepurification yield, water amount, temperature, conversion, waste treatment dataset. 53
Figure D 7: Violin plot of impact category results for the simulated process, showing mean (μ), median, standard deviation (σ), coefficient of variation (σ/μ), and 5th (p5) and 95th (p95) percentiles across climate change, ecotoxicity, eutrophication, human toxicity (carcinogenic and non-carcinogenic), ionizing radiation, land use, material resource depletion, and water use indicators for the depolymerization of PA6 using iPrOH based on

varying the following parameters outlined in section B.1: operating years, electricity source, PA6 waste fraction, PA6 sorting dataset, prepurification inclusion or exclusion, PA6 prepurification dataset, PA6 prepurification yield, waste treatment dataset. 54
Figure D 8: Violin plot of impact category results for the simulated process, showing mean (μ), median, standard deviation (σ), coefficient of variation (σ/μ), and 5th (p5) and 95th (p95) percentiles across climate change, ecotoxicity, eutrophication, human toxicity (carcinogenic and non-carcinogenic), ionizing radiation, land use, material resource depletion, and water use indicators for the depolymerization of PA6 using NaOH based on varying the following parameters outlined in section B.1: operating years, electricity source, PA6 waste fraction, PA6 sorting dataset, prepurification inclusion or exclusion, PA6 prepurification dataset, PA6 prepurification yield. 55
Figure D 9: GWP as a function of process capacity factor (x_{CF}) for PA6 re-monomerization with two electricity supply scenarios: average German electricity mix and wind electricity. Data points represent GWP results from the parametric LCA model at varying capacity factors, assuming reference conditions: H_3PO_4 input of 0.266 kg/s, 15 years operating time, PA6 waste purification (dissolution), hazardous process waste treatment, PA6 waste fraction of 0.5, and PA6 waste purification yield of 99%. Solid lines correspond to the surrogate equations (shown in the plot boxes) derived for each scenario. Results illustrate the effect of electricity source and plant utilization on climate impacts. 57
Figure D 10: GWP as a function of process capacity factor (x_{CF}) for PA6 re-monomerization with two PA6 waste prepurification scenarios: with and without waste purification. Data points represent GWP results from the parametric LCA model at varying capacity factors, assuming reference conditions: H_3PO_4 input of 0.266 kg/s, 15 years operating time, German electricity mix, hazardous process waste treatment, PA6 waste fraction of 0.5, and PA6 waste purification yield of 99%. Solid lines correspond to the surrogate equations (displayed in the plot boxes) derived for each scenario. Results illustrate the effect of PA6 waste pre-purification and plant utilization on climate impacts. 57
Figure D 11: GWP as a function of the phosphoric acid mass flow rate for PA6 re-monomerization, comparing two process waste treatment scenarios: hazardous waste treatment and non-hazardous waste treatment. Data points represent GWP results from the parametric LCA model at varying H_3PO_4 mass flow rates, assuming 15 years operating time, German electricity mix, full PA6 waste prepurification (dissolution), PA6 waste fraction of 0.5, and PA6 waste purification yield of 99%. Solid lines correspond to the surrogate equations (displayed in the plot boxes) derived for each scenario. The results demonstrate the influence of phosphoric acid usage and waste treatment strategy on the overall climate impact of the process. 58
Figure D 12: Freshwater ecotoxicity (FET) as a function of the phosphoric acid mass flow rate for PA6 re-monomerization, comparing hazardous and non-hazardous waste treatment scenarios. Data points show FET results from the parametric LCA model at varying H_3PO_4 mass flow rates, assuming 15 years operating time, German electricity mix, full PA6 waste prepurification (dissolution), PA6 waste fraction of 0.5, and PA6 waste purification yield of 99%. Solid lines represent the corresponding surrogate equations (displayed in the plot boxes) for each scenario. The results illustrate the substantial influence of both phosphoric acid usage and waste treatment strategy on freshwater ecotoxicity impacts. 58
Figure D 13: GWP as a function of process capacity factor (x_{CF}) for PA6 re-monomerization with two electricity supply scenarios: average German electricity mix and wind electricity. Data points represent GWP results from the parametric LCA model at varying capacity factors, assuming reference conditions: HTW input of 14.95 kg/s, conversion of 0.89, reactor temperature of 345 °C, 15 years operating time, PA6 waste purification (dissolution), PA6 waste fraction of 0.5, and PA6 waste purification yield of 99%. Solid lines correspond to the surrogate equations (shown in the plot boxes) derived for each scenario. Results illustrate the effect of electricity source and plant utilization on climate impacts. 60
Figure D 14: GWP as a function of operating years for PA6 re-monomerization with two electricity supply scenarios: average German electricity mix and wind electricity. Data points represent GWP results from the parametric LCA model at varying capacity factors, assuming reference conditions: HTW input of 14.95 kg/s, conversion of 0.89, reactor temperature of 345 °C, a capacity factor of 0.9, PA6 waste purification (dissolution), PA6 waste fraction of 0.5, and PA6 waste purification yield of 99%. Solid lines correspond to the surrogate equations (displayed in the plot boxes) derived for each scenario. Results illustrate the effect of PA6 waste pre-purification and operating years on climate impacts. 60
Figure D 15: GWP as a function of water mass flow rate for PA6 re-monomerization, comparing two scenarios: with and without PA6 waste purification. Data points represent GWP results from the parametric LCA model at varying water flow rates, assuming reference conditions: HTW input of 14.95 kg/s, conversion of 0.89, reactor temperature of 345 °C, 15 years operating time, a capacity factor of 0.9, German electricity mix, PA6 waste fraction

of 0.5, and PA6 waste purification yield of 99%. Solid lines correspond to the surrogate equations (displayed in the plot boxes) derived for each scenario. The results demonstrate the influence of water usage and waste purification strategy on the climate impact of the process. 61
Figure D 16: GWP as a function of PA6 conversion for PA6 re-monomerization, comparing scenarios with and without PA6 waste purification. Data points represent GWP results from the parametric LCA model at varying conversion rates, assuming reference conditions: HTW input of 14.95 kg/s, reactor temperature of 345 °C, a capacity factor of 0.9, 15 years operating time, German electricity mix, PA6 waste fraction of 0.5, and PA6 waste purification yield of 99%. Solid lines correspond to the surrogate equations (displayed in the plot boxes) derived for each scenario. The results demonstrate the effect of process conversion and waste purification on the climate impact of the process.. 61
Figure D 17: GWP as a function of process capacity factor (x_{CF}) for PA6 re-monomerization with two electricity supply scenarios: average German electricity mix and wind electricity. Data points represent GWP results from the parametric LCA model at varying capacity factors, assuming the reference conditions iPrOH input of 6.69 kg/s, 15 years operating time, PA6 waste purification (dissolution), PA6 waste fraction of 0.5, and PA6 waste purification yield of 99%. Solid lines correspond to the surrogate equations (shown in the plot boxes) derived for each scenario. Results illustrate the effect of electricity source and plant utilization on climate impacts. 63
Figure D 18: GWP as a function of operating years for PA6 re-monomerization with two electricity supply scenarios: average German electricity mix and wind electricity. Data points represent GWP results from the parametric LCA model at varying number of operating years, assuming the reference conditions iPrOH input of 6.69 kg/s, 15 years operating time, PA6 waste purification (dissolution), PA6 waste fraction of 0.5, and PA6 waste purification yield of 99%. Solid lines correspond to the surrogate equations (displayed in the plot boxes) derived for each scenario. Results illustrate the effect of electricity source and operating time on climate impacts......... 63
Figure D 19: GWP as a function of process capacity factor (x_{CF}) for PA6 re-monomerization with two electricity supply scenarios: average German electricity mix and wind electricity. Data points represent GWP results from the parametric LCA model at varying capacity factors, assuming the reference conditions: 15 years operating time, PA6 waste purification (dissolution), PA6 waste fraction of 0.5, PA6 waste purification yield of 99% and CL recovery of 99.7%. Solid lines correspond to the surrogate equations (shown in the plot boxes) derived for each scenario. Results illustrate the effect of electricity source and plant utilization on climate impacts.................... 65
Figure D 20: GWP as a function of operating years for PA6 re-monomerization with two electricity supply scenarios: average German electricity mix and wind electricity. Data points represent GWP results from the parametric LCA model at varying number of operating years, assuming the reference iPrOH input, a capacity factor of 0.9, PA6 waste purification (dissolution), PA6 waste fraction of 0.5, PA6 waste purification yield of 99% and CL recovery of 99.7%. Solid lines correspond to the surrogate equations (displayed in the plot boxes) derived for each scenario. Results illustrate the effect of electricity source and operating time on climate impacts......... 65
Figure D 21: GWP as a function of CL recovery for PA6 re-monomerization comparing two scenarios: with and without PA6 waste purification. Data points represent GWP results from the parametric LCA model at CL recovery, assuming reference conditions: capacity factor of 0.9, 15 years operating time, PA6 waste purification (dissolution), PA6 waste fraction of 0.5, PA6 waste purification yield of 99% and CL recovery of 99.7%. Solid lines correspond to the surrogate equations (displayed in the plot boxes) derived for each scenario. The results demonstrate the influence of CL recovery and waste purification strategy on the climate impact of the process. 66

A Property & Thermodynamic Data Collection

A.1 Scalar Properties

Enthalpy of Formation

In process simulation using Aspen Plus, specifying accurate thermodynamic properties such as the enthalpy of formation is crucial for reliable modeling outcomes and accurate energy balances. Since the reaction enthalpy and the monomer's enthalpy of formation are available, the enthalpy of formation for the resulting polymer was derived using theoretical calculations due to the scarcity of direct experimental data. Therefore, the reaction enthalpy of caprolactam polymerization, ΔH_{poly} , multiplied by the degree of polymerization n gives the total reaction enthalpy $\Delta H_{\text{poly total}} \frac{\text{J}}{\text{mol PA6}} = \Delta H_{\text{poly}} \times n$. Concurrently, the cumulative standard enthalpy of formation for all the involved monomers, was computed as $\Delta H_f^\circ(\text{CL}) \times n$. These values were then summed to establish the overall enthalpy of formation for PA6 $\Delta H_f^\circ(\text{PA6}) = \Delta H_{f\ CL\ total}^\circ + \Delta H_{\text{poly total}}$.

Enthalpy of Melting

PA6 is set in the following way: Solid heat of formation in Aspen Plus for PA6 is changed such that the melting enthalpy matches exactly 188 J/g [1].

Table A 1: Scalar properties of PA6 taken from the Polymer Handbook [2].

**Parameters**	**Units**	**PA6**
Standard enthalpy of formation (25C)	kJ/kmol	-65642100
Solid enthalpy of formation (25C)	kJ/kmol	-69776300
Melting temperature	C	220
Molecular weight	g/mole	21953.04
Glass transition temperature	C	59
Degree of polymerization		194
Polydispersity index		2
Melting enthalpy	J/g	188

A.2 Temperature-Dependent Properties

A.2.1 Experimental Data

Table A 2: Liquid and solid heat capacity data of PA6 sourced from [2,3].

**T [K]**	**cp [J/g/K]**	**T [K]**	**cp [J/g/K]**	**T [K]**	**cp [J/g/K]**
70	0.44	270	1.35	430	2.56
80	0.50	273	1.36	440	2.57

90	0.55	280	1.39	450	2.59
100	0.60	290	1.45	460	2.60
110	0.65	298	1.49	470	2.61
120	0.69	300	1.50	480	2.63
130	0.74	310	1.56	490	2.64
140	0.78	313	1.57	500	2.65
150	0.82	313	2.40	510	2.67
160	0.86	320	2.41	520	2.68
170	0.90	330	2.42	530	2.69
180	0.94	340	2.44	540	2.71
190	0.98	350	2.45	550	2.72
200	1.03	360	2.46	560	2.73
210	1.07	370	2.48	570	2.75
220	1.11	380	2.49	580	2.76
230	1.16	390	2.51	590	2.77
240	1.20	400	2.52	600	2.79
250	1.25	410	2.53		
260	1.30	420	2.55		

A.2.2 Polynomials and Parameters

Aspen ideal gas heat capacity polynomial

$$C_p^{*,ig} = C_{1i} + C_{2i}T + C_{3i}T^2 + C_{4i}T^3 + C_{5i}T^4 + C_{6i}T^5 \text{ for } C_{7i} \leq T \leq C_{8i}$$
$$C_p^{*,ig} = C_{9i} + C_{10i}T^{C_{11i}} \text{ for } T < C_{7i}$$

Table A 3: parameters for the polynomial of ideal gas heat capacity

Unit	1	2	3	4	5	6	7	8	9	10
J/kJmol/K	42988240	32535	-6.187	0.00428	0	0	0	2000	0	0

DIPPR equation for liquid heat capacity

$$C_{p,i}^{*,l} = C_{1i} + C_{2i}T + C_{3i}T^2 + C_{4i}T^3 + C_{5i}T^4 \text{ for } C_{6i} \leq T \leq C_{7i}$$

Table A 4: parameters for the DIPPR equation for liquid heat capacity

Unit	1	2	3	4	5	6	7
J/kJmol/K	477121.356	328.980302	477121.356	328.980302	0	313	600

DIPPR equation for solid heat capacity

$$C_{p,i}^{*,s} = C_{1i} + C_{2i}T + C_{3i}T^2 + C_{4i}T^3 + C_{5i}T^4 \text{ for } C_{6i} \leq T \leq C_{7i}$$

Table A 5: parameters for the DIPPR equation for solid heat capacity

Unit	1	2	3	4	5	6	7
J/kJmol/K	1000	123.008312	0.094201	-0.0015295	3.448E-06	0	1000

A.3 Binary Interaction Parameters

NRTL method is used as it is recommended for highly non-ideal chemical systems and is suitable for both VLE and LLE applications.

$$ln(\gamma_i) = \frac{\sum_j \tau_{ji} G_{ji} x_j}{\sum_k G_{ki} x_k} + \sum_j \frac{G_{ij} x_j}{\sum_k G_{kj} x_k}\left(\tau_{ij} - \frac{\sum_m \tau_{mj} G_{mj} x_m}{\sum_k G_{kj} x_k}\right)$$

Where:

$$G_{ij} = exp(-\alpha_{ij} \tau_{ij})$$
$$\tau_{ij} = a_{ij} + \frac{b_{ij}}{T} + e_{ij} ln(T) + f_{ij}T$$
$$\alpha_{ij} = c_{ij} + d_{ij} (T - 273.15 K)$$
$$\tau_{ii} = 0$$
$$G_{ii} = 1$$

The Aspen Physical Property System includes numerous built-in binary parameters for the NRTL model, which are also utilized in its variants for polymers and electrolytes. These binary parameters were regressed using VLE and LLE data sourced from the Dortmund Databank. For VLE applications, the regression was conducted employing the ideal gas law, Redlich-Kwong, and Hayden-O'Connell equations of state. If the built-in binary parameters were not available, UNIFAC was used for their estimation.

Table A 6: NRTL parameters for the components and their source. The parameters D_{IJ}, E_{IJ}, E_{JI}, F_{IJ}, and F_{JI} are 0 and the unit for temperature is in Kelvin.

"Component i"	"Component j"	"Source"	"A_{IJ}"	"A_{JI}"	"B_{IJ}"	"B_{JI}"	"C_{IJ}"
CL	iPrOH	NISTV120 NIST-IG	3.445	1.534	240.863	-1091.190	0.1
CL	Esterified ACA	UNIFAC	0	0	725.470	-93.150	0.3
CL	ACA	UNIFAC	0	0	504.838	-57.034	0.3
CL	Water	APV120 VLE-RK	-0.798	-4.018	1965.523	907.909	0.3
CL	ACA dimer	UNIFAC	0	0	1000.159	-21.676	0.3
iPrOH	ACA	UNIFAC	0	0	-806.008	1644.471	0.3
iPrOH	Esterified ACA	UNIFAC	0	0	-605.333	997.654	0.3
Water	ACA dimer	UNIFAC	0	0	1657.122	-478.008	0.3
Water	ACA	UNIFAC	0	0	1137.342	-424.160	0.3
H_3PO_4	Water	NISTV120 NIST-HOC	-5.856	12.829	1486.610	-5183.080	0.334

B Process and Parameter Descriptions

B.1 Varied Parameters for Sensitivity Analysis, Distributions and Uncertainty Ranges

This section lists the parameters and scenario switches that were varied to test robustness of the results and to compute the uncertainty ranges shown for each impact category. Continuous parameters were varied within the ranges reported in the tables. Discrete parameters were implemented as alternative background datasets.

We distinguish two uses of parameter variation. We performed a one-at-a-time sensitivity analyses to quantify how strongly individual modelling choices and uncertain inputs affect the impact results. In these runs, a single parameter (or discrete scenario switch) is changed while all other inputs are kept at their base-case reference setting. Second, we compute uncertainty ranges (error bars) for the base case by propagating a subset of uncertainties that reflect missing information or inherent variability in the supply chains and pathways. For this uncertainty propagation, the explored bounds are interpreted as plausible extreme values, and probability distributions are generated by Monte Carlo simulation. Parameters that were only explored for testing their influence remain sensitivity-only and are not included in the uncertainty bounds.

Table B 1: Overview of the parameters, including their type, reference setting, and description of each parameter.

Name of parameter	Type	Reference setting	Description	Uncertainty bounds
Years	Continuous	15 years	Varies the total plant operation years between 10 and 20 years.	x
Electricity source	Discrete	German electricity grid	Switches between 1) German electricity grid and 2) wind-based electricity available as datasets in Ecoinvent v3.9.1 [4]	x
PA6 waste fraction	Continuous	0.5	Varies the mass fraction of PA6 in the incoming waste stream between 0.3 and 0.9.	x
Collection and sorting system	Discrete	Sorting of fishing gear containing PA6 [5]	Switches between waste collection and sorting datasets of 1) fishing gear containing PA6 including shredding [5] 2) mixed plastic waste [6] 3) PE waste [4], and 4) PET waste [4].	x
PA6 pre-purification status	Discrete	Included	1) Includes or 2) excludes the impacts of PA6 dissolution, as a pre-purification step of PA6 waste.	x
PA6 pre-purification method	Discrete	Dissolution [7]	Switches between 1) dissolution of PA6 from packaging waste [7] and 2) density separation and washing of fishing gear containing PA6 [5].	x
PA6 purification yield	Continuous	0.9	Varies the kg of PA6 produced per kg input waste (yield) between 0.85 and 0.99.	x
HTW process: Reactor temperature, pressure HTW:PA6 ratio, CL reaction yield	Continuous	345 °C, 16 MPa, HTW:PA6 ratio = 30, CL reaction yield = 89%	Varies the temperature, conversion, PA6 to HTW ratio and CL reaction yield between 300 °C, 10 MPa, 11, and 91.6% [8], to 345 °C, 16 MPa, 30 and 89% [9]	x
H_3PO_4 process: H_3PO_4:PA6 ratio	Continuous	0.18	Varies H_3PO_4:PA6 ratio between 0.18 to 0.49	

iPrOH process: iPrOH:PA6 ratio	Continuous	12.8	Varies iPrOH:PA6 ratio between 4.7 and 12.8	
NaOH process: CL recovery	Continuous	99.967%	Varies the CL recovery between 96.6% and 99.9%	x
Process waste treatment option	Discrete	Incineration of PET waste	Switches between incineration of 1) PET, 2) PE, and 3) mixed plastic waste [4] (HTW and iPrOH processes).	

For the probability distributions of impact categories and calculation of uncertainty ranges, the HTW process was varied in reaction temperature, conversion, and reaction agent amount due to inconsistent and highly variable data reported in the literature for this pathway. As can be seen in Table B 2, the optimum solvent-to-feed ratios range from 11:1 to 30:1, with operating temperatures between 300°C and 345°C, achieving CL yields of 85% to 92.5%. As reference, the conditions reported by Wang et al. (2014) [9] were taken.

Table B 2: Varying investigated reaction temperatures, pressures and times with different optimum conditions reported by the literature for the HTW process

**Literature**	**Conditions**	**Solvent / Catalyst**	**Main Products**	**Best S/F**	**T / p**	**Time (min)**	**K**	**Y [%]**
				Neutral Hydrolysis				
Iwaya et al. (2006) [10]	300-400 °C, 20-35MPa, 5-60 min	HTW	CL, ACA	29.5:1	300 °C	60	x	85
Wang et al. (2014) [9]	270-370 °C, 3-16 MPa, 15-75 min	HTW	CL, ACA, CL dimer, trimer	30 :1	345 °C, 16 MPa	75	-	89
Darzi et al. (2022) [11,12]	250-400 °C, 30-90 min	HTW	CL, CL dimer, cyclopentene	30	300 °C, 10MPa	90	-	92.5
Hu et al. (2022)	280-310 °C, 20-80 min	HTW	CL, CL&ACA dimer, oligomers	11:1	300 °C -	60	-	91.6

For the other pathways, these parameters were not included in the uncertainty ranges or probability distributions but were varied in the sensitivity analysis, following the same approach as for the HTW process, to assess the potential influence of such variations.

B.2 Background Systems

The background systems represent upstream and downstream processes that are not part of the core chemical recycling steps, but are essential to the overall life cycle inventory. These systems, including the sorting and purification of PA6 waste, as well as the incineration of process residues, were not modelled actively but taken based on literature data to ensure a comprehensive and comparable assessment across all scenarios. Several alternative datasets were assumed for each background process to ensure the model remains broadly applicable to different types of plastic waste. These datasets were implemented as variable activities (switch activities) in the Python package lca_algebraic and systematically varied in the statistical analysis to capture uncertainty and generate error bars for each impact category.

B.2.1 Assumed Sorting Systems

To ensure representativeness and comparability across different PA6 recycling scenarios, four alternative sorting datasets were considered: the Schneider et al. (2023) process for fishing gear [5], the dataset from Haupt et al. (2018) for mixed plastic waste [6], and two ecoinvent datasets for the sorting of waste polyethylene and waste PET [4]. These scenarios reflect relevant real-world collection streams, enabling robust sensitivity analysis in the LCA.

B.2.1.1 Reference - Sorting of Fishing Gear containing PA6

The primary dataset for sorting is derived from Schneider et al. (2023) [5], who performed a comparative attributional LCA assessing different waste management pathways for derelict fishing gear, including landfill disposal, syngas

production, energy recovery, and mechanical recycling. Their process chain explicitly models the sequential sorting steps required for fishing gear containing Nylon 6. During sorting, large metal components such as anchors, chains, and rocks are removed, with a steel separation efficiency of 79.2% based on experimental data. Subsequently, the waste undergoes coarse and fine shredding, with integrated magnetic separation assumed to remove remaining metal scrap at 100% efficiency. Following the cut-off approach, separated steel is considered a recyclable material, leaving the system without allocation of further environmental burdens or credits. The retrieval of the fishing gear was not included.

B.2.1.2 Proxy - Sorting of Mixed Plastic Waste

The sorting of mixed plastic waste was modeled based on the life cycle inventory provided by Haupt et al. (2018) [6]. Sorting includes sorting out residues and the use of Near Infrared Radiation (NIR) technology to separate different polymers.

B.2.1.3 Proxy - Sorting of PE and PET

For scenarios based on packaging waste streams dominated by single polymer types, the sorting of waste PE and PET was modeled using the ecoinvent v3.9.1 (cut-off) inventories "treatment of waste polyethylene, for recycling, unsorted, sorting" and "treatment of waste polyethylene terephthalate, for recycling, unsorted, sorting." These datasets represent the sorting of collected, unsorted PE and PET waste into fractions suitable for mechanical recycling. The modeled process reflects typical material recovery facility operations, where incoming mixed plastic waste is sorted through a combination of mechanical separation, screening, and polymer identification technologies such as NIR sorting [13]. Non-target materials and contaminants are separated for disposal or energy recovery, while sorted PE or PET fractions are prepared for downstream recycling processes. The inventories include energy use, auxiliary materials, and emissions associated with the sorting process, based on average European data. The functional unit for both inventories is 1 kg of collected, unsorted waste PE or PET. The datasets are widely used as proxies for polymer-specific sorting processes in LCA studies, and provide a practical benchmark for scenarios where detailed, site-specific sorting data are unavailable [6].

B.2.2 Assumed Pre-Purification Systems

If the initial sorting process does not yield a Nylon 6 fraction with sufficient purity for chemical recycling, such as when additional plastics are present, further purification steps are required to obtain a suitable feedstock. The necessity for such pre-purification also depends on the robustness of the specific chemical recycling process. For a conservative baseline, it was assumed that pre-purification is included in the inventory; however, this parameter is varied in the sensitivity analysis and error bar calculations by switching the pre-purification option on or off. Varying both the inclusion and method of pre-purification ensures that the calculations are not limited to a single waste type, but remain broadly applicable to a range of real-world waste streams. As a result, the model may yield broader error bars, but this ultimately strengthens the robustness and generalizability of the assessment. Two alternative PA6 purification datasets were considered: a two-stage density separation based on derelict fishing gear (Schneider et al. (2023) [5]), and a dissolution-based process for mixed PE/PA6 waste Costamagna et al. (2023) [7]).

B.2.2.1 Density Separation and Washing of Fishing Gear containing PA6

The purification dataset, based on Schneider et al. (2023) models, involves a two-stage density separation with washing and drying . In the first stage of density separation, heavy contaminants such as lead weights and mineral sediments are removed using saline water (~1.15 g/cm³), achieving a separation efficiency of 90% for minerals and 100% for lead. The second stage employs freshwater to separate lighter polymers such as PP and PE, which float, from the denser PA6, which sinks. Subsequently, the PA6-rich fraction is washed to remove residual minerals and organic matter, with a 15% polymer mass loss assumed and complete mineral removal. Finally, the cleaned PA6 fibres are dried. Based on these steps, the resulting material is assumed, in alignment with Schneider et al. (2023), to be of sufficient purity to substitute average virgin Nylon 6 in the life cycle assessment, supporting its suitability as a feedstock for chemical recycling.

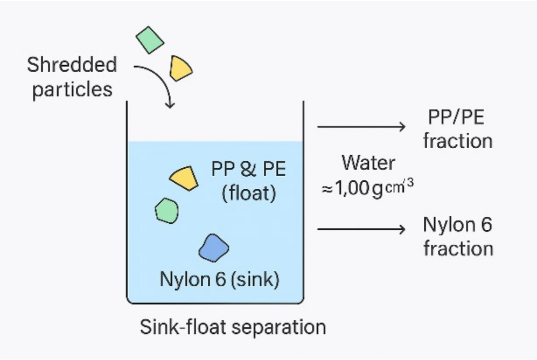

Figure B 1: Schematic of the freshwater sink-float separation step for PA6 purification, as described in Schneider et al. (2023).

B.2.2.2 Reference - Dissolution of PA6/PE Packaging Waste

The purification dataset based on Costamagna et al. (2023) models the selective dissolution of PA6 from mixed polyamide/polyethylene (PA/PE) packaging waste [7]. Shredded multilayer PA/PE films are treated with monoethylene glycol (MEG) at elevated temperature (~160 °C), selectively dissolving the PA6 fraction while PE remains undissolved. The undissolved PE is filtered and washed with hot MEG and water, while the dissolved PA6 is recovered by cooling and precipitation, followed by filtration and washing to remove residual solvent. Both polymers are subsequently dried. The process incorporates solvent and water recovery via distillation, with continuous recycling during operation and makeup to compensate for losses. Based on experimental results, the process achieves efficient separation of PA6 and PE, with the input stream typically consisting of 30% PA6 and 70% PE by mass, and both fractions recovered at nearly 100% yield. The recovered PA6 is assumed to be of sufficient purity to substitute for virgin PA6 in the life cycle assessment, supporting its suitability as a feedstock for chemical recycling.

B.2.3 Proxy - Incineration of Process Waste

All processes generate a waste stream containing unconverted PA6, side products, and oligomers, with H_3PO_4 and NaOH present in the acidic and basic processes, respectively. These streams are modeled using the hazardous waste treatment dataset from the ecoinvent database for the acidic and alkaline processes [4]. For the alcoholysis and HTW depolymerization processes, no PA6-specific waste treatment dataset is available, so proxies for ecoinvent v3.3 datasets “treatment of waste polyethylene terephthalate, municipal incineration”, “treatment of waste polyethylene, municipal incineration” and “treatment of waste plastic, mixture, municipal incineration” were used. These four incineration systems employ wet flue gas scrubbing along with selective catalytic reduction for the removal of nitrogen oxides, ensuring modern and efficient treatment practices in accordance with European standards [14]. All three proxies for the HTW and iPrOH processes are included in the sensitivity analysis to assess their influence on the overall results. Following the cut-off approach, no credits are allocated for recovered energy; only the direct environmental burdens from incineration are assigned to the process waste. This proxy modeling provides a consistent and conservative basis for assessing the end-of-life treatment of chemical recycling residues in the absence of dedicated PA6 incineration data.

B.3 Foreground System – Chemical Recycling Processes

Detailed descriptions of the foreground chemical recycling processes are provided in the main text of the paper. The following sections give explanations regarding the TRL and the process flow diagrams and stream summaries.

B.3.1 Technology Readiness Level

Acid-catalyzed (H_3PO_4 + steam): Industrial practice for in-plant scrap via steam in the presence of phosphoric acid has been described historically [15]. However, publicly available sources do not provide sufficient detail on mass/energy balances or catalyst management for mixed post-consumer feeds. For the specific configuration assessed here, we classify the maturity as TRL 4-5, based on evidence limited to patent literature and reports of continuous operation, without publicly available plant-scale validation for mixed feeds.

Hydrothermal water (HTW): Bench studies report high yields for PA6 depolymerization in hot compressed (pressurized) water under subcritical to near-critical conditions [8]. In addition, patent literature describes an integrated process concept for multi-component PA6-containing waste with pressurized-water depolymerization and downstream solid-liquid separation and caprolactam recovery, including a continuous stirred-tank reactor [16]. However, publicly available evidence remains limited to lab scale and patent disclosures, without open pilot/plant mass-energy balances or long-run operability data for mixed post-consumer feeds. We therefore assign TRL 4-5.

Alkaline, solvent-free with direct CL removal (vacuum distillation): Patent literature describes molten-phase, base-catalyzed PA6 depolymerization at 250–320 °C where CL is withdrawn directly as vapor under reduced pressure and condensed [17]. Recent bench studies demonstrate solvent-free NaOH/KOH depolymerization of virgin and dyed PA6 wastes to high-purity CL (in some cases repolymerized to PA6 or claimed without extra distillation) [18]. Hence, we classify TRL 4-5 for this configuration.

Alcoholysis (iPrOH / secondary alcohols): Patent literature demonstrates catalyst-free depolymerization of PA6 in supercritical secondary alcohols, with CL yields quantified in small sealed-tube experiments. Downstream solvent removal and monomer purification are described conceptually and continuous operation is suggested, but no public continuous embodiment, integrated mass/energy balances, or mixed post-consumer feeds are disclosed [19]. We therefore assign TRL 3-4 for this configuration.

B.3.2 Process Flow Schemes, Stream Summaries and Equipment Summaries

B.3.2.1 H_3PO_4 Process

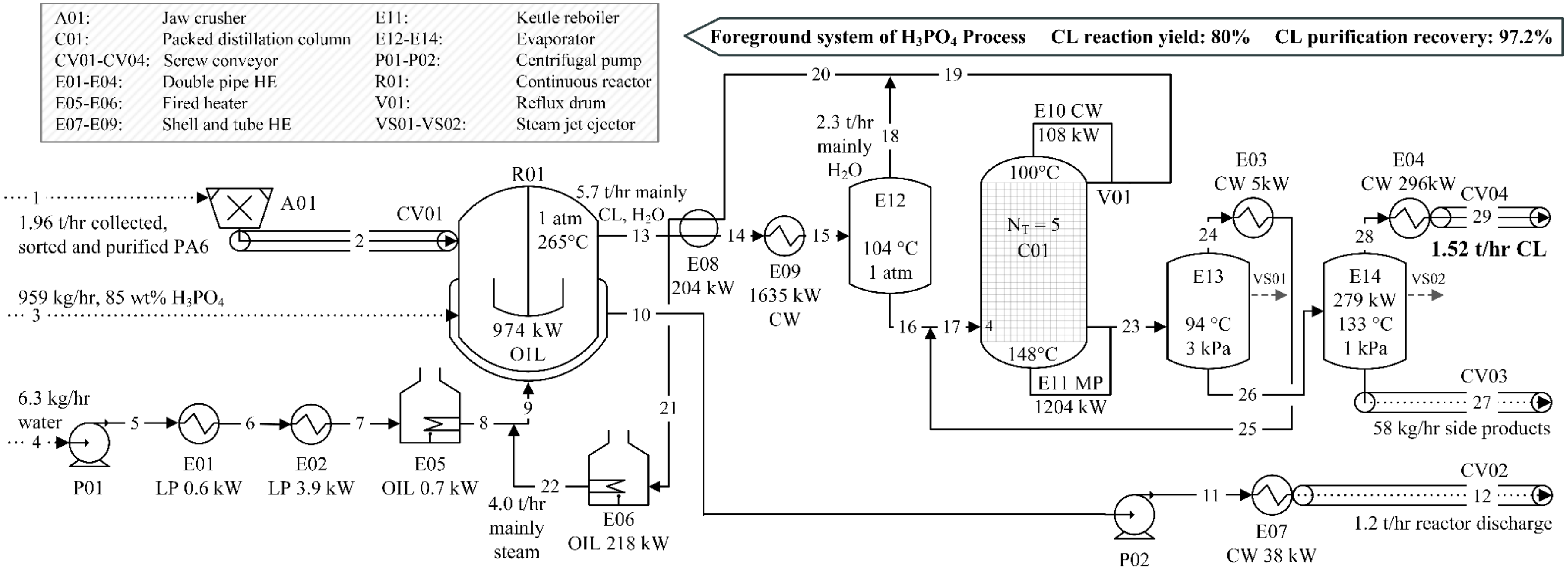

Figure B 2: schematic process flow diagram of the H_3PO_4 process.

Table B 3: stream table of the H_3PO_4 process including information on phases, temperatures, pressures, mass flows, mass fractions and enthalpy flows of the streams.

Stream Name	Units	1	2	3	4	5	6	7	8	9	10
Phase	-	Solid	Solid	Liquid	Liquid	Liquid	Liquid	Vapor	Vapor	Vapor	Liquid
Temperature	°C	25.00	25.00	25.00	25.00	25.06	108.76	106.54	300.00	300.00	265.00
Pressure	bar	1.01	1.01	1.01	1.01	1.72	1.37	1.27	1.02	1.01	1.01

Mass Flows	TOTAL	kg/hr	1957.44	1957.44	959.42	6.29	6.29	6.29	6.29	6.29	3962.80	1211.80
	CL	kg/hr	0.00	0.00	0.00	0.00	0.00	0.00	0.00	0.00	3.13	141.53
	WATER	kg/hr	0.00	0.00	143.91	6.29	6.29	6.29	6.29	6.29	3959.66	30.20
	PA6	kg/hr	1957.44	1957.44	0.00	0.00	0.00	0.00	0.00	0.00	0.00	224.56
	H_3PO_4	kg/hr	0.00	0.00	815.51	0.00	0.00	0.00	0.00	0.00	0.00	815.51
	ACADIMER	kg/hr	0.00	0.00	0.00	0.00	0.00	0.00	0.00	0.00	0.00	0.00
Mass Fractions	CL	-	0.00	0.00	0.00	0.00	0.00	0.00	0.00	0.00	0.00	0.12
	WATER	-	0.00	0.00	0.15	1.00	1.00	1.00	1.00	1.00	1.00	0.02
	PA6	-	1.00	1.00	0.00	0.00	0.00	0.00	0.00	0.00	0.00	0.19
	H_3PO_4	-	0.00	0.00	0.85	0.00	0.00	0.00	0.00	0.00	0.00	0.67
	ACADIMER	-	0.00	0.00	0.00	0.00	0.00	0.00	0.00	0.00	0.00	0.00
Enthalpy Flow		kW	-1728.22	-1728.22	-3656.73	-27.70	-27.70	-27.06	-23.17	-22.51	-14183.70	-3147.01

**Stream Name**		**Units**	**11**	**12**	**13**	**14**	**15**	**16**	**17**	**18**	**19**	**20**
Phase		-	Liquid	Liquid	Mixed	Mixed	Mixed	Liquid	Liquid	Vapor	Vapor	Mixed
Temperature		°C	265.04	40.00	264.88	199.22	104.00	104.00	103.78	104.00	100.02	102.31
Pressure		bar	1.32	1.01	1.01	1.01	1.01	1.01	1.01	1.01	1.01	1.01
Mass Flows	TOTAL	kg/hr	1211.80	1211.80	5667.85	5667.85	5667.85	3393.69	3417.72	2274.16	1682.33	3956.49
	CL	kg/hr	141.53	141.53	1565.95	1565.95	1565.95	1562.82	1586.82	3.13	0.00	3.13
	WATER	kg/hr	30.20	30.20	4073.37	4073.37	4073.37	1802.35	1802.37	2271.03	1682.33	3953.35
	PA6	kg/hr	224.56	224.56	0.00	0.00	0.00	0.00	0.00	0.00	0.00	0.00
	H_3PO_4	kg/hr	815.51	815.51	0.00	0.00	0.00	0.00	0.00	0.00	0.00	0.00
	ACADIMER	kg/hr	0.00	0.00	28.53	28.53	28.53	28.53	28.53	0.00	0.00	0.00
Mass Fractions	CL	-	0.12	0.12	0.28	0.28	0.28	0.46	0.46	0.00	0.00	0.00
	WATER	-	0.02	0.02	0.72	0.72	0.72	0.53	0.53	1.00	1.00	1.00

	PA6	-	0.19	0.19	0.00	0.00	0.00	0.00	0.00	0.00	0.00	0.00
	H_3PO_4	-	0.67	0.67	0.00	0.00	0.00	0.00	0.00	0.00	0.00	0.00
	ACADIMER	-	0.00	0.00	0.01	0.01	0.01	0.01	0.01	0.00	0.00	0.00
Enthalpy Flow		kW	-3146.97	-3415.34	-15449.49	-15653.29	-17288.32	-8912.30	-8930.29	-8376.02	-6206.97	-14582.99

**Stream Name**		**Units**	**21**	**22**	**23**	**24**	**25**	**26**	**27**	**28**	**29**	**Volatiles of VS01**	**Volatiles of VS02**
Phase		-	Vapor	Vapor	Liquid	Vapor	Mixed	Liquid	Liquid	Vapor	Liquid	Vapor	Vapor
Temperature		°C	199.23	300.00	147.58	93.77	74.61	93.77	40.00	133.21	40.00	74.61	69.70
Pressure		bar	1.01	0.76	1.01	0.03	0.03	0.03	1.01	0.01	1.01	0.03	0.01
Mass Flows	TOTAL	kg/hr	3956.49	3956.49	1735.39	152.60	24.03	1582.80	57.87	1524.93	1522.17	137.35	11.54
	CL	kg/hr	3.13	3.13	1586.82	34.17	24.00	1552.65	29.34	1523.32	1522.07	10.16	1.25
	WATER	kg/hr	3953.35	3953.35	120.04	118.43	0.02	1.62	0.00	1.62	0.10	118.40	1.52
	PA6	kg/hr	0.00	0.00	0.00	0.00	0.00	0.00	0.00	0.00	0.00	0.00	0.00
	H_3PO_4	kg/hr	0.00	0.00	0.00	0.00	0.00	0.00	0.00	0.00	0.00	0.00	0.00
	ACADIMER	kg/hr	0.00	0.00	28.53	0.00	0.00	28.53	28.53	0.00	0.00	0.00	0.00
Mass Fractions	CL	-	0.00	0.00	0.91	0.22	1.00	0.98	0.51	1.00	1.00	0.07	0.11
	WATER	-	1.00	1.00	0.07	0.78	0.00	0.00	0.00	0.00	0.00	0.86	0.13
	PA6	-	0.00	0.00	0.00	0.00	0.00	0.00	0.00	0.00	0.00	0.00	0.00
	H_3PO_4	-	0.00	0.00	0.00	0.00	0.00	0.00	0.00	0.00	0.00	0.00	0.00
	ACADIMER	-	0.00	0.00	0.02	0.00	0.00	0.02	0.49	0.00	0.00	0.00	0.00
			-14379.18	-14161.09	-1628.53	-456.82	-17.99	-1171.71	-47.31	-848.95	-1161.12	-444.19	-6.24
Enthalpy Flow		kW	199.23	300.00	147.58	93.77	74.61	93.77	40.00	133.21	40.00	74.61	69.70

B.3.2.2 HTW Process

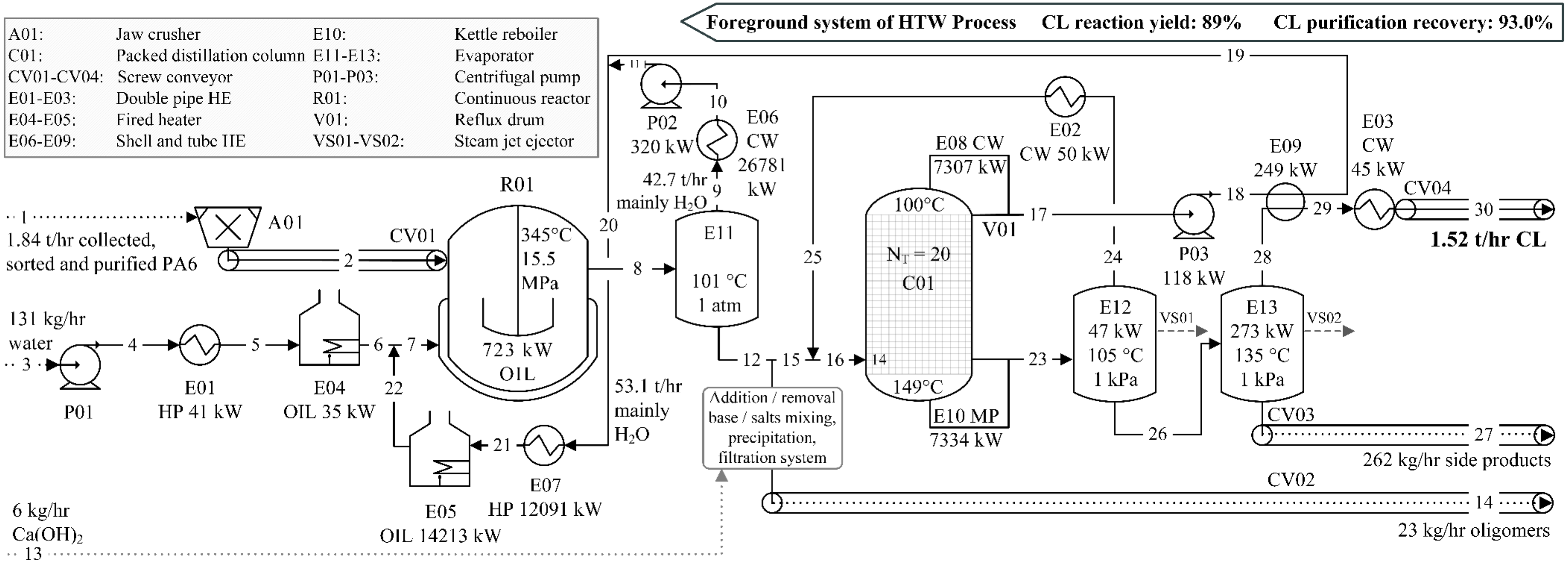

Figure B 3: schematic process flow diagram of the HTW process.

Table B 4: stream table of the HTW process including information on phases, temperatures, pressures, mass flows, mass fractions and enthalpy flows of the streams.

Stream Name		Units	1	2	3	4	5	6	7	8	9	10
Phase		-	Solid	Solid	Liquid	Liquid	Liquid	Liquid	Liquid	Liquid	Vapor	Liquid
Temperature		°C	25.00	25.00	25.00	37.68	250.00	344.62	344.62	344.99	100.69	100.02
Pressure		bar	1.01	1.01	1.01	155.42	155.12	154.82	154.82	154.82	1.01	1.01
Mass Flows	TOTAL	kg/hr	1837.38	1837.38	135.79	135.79	135.79	135.79	55137.71	56967.00	43650.23	43650.23

Mass Fractions	CL	-	0.00	0.00	0.00	0.00	0.00	0.00	1.47E-04	0.03	1.85E-04	1.85E-04
	PA6	-	1.00	1.00	0.00	0.00	0.00	0.00	0.00	0.00	0.00	0.00
	WATER	-	0.00	0.00	1.00	1.00	1.00	1.00	1.00	0.97	1.00	1.00
	ACA	-	0.00	0.00	0.00	0.00	0.00	0.00	1.00E-05	3.56E-04	1.28E-05	1.28E-05
	ACADIMER	-	0.00	0.00	0.00	0.00	0.00	0.00	0.00	0.00	0.00	0.00
	CAOH	-	0.00	0.00	0.00	0.00	0.00	0.00	0.00	0.00	0.00	0.00
	ACA*CA	-	0.00	0.00	0.00	0.00	0.00	0.00	0.00	0.00	0.00	0.00
Enthalpy Flow		kW	-1622.22	-1622.22	-598.39	-596.41	-554.73	-519.74	-211014.04	-211917.59	-161006.04	-188405.27

**Stream Name**		**Units**	**11**	**12**	**13**	**14**	**15**	**16**	**17**	**18**	**19**	**20**
Phase		-	Liquid	Liquid	Liquid	Liquid	Liquid	Mixed	Liquid	Liquid	Liquid	Liquid
Temperature		°C	105.89	100.69	25.00	100.69	100.69	7.46	100.02	108.13	124.90	109.88
Pressure		bar	155.42	1.01	1.01	1.01	1.01	0.01	1.01	155.72	155.42	155.42
Mass Flows	TOTAL	kg/hr	43650.23	13316.77	5.56	23.14	13299.19	13555.10	11352.99	11352.99	11352.99	55003.23
Mass Fractions	CL	-	1.85E-04	0.12	0.00	0.02	0.12	0.14	1.66E-06	1.66E-06	1.66E-06	1.47E-04
	PA6	-	0.00	0.00	0.00	0.00	0.00	0.00	0.00	0.00	0.00	0.00
	WATER	-	1.00	0.86	0.00	0.00	0.86	0.85	1.00	1.00	1.00	1.00
	ACA	-	1.28E-05	0.00	0.00	1.30E-06	0.00	0.00	0.00	0.00	0.00	1.01E-05
	ACADIMER	-	0.00	0.01	0.00	0.00	0.01	0.01	0.00	0.00	0.00	0.00
	CAOH	-	0.00	0.00	1.00	0.00	0.00	0.00	0.00	0.00	0.00	0.00
	ACA*CA	-	0.00	0.00	0.00	0.97	0.00	0.00	0.00	0.00	0.00	0.00
Enthalpy Flow		kW	-188078.9	-50911.55	-13.35	-11.47	-50875.82	-51067.45	-49010.38	-48892.87	-48644.02	-236722.93

**Stream Name**	**Units**	**21**	**22**	**23**	**24**	**25**	**26**	**27**	**28**	**29**	**30**

Phase		-	Liquid	Liquid	Liquid	Vapor	Liquid	Liquid	Liquid	Vapor	Mixed	Liquid
Temperature		°C	250.00	344.62	149.38	105.00	69.91	105.00	40.00	135.00	124.90	40.00
Pressure		bar	155.12	154.82	1.01	0.01	0.01	0.01	1.01	0.01	0.01	1.01
Mass Flows	TOTAL	kg/hr	55003.23	55003.23	2202.11	417.16	255.90	1784.96	261.44	1523.52	1523.52	1522.21
Mass Fractions	CL	-	1.47E-04	1.47E-04	0.85	0.67	1.00	0.90	0.30	1.00	1.00	1.00
	PA6	-	0.00	0.00	0.00	0.00	0.00	0.00	0.00	0.00	0.00	0.00
	WATER	-	1.00	1.00	0.06	0.33	3.03E-04	4.85E-04	3.38E-06	5.67E-04	5.67E-04	8.88E-05
	ACA	-	1.01E-05	1.01E-05	0.00	0.00	0.00	0.00	0.00	0.00	0.00	0.00
	ACADIMER	-	0.00	0.00	0.08	1.95E-08	3.18E-08	0.10	0.70	3.66E-07	3.66E-07	3.66E-07
	CAOH	-	0.00	0.00	0.00	0.00	0.00	0.00	0.00	0.00	0.00	0.00
	ACA*CA	-	0.00	0.00	0.00	0.00	0.00	0.00	0.00	0.00	0.00	0.00
Enthalpy Flow		kW	-224669.48	-210499.28	-2029.64	-661.79	-191.63	-1322.48	-219.93	-844.58	-1093.43	-1161.27

**Stream Name**		**Units**	**Volatiles of VS01**	**Volatiles of VS02**
Phase		-	Vapor	Vapor
Temperature		°C	69.91	74.84
Pressure		bar	0.01	0.01
Mass Flows	TOTAL	kg/hr	163.60	3.75
Mass Fractions	CL	-	0.15	0.15
	PA6	-	0.00	0.00
	WATER	-	0.83	0.19
	ACA	-	0.00	0.00
	ACADIMER	-	0.00	0.00
	CAOH	-	0.00	0.00
	ACA*CA	-	0.00	0.00

		rest: air	rest: air
Enthalpy Flow	kW	-519.58	-3.00

B.3.2.3 iPrOH Process

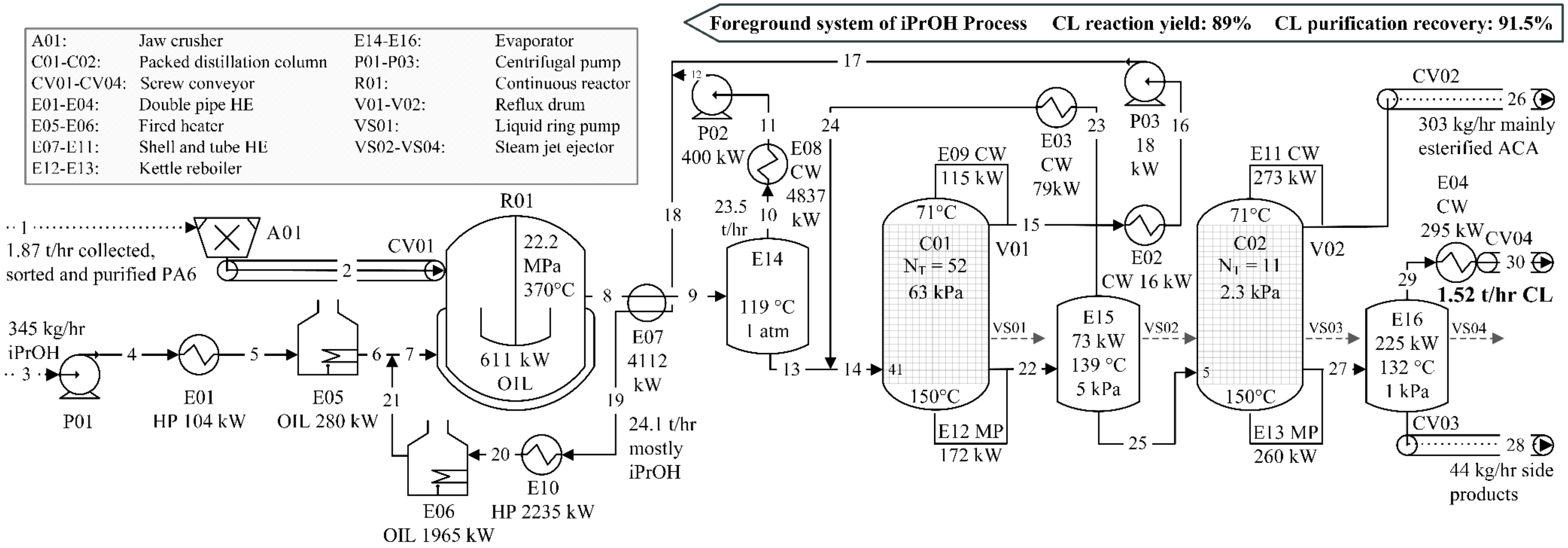

Figure B 4: schematic process flow diagram of the iPrOH process.

Table B 5: stream table of the iPrOH process including information on phases, temperatures, pressures, mass flows, mass fractions and enthalpy flows of the streams.

Stream Name	Units	1	2	3	4	5	6	7	8	9	10
Phase	-	Solid	Solid	Liquid	Liquid	Liquid	Liquid	Liquid	Liquid	Liquid	Vapor
Temperature	°C	25.00	25.00	25.00	60.29	250.00	370.00	370.00	360.63	218.04	119.33

Pressure		bar	1.01	1.01	1.01	255.60	255.30	255.00	255.00	222.00	221.70	1.01
Mass Flows	TOTAL	kg/hr	1868.78	1868.78	345.09	345.09	345.09	345.09	24494.35	26363.37	26363.37	23564.69
Mass Fractions	CL	%	0.00%	0.00%	0.00%	0.00%	0.00%	0.00%	0.37%	6.31%	6.31%	0.39%
	PA6	%	100.00%	100.00%	0.00%	0.00%	0.00%	0.00%	0.00%	0.00%	0.00%	0.00%
	iPrOH	%	0.00%	0.00%	100.00%	100.00 %	100.00%	100.00%	99.18%	91.56%	91.56%	99.15%
	Est ACA	%	0.00%	0.00%	0.00%	0.00%	0.00%	0.00%	0.44%	2.12%	2.12%	0.46%
	ACADIMER	%	0.00%	0.00%	0.00%	0.00%	0.00%	0.00%	0.00%	0.01%	0.01%	0.00%
Enthalpy Flow		kW	-1649.95	-1649.95	-507.52	-497.01	-393.14	-365.13	-25783.93	-26790.56	-30902.21	-28457.93

**Stream Name**		**Units**	**11**	**12**	**13**	**14**	**15**	**16**	**17**	**18**	**19**	**20**
Phase		-	Liquid	Liquid	Liquid	Liquid	Liquid	Liquid	Liquid	Liquid	Liquid	Liquid
Temperature		°C	82.14	99.08	119.33	113.79	70.63	37.33	72.21	98.47	214.72	250.00
Pressure		bar	1.01	255.90	1.01	1.01	0.63	0.63	255.90	255.90	255.60	255.30
Mass Flows	TOTAL	kg/hr	23564.69	23564.69	2798.68	3197.33	588.93	582.55	582.55	24149.26	24149.26	24149.26
Mass Fractions	CL	%	0.39%	0.39%	56.16%	54.07%	0.00%	0.00%	0.00%	0.38%	0.38%	0.38%
	PA6	%	0.00%	0.00%	0.00%	0.00%	0.00%	0.00%	0.00%	0.00%	0.00%	0.00%
	iPrOH	%	99.15%	99.15%	27.64%	25.80%	99.99%	99.96%	99.96%	99.17%	99.17%	99.17%
	Est ACA	%	0.46%	0.46%	16.09%	20.02%	0.01%	0.01%	0.01%	0.45%	0.45%	0.45%
	ACADIMER	%	0.00%	0.00%	0.12%	0.10%	0.00%	0.00%	0.00%	0.00%	0.00%	0.00%
Enthalpy Flow		kW	-33294.56	-32894.69	-2444.28	-2730.9	-842.53	-850.46	-832.33	-33730.11	-29618.47	-27383.90

**Stream Name**	**Units**	**21**	**22**	**23**	**24**	**25**	**26**	**27**	**28**	**29**	**30**
Phase	-	Liquid	Liquid	Vapor	Liquid	Liquid	Liquid	Liquid	Liquid	Vapor	Liquid
Temperature	°C	370.00	149.90	139.00	68.14	139.00	40.00	149.56	40.00	131.91	40.00

Pressure		bar	253.60	0.63	0.06	0.05	0.06	1.01	0.02	1.01	0.01	1.01
Mass Flows	TOTAL	kg/hr	24149.26	2608.40	578.65	398.65	2029.76	455.60	1567.73	44.05	1523.68	1523.41
Mass Fractions	CL	-	0.38%	66.27%	27.26%	39.40%	77.39%	1.72%	99.70%	92.48%	99.91%	99.91%
	PA6	-	0.00%	0.00%	0.00%	0.00%	0.00%	0.00%	0.00%	0.00%	0.00%	0.00%
	iPrOH	-	99.17%	9.06%	39.41%	12.94%	0.40%	0.61%	0.00%	0.00%	0.00%	0.00%
	Est ACA	-	0.45%	24.54%	33.33%	47.66%	22.04%	97.67%	0.09%	0.01%	0.09%	0.09%
	ACADIMER	-	0.00%	0.13%	0.00%	0.00%	0.16%	0.00%	0.21%	7.51%	0.00%	0.00%
Enthalpy Flow		kW	-25418.80	-1831.13	-427.54	-286.62	-1330.90	-234.20	-1108.83	-41.83	-843.84	-1161.40

Stream Name		Units	Volatiles of VS01	Volatiles of VS02	Volatiles of VS03	Volatiles of VS04
Phase		-	Vapor	Vapor	Vapor	Vapor
Temperature		°C	37.33	68.14	66.04	69.94
Pressure		bar	0.63	0.05	0.02	0.01
Mass Flows	TOTAL	kg/hr	19.75	182.32	9.40	2.73
Mass Fractions	CL	-	0.00%	0.37%	0.19%	10.14%
	PA6	-	0.00%	0.00%	0.00%	0.00%
	iPrOH	-	33.03%	96.79%	57.09%	0.00%
	Est ACA	-	0.00%	1.56%	11.21%	0.12%
	ACADIMER	-	0.00%	0.00%	0.00%	0.00%
			rest: air	rest: air	rest: air	rest: air
Enthalpy Flow		kW	-8.13	-220.08	-7.03	-0.13

B.3.2.4 NaOH Process

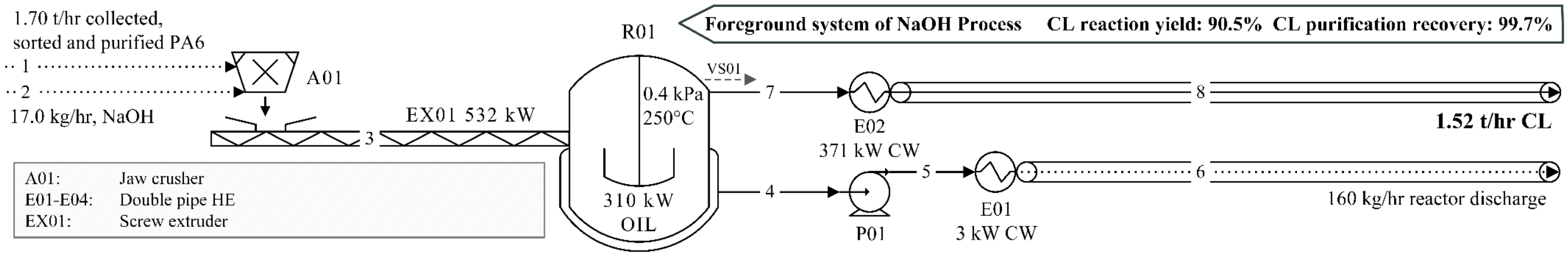

Figure B 5: schematic process flow diagram of the NaOH process.

Table B 6: stream table of the NaOH process including information on phases, temperatures, pressures, mass flows, mass fractions and enthalpy flows of the streams.

**Stream Name**		**Units**	**1**	**2**	**3**	**4**	**5**	**6**	**7**	**8**	**Volatiles of VS01**
Phase		-	Solid	Liquid	Liquid	Liquid	Liquid	Liquid	Vapor	Liquid	Vapor
Temperature		°C	25.00	25.00	219.86	250.00	250.01	40.00	250.00	40.00	89.15
Pressure		bar	1.01	1.01	1.01	0.00	1.37	1.01	0.00	1.01	0.00
Mass Flows	TOTAL	kg/hr	1686.45	17.04	1703.49	177.22	177.22	177.22	1526.26	1522.07	7.38
	CL	kg/hr	0.00	0.00	0.00	0.63	0.63	0.63	1526.26	1522.07	4.19
	PA6	kg/hr	1686.45	0.00	1686.45	159.56	159.56	159.56	trace	0.00	0.00
	NaOH	kg/hr	0.00	17.04	17.04	17.04	17.04	17.04	0.00	0.00	trace
Mass Fractions	CL	-	0.00	0.00	0.00	0.00	0.00	0.00	1.00	1.00	0.57
	PA6	-	1.00	0.00	0.99	0.90	0.90	0.90	trace	0.00	0.00
	NaOH	-	0.00	1.00	0.01	0.10	0.10	0.10	52.3463 PPB	52.4902 PPB	trace
Enthalpy Flow		kW	-1488.96	-33.31	-1206.03	-142.33	-142.33	-164.83	-754.03	-1160.74	-2.34

C Inventory Analysis

C.1 Equipment

The equipment inventories were established either by calculating equipment dimensions and subsequently determining the required material amounts (e.g., steel, aluminum) based on these calculations, or by directly utilizing available datasets from ecoinvent for specific equipment types. For cases involving calculations, the determined material amounts were incorporated into the inventory using ecoinvent datasets. Equipment dimensions were determined using equations provided by Seader et al. in their work on chemical process design [20]. These equations, tailored for various types of equipment, account for specific design parameters and operating conditions. The corresponding equations for each equipment type are detailed in the following sections, addressing aspects such as vessel sizing, heat exchanger design, and column dimensions, among others.

Since the weight calculations by Seader et al. primarily focus on the main structural components of the equipment, such as steel, and do not include materials required for auxiliary purposes (e.g., controlling the equipment, concrete foundations, electricity cables, or insulation materials like glass wool), we utilized the methodology developed by Castellanos-Beltran et al. (2023) [21]. This offers regressed formulas to estimate the LCI of specific equipment types, such as heat exchangers, towers, and flash tanks, covering manufacturing, transportation, operation, and disposal stages with regressed functions to calculate material requirements for lifecycle inventories of all components beyond steel. These equations enable a systematic estimation of the weights of additional structural components (e.g., concrete, aluminum) and functional components, facilitating comprehensive integration into LCIs and enabling detailed assessments of material use and environmental impacts.

Ecoinvent datasets are referenced to align with these material specifications: carbon steel is represented by low-alloy steel, while stainless steel corresponds to chromium steel 18/8.

C.1.1 Jaw Crusher

The material production of jaw crushers used in the processes were excluded from the analysis. This decision was made because they are not considered main equipment, equations for calculating their dimensions are not provided by Seader et al., and no datasets specific to jaw crusher production are available in ecoinvent.

C.1.2 Reactor

The weight calculation of a reactor as a horizontal vessel can be seen in the following Table A 7. Depending on the pathway, a different material for the reactor production was assumed, e.g. Hastelloy for the H_3PO_4 process and stainless steel for the iPrOH, HTW and NaOH processes.

Table A 7: Equipment dimension calculation of horizontal vessels such as reactors according to Seader et al. [20,22].

Meaning			Calculation
Height to diameter ratio	:	ratio	3 (from heuristics, Seider et al. (2008)[20])
Volume	:	V	From Aspen Plus Simulation, liquid volume
Inside Diameter	:	D_i	$D_i = \left(\frac{4 \cdot V}{\pi \cdot \text{ratio}}\right)^{\frac{1}{3}}$
Height	:	H	$H = D_i \cdot$ ratio (height to diameter)
Operating pressure	:	P_o [psig]	From Aspen Plus Process Simulation
Design Pressure :	:	P_d	For P_o between 0 and 5: $P_d = 10$<br>For P_o between 10 and 1000 psig:<br>$P_d = \exp\{0.60608 + 0.91615[\ln(P_o)] + 0.0015655[\ln(P_o)]^2\}$<br>For P_o bigger than 1000 psig: $P_d = 1.1\ P_o$
Operating temperature	:	T_o	From Aspen Plus Process Simulation

	[°F]	
Design temperature :	T_d [°F]	$T_d = T_o + 50\ °F$
Modulus of elasticity :	E_M [psi]	For -20 < T_d <= 200 : E_M = 30.2 10^6<br>For 200 < T_d <= 400 : E_M = 29.5 10^6<br>For 400 < T_d <= 650 : E_M = 28.3 10^6<br>For T_d > 650 : E_M = 26 10^6
Maximum allowable stress :	S [psi]	For -20 < T_d <= 750 : S = 15000<br>For 750 < T_d <= 800 : S = 14750<br>For 800 < T_d <= 850 : S = 14200<br>For 850 < T_d <= 900 : S = 13100
Wall thickness : Correction factor : Total wall thickness :	t_E<br>t_{EC}<br>t_t<br>[in]	For atmospheric or high pressures: H and D in inches<br>$t_t = \frac{P_d \cdot D_i}{2 \cdot S \cdot E - 1.2 \cdot P_d}$<br>For vacuum operation: H and D in inches<br>$t_E = 1.3 \cdot (D_i + t_E) \cdot \left(\frac{P_d \cdot H}{E_M \cdot (D_i + t_E)}\right)^{0.4}$ for $\frac{t_E}{D_i} \leq 0.05$<br>$t_{EC} = H \cdot (0.18 \cdot D_i - 2.2) \cdot 10^{-5} - 0.19$<br>$t_t = t_E + t_{EC} + 0.125$
Weight of shell and two heads :	W [lb]	$W = \pi\,(D_i + t_t)(H + 0.8\,D_i)\,t_t\,\rho$<br>H and D in inches , ρ_{carbon} in lb/in^3

C.1.3 Distillation Column Vessel and Reflux Drum

For distillation columns, structured packed designs were assumed for vacuum operations, while trayed designs were used for atmospheric pressure operations. For the trayed columns, sieve trays with hole diameters of 0.6 to 0.7 cm were assumed. The tray spacing was set at 0.5 m, with additional space allocated at the top and bottom of the column, set to 1.2 m and 1.8 m, respectively.

The diameter of a distillation column modelled with the DWSTU in Aspen Plus is calculated through the flooding velocity and assuming an operation mode at 80% of flooding [23]. With the calculated diameter, the flooding factor was maintained between 0.4 and 0.8 on each tray. The active area of the trays was set to 90% to reflect standard design practices.

For columns modeled using RADFRAC in Aspen Plus, the diameter was directly taken from the software's output without additional calculations.

The following table gives the calculation procedure of the steel weight of the distillation column based on Seader et al. [20].

Table A 8: Equipment dimension calculation of the distillation columns [20,22].

Meaning		Formulas
Number of trays :	N	Aspen Process Optimisation
Height :	H	H = 0.5 N + 3
Tangent-to-tangent length :	L	L = 0.5 N
Diameter Calculation for DWSTU Column:		
Reflux ratio :	RR	From Aspen Plus Simulation
Distillate :	D [kg/s]	From Aspen Plus Simulation
Liquid flowrate :	L [kg/s]	L = R · D

Vapor flowrate top of column	:	V [kg/s]	V=D · (1+RR)+F
Vapor density	:	ρ_V [kg/m³]	From Aspen Plus Simulation
Liquid density	:	ρ_L [kg/m³]	From Aspen Plus Simulation
Flow parameter	:	F_{LV}	$\frac{L}{V} \cdot \left(\frac{\rho_V}{\rho_L}\right)^{0.5}$
Flooding capacity factor	:	$C_{sb,f}$	$\log_{10} C_{sb,f} = -1.0262 - 0.63513 \cdot \log_{10}(F_{LV}) - 0.20097 \cdot \log_{10}(F_{LV})^2$
Flooding velocity	:	u_f [ft/s]	$u_f = C_{sb,f} \cdot 1.3 \cdot \left(\frac{\rho_V}{\rho_L - \rho_V}\right)^{0.5}$
Vapor velocity	:	u_V	$u_V = 0.8 \cdot u_f$
Active area	:	A	$A = \frac{V}{\rho_V \cdot u_V}$
Diameter	:	D	$D = \left(\frac{4 \cdot A}{\pi}\right)^{\frac{1}{2}}$
Lowest pressure :	:	P_o [psig]	Aspen Plus Process Simulation
Design Pressure :	:	P_d [psig]	For P_o between 0 and 5: $P_d = 10$<br>For P_o between 10 and 1000 psig:<br>$P_d = \exp\{0.60608 + 0.91615[\ln(P_o)] + 0.0015655[\ln(P_o)]^2\}$<br>For P_o bigger than 1000 psig: $P_d = 1.1\ P_o$
Highest operating temperature	:	T_o [°F]	Aspen Plus Process Simulation
Design temperature	:	T_d [°F]	$T_d = T_o + 50\ °F$
Modulus of elasticity	:	E_M [psi]	For $-20 < T_d <= 200$: $E_M = 30.2\ 10^6$<br>For $200 < T_d <= 400$: $E_M = 29.5\ 10^6$<br>For $400 < T_d <= 650$: $E_M = 28.3\ 10^6$<br>For $T_d > 650$: $E_M = 26\ 10^6$
Maximum allowable stress	:	S [psi]	For $-20 < T_d <= 750$: S = 15000<br>For $750 < T_d <= 800$: S = 14750<br>For $800 < T_d <= 850$: S = 14200<br>For $850 < T_d <= 900$: S = 13100
Wall thickness<br>Correction factor<br>Total wall thickness	:<br>:<br>:	t_E<br>t_{EC}<br>t_t<br>[in]	For atmospheric or high pressures: H and L in inches<br>$t_{E1} = \frac{0.22\ ((D_i + t_{E1}) + 18)\ L^2}{S\ (D_i + t_{E1})^2}$<br>$t_{E2} = \frac{P_d \cdot D_i}{2 \cdot S \cdot E - 1.2 \cdot P_d}$<br>$t_t = \frac{t_{E1} + t_{E2}}{2}$<br>For vacuum operation: H and L in inches<br>$t_E = 1.3 \cdot (D_i + t_E) \cdot \left(\frac{P_d \cdot L}{E_M \cdot (D_i + t_E)}\right)^{0.4}$ for $\frac{t_E}{D_i} \leq 0.05$<br>$t_{EC} = L \cdot (0.18 \cdot D_i - 2.2) \cdot 10^{-5} - 0.19$<br>$t_t = t_E + t_{EC} + 0.125$
Weight of shell and two heads	:	W	$W = \pi\ (D + t_S)\ (L + 0.8\ D)\ t_S\ \rho$<br>L and D in inches , ρ in lb/in³

Reflux drums are in a horizontal cylindrical shape with an optimum length to diameter ratio of three. The liquid hold up of the reflux drum was set to 5 minutes with a liquid level of 50% [20]. The following table gives the calculation procedure of the steel weight of the reflux drum based on Seader et al. [20].

Table A 9: Equipment dimension calculation of the reflux drum [20,22].

Meaning	Formulas
Liquid flowrate	$\dot{V}_L$ from Aspen Plus Simulation
Holdup volume	$V_{ho} = t_{ho} . \dot{V}_L$<br>$with\ t_{ho} = 300 \ \ (heuristic)$
Volume vessel	$V_{ves} = \frac{V_{ho}}{0.5}$
Diameter vessel	$D_{ves} = \sqrt[3]{\frac{4\ V_{ves}}{3\ \pi}}$
Length vessel	$L_{ves} = 3\ D_{des} \ \ (heuristic)$
Lowest pressure	Aspen Plus Process Simulation
Design Pressure vacuum	$P_d = \exp\{0.60608 + 0.91615[\ln(P_o)] + 0.0015655[\ln(P_o)]^2\}$<br>For operating pressure between 10 and 1000 psig<br>$P_d = 10$<br>For operating pressure between 0 and 5 psig
Highest temperature	Aspen Plus Process Simulation
Design Temperature	$T_d = T_{highest} + 50\ °F$
Maximum allowable stress	For -20 < T_d <= 750 : S = 15000<br>For 750 < T_d <= 800 : S = 14750<br>For 800 < T_d <= 850 : S = 14200<br>For 850 < T_d <= 900 : S = 13100
Modulus of elasticity	For -20 < T_d <= 200 : $E_M = 30.2\ \ 10^6$<br>For 200 < T_d <= 400 : $E_M = 29.5\ \ 10^6$<br>For 400 < T_d <= 650 : $E_M = 28.3\ \ 10^6$<br>For T_d > 650 : $E_M = 26\ \ 10^6$
Total wall thickness	For atmospheric or high pressures: L and D in inches<br>$t_t = \frac{P_d \cdot D_i}{2 \cdot S \cdot E - 1.2 \cdot P_d}$<br><br>For vacuum operation: L and D in inches<br>$t_E = 1.3 \cdot (D_i + t_E) \cdot \left(\frac{P_d \cdot L}{E_M \cdot (D_i + t_E)}\right)^{0.4}$ for $\frac{t_E}{D_i} \leq 0.05$<br>$t_{EC} = L \cdot (0.18 \cdot D_i - 2.2) \cdot 10^{-5} - 0.19$<br>$t_t = t_E + t_{EC} + 0.125$
Weight of shell and two heads	$W = \pi\ (D + t_S)\ (L + 0.8\ D)\ t_S\ \rho_{carbon}$<br>L and D in inches , $\rho_{carbon} = 0.284\ lb/in^3$

Based on the calculated steel weights of column and reflux drum given by the previous tables, the weight of concrete, powering, glasswool and aluminum was calculated based on Castellanos-Beltran et al. (2023) [21] as follows:

Concrete calculation: Weight $_{concrete}$ (kg) $=$ Weight $_{steel}$ $\cdot 2.63$

Powering and electronics calculation: Weight $_{powering}$ (kg) $=$ Weight $_{steel}$ $\cdot\ 0.01$

Glass wool calculation: Weight $_{glass\ wool}$ (kg) $= -26.9 \times D + 4.753 \times N_{th} + 47.56 \times D^2 + 31.96 \times [D \times N_{th}] + 24.53$

Aluminium calculation:

Weight $_{aluminium}$ (kg) $= 30.67 \times \mathrm{D} + 11.25 \times \mathrm{N_{th}} + 53.86 \times \mathrm{D}^2 + 40.4 \times [\mathrm{D} \times \mathrm{N_{th}}] + 4.367$

C.1.4 Filter

The material production of filters used in the processes were excluded from the analysis. This decision was made because they are not considered main equipment, equations for calculating their dimensions are not provided by Seader et al., and no datasets specific to jaw crusher production are available in ecoinvent.

C.1.5 Heat Exchanger

The type of heat exchanger was chosen according to heuristics of Seider et al. (2008) which was incorporated in the Python code and can be seen in the following table [20,22]. The table below provides an overview of the key parameters used for sizing heat exchangers based on established heuristics and design standards.

Shell and tube heat exchangers are chosen to operate countercurrently. Heat exchangers are designed as a 16-ft long tube with an outside diameter of ¾-in and a 1-in tube pitch in triangular spacing, as it is the standard design [20]. The tube side is for fouling, corrosive, scaling and high-pressure fluids while the shell side is for viscous, evaporating or condensing fluids. The countercurrent flow correction factor is 0.9 in a conservative estimate.

The shell diameter is estimated to be 30% larger than the tube bundle diameter to ensure adequate clearance for maintenance and optimal flow dynamics. The baffle cut is set at 25%, following the recommendation of segmental baffles with a height equal to 75% of the shell diameter. This configuration supports efficient heat transfer while maintaining structural integrity.

Table A 10: Heat exchanger dimension calculation and material requirement calculation [20,22].

Meaning	Formula
Heat load	From Aspen Plus or calculation through: $Q = c_p \cdot \dot{m} \cdot \Delta T$
Logarithmic mean	From Aspen Plus or calculation through: $\Delta T_{LM} = \frac{\Delta T_1 - \Delta T_2}{\ln\left(\frac{\Delta T_1}{\Delta T_2}\right)}$
Heat transfer coefficient	water – liquid: 850 W/m^2/°C<br>liquid – liquid: 280 W/m^2/°C<br>gas – gas: 30 W/m^2/°C<br>reboiler: 1140 W/m^2/°C<br>water – water: 1140 W/m^2/°C<br>liquid – condensing vapour: 850 W/m^2/°C
Correction factor	$\mathrm{F} = 0.9$ (heuristic Seider et al. (2008)20)
Area	$\mathrm{A} = \frac{\mathrm{Q}}{\mathrm{U} \cdot \Delta \mathrm{T_{LM}} \cdot \mathrm{F}}$
Tube length	$L_{TU} = 4.8768$ m (default according to heuristic)
Tube outer diameter	$D_{TU,OD} = 0.01905$ m (default according to heuristic)
Tube thickness	$WT_{TU} = 0.00211$ m (default)
Tube inner diameter	$D_{TU,ID} = D_{TU,OD} - (2 \cdot WT_{TU})$
Tube number	$N_{TU} = \frac{A}{\pi \cdot D_{TU,OD} \cdot L_{TU}}$ rounded up to the nearest whole number
Tube density	$\rho_{TU} = 7850 \frac{kg}{m^3}$ (default: carbon steel)
Tube weight	$W_{TU} = \pi \cdot N_{TU} \cdot \left(\frac{D_{TU,OD}^2 - D_{TU,ID}^2}{4}\right) \cdot L_{TU} \cdot \rho_{TU}$
Pitch factor	$F_{pitch} = 1.1$ for triangular pitch (default according to heuristic)<br>$F_{pitch} = 1.25$ for square pitch
Pitch	$\mathrm{pitch} = F_{pitch} \cdot D_{TU,OD}$
Shell diameter	$D_{shell,OD} = 1.3 \cdot \sqrt{\frac{N_{TU} \cdot pitch^2}{\pi}}$
Number of baffles	$N_{baff} = D_{shell,OD} / 0.9$ rounded to the nearest whole number.

Shell thickness	$WT_{shell} = 0.0127$ m (default)
Shell inner diameter	$D_{shell,ID} = D_{shell,ID} - (2 \cdot WT_{shell})$
Shell volume	$V_{shell} = \pi \cdot \left(\frac{D_{shell,OD}^{2} - D_{shell,ID}^{2}}{4}\right) \cdot L_{TU}$
Percentage of baffle cut	$Cut_{baff} = 15$ % (default according to heuristic)
Baffle spacing	$Space_{baff} = \frac{D_{shell}}{N_{baff} + \left(\frac{Cut_{baff}}{100}\right)}$
Baffle thickness	$WT_{baff} = 0.00635$ (default)
Baffle density	$\rho_{shell} = 7850 \frac{kg}{m^3}$ (default: carbon steel)
Baffle weight	$W_{baff} = \frac{\pi \cdot D_{shell,ID}^{2}}{4} \cdot WT_{baff} \cdot \frac{L_{TU}}{Space_{baff}} \cdot \rho_{shell}$
Shell weight	$W_{shell} = V_{shell} \cdot \rho_{shell} + W_{baff}$

Based on shell and tube weights, the other material requirements can be calculated according to Castellanos-Beltran et al. (2023) [21] with the equations displayed in the following equations.

Glass Wool Weight Calculation:

$$A^{0.488} \cdot (39.1 - 13.08 \cdot \left(\frac{D_{TU,OD} \cdot 1000}{19.05} + 1.707 \cdot \left(\frac{D_{TU,OD} \cdot 1000}{19.05}\right)^{2}\right)$$

Aluminium Weight Calculation:

$$A^{0.3565} \cdot (95.14 - 25.62 \cdot \left(\frac{D_{TU,OD} \cdot 1000}{19.05} + 3.234 \cdot \left(\frac{D_{TU,OD} \cdot 1000}{19.05}\right)^{2}\right)$$

Total Concrete Heat Exchanger Weight:

$$(W_{shell} + W_{TU}) \cdot 2.63$$

Total Powering and Electronics Calculation:

$$(W_{shell} + W_{TU}) \cdot 0.01$$

C.1.6 Pumps

The ecoinvent database includes datasets for pump production, which were utilized and are shown in the inventory tables in Section C.3.

C.1.7 Screw Conveyor

The ecoinvent database includes datasets for conveyor production, which were utilized and are shown in the inventory tables in Section C.3.

C.1.8 Extruder

For the LCI, a large extruder with a throughput exceeding 1000 kg/hr was incorporated. Based on the requirements, the largest screw diameter (150 mm) was selected. The screw design includes flights (helical blades) and channels (spaces between the flights), which were modeled to occupy 60% of the volume of a solid cylinder.

To account for proper clearance and insulation, an additional 10% of the screw diameter was added to calculate the internal diameter of the barrel. The wall thickness of the barrel was set at 25 mm. The calculations for the screw and barrel volumes, as well as the total weight of the extruder, are as follows:

Table C 1: Equipment dimension and material requirement calculation of the extruder.

**Meaning**	**Formula**

Screw diameter	$D_{screw} = 0.15$ m
Length-to-Diameter ratio	$LD_{ratio} = 30$
Barrel wall thickness	$WT_{barrel} = 0.25$ m
Barrel inner diameter	$D_{barrel,ID} = 1.1 \cdot D_{screw}$
Barrel outer diameter	$D_{barrel,OD} = D_{barrel,ID} + 2 \cdot WT_{barrel}$
Screw volume	$V_{screw} = \pi \cdot 0.6 \cdot \frac{D_{screw}}{2}^2 \cdot D_{screw} \cdot LD_{ratio}$
Barrel volume	$V_{barrel} = \pi \cdot \frac{D_{barrel,OD}}{2}^2 \cdot D_{screw} \cdot LD_{ratio} - \pi \cdot \frac{D_{barrel,ID}}{2}^2 \cdot D_{screw} \cdot LD_{ratio}$
Total weight of extruder	$V_{ex} = (V_{screw} + V_{barrel}) \cdot \rho_{steel}$

C.1.9 Vacuum System

The environmental impacts associated with the production of vacuum pumps were approximated using data for the production of conventional pumps, as detailed in Section C.1.6.

C.1.10 Piping, Instrumentation and Control

To account for additional components such as piping, instrumentation, and control, the total equipment steel weight was multiplied by a factor of 1.57 [20].

C.1.11 Chemical Factory

Piping, instrumentation, and control systems, as well as land use, were incorporated using a dataset from ecoinvent representing a typical chemical facility, frequently used for assessing the impacts of chemical plants [24,25]. The "chemical factory construction" activity was utilized as Ecoinvent dataset. With the calculated steel weight requirements (see sections above), the chemical factory construction activity was adjusted ensuring the steel content corresponds to the actual calculated value. This scaling proportionally adjusts all other components of the activity. Since the steel and concrete amounts were already directly incorporated into the process equipment modeling, these quantities were subtracted from the activity to avoid double-counting.

Land use was addressed using the "chemical factory construction, organics" activity. This activity inherently includes the "chemical factory construction" as part of its inputs. The "chemical factory construction, organics" activity was scaled similarly to the "chemical factory construction" activity—based on the known activity amount of "chemical factory construction".

C.1.12 Raw Materials

The recycling solvent required to fill the reactor (intital solvent charge), based on its residence time and volume, was assumed to be replaced once per year.

C.2 Utility Calculations

C.2.1 Equipment Utility Requirements

The following equipment types were not simulated in Aspen Plus. Hence the duty needed to be calculated manually.

C.2.1.1 Steam Jet Ejector

For three-stage steam jet ejector, the following equations are used to calculate the amount of MP steam [20].

$$\text{For } p > 15 \text{ kPa:} \quad \dot{m}_s = 10\, W_{vacuum} \tag{1}$$

For p < 15 kPa: $\dot{m}_s = 100\ W_{vacuum}$ (2)

C.2.1.2 Liquid Ring Pump

Electricity consumption of liquid ring pump is calculated as follows:

$$\dot{Q} = \Delta p \cdot W_{vacuum} \tag{3}$$

C.2.1.3 Jaw Crusher

Electricity consumption of jaw crusher is calculated using the mass flow $\dot{m}$ [26]:

$$\dot{Q} = 0.23 \cdot \dot{m} \qquad (\dot{m}\text{ in ton/s}) \tag{4}$$

C.2.1.4 Screw Conveyor

Electricity consumption of screw conveyor is calculated as follows [20]:

$$\dot{Q} = 0.00146 \cdot \dot{m}^{0.85} \cdot L \qquad (\dot{m}\text{ in }\frac{\text{lb}}{\text{s}}\text{, L in ft}) \tag{5}$$

C.2.1.5 Reactor Turbine Agitator

Electricity consumption of the agitator is calculated according to a heuristic in Seider at al. (2008) in HP with the volume of the reactor in gallons [20]:

$$\dot{Q} = 0.01 \cdot volume \qquad (\text{volume in gallons, Q in HP}) \tag{6}$$

C.2.1.6 Extruder

Electricity consumption of the extruder is calculated as follows [27,28]:

$$\dot{Q} = 0.0053 \cdot \dot{m} \cdot \text{cp} \cdot \Delta T \qquad (\dot{Q}\ in\ HP, \dot{m}\text{ in }\frac{lb}{hr}\text{, cp in }\frac{\text{BTU}}{lb\ F}\text{, }\Delta\text{T in F}) \tag{7}$$

C.2.2 Conditions

C.2.2.1 Cooling Water

The cooling water amount was calculated according to equation (8), where cp_w is the heat capacity of water, and ΔT_w the temperature difference of the cooling water inlet and outlet stream [29].

$$\dot{m}_w = \dot{Q}_{HE} / cp_w \cdot \Delta T_w \tag{8}$$

Table B 7: Characteristics of cooling water that is taken as utility in this project

	T [°C]		c_{pw} [kJ/kg/K]	Costs [\$/m^3]
	In	out		
Cooling water :	30	45	4.2	0.02 [20]

C.2.2.2 Steam

Steam is assumed to enter and leave the heat exchanger as saturated vapour and saturated liquid, respectively. The amount of steam is calculated according to equation (9), where m_s is the steam mass flow rate, and h_e is the evaporation energy.

$$\dot{m}_s = \dot{Q} / h_e \tag{9}$$

Table B 8: Steam classes that are taken as utilities for this project, the absolute pressure, temperature and evaporation enthalpy

			Steam class		
			High Pressure (HP)	Medium Pressure (MP)	Low Pressure (LP)
p	[Bara]	:	55	11.5	3.6
T	[°C]	:	270	186	139.9
h_e	[kJ/kg]	:	1604.4	1991	2144
Costs Steam [30]	$/ton	:	19.01	17.57	16.81
Process temperature usage			Until 250°C	Until 166°C	Until 120°C

C.2.2.3 Fuel Oil No2

The amount of fuel oil is calculated according to the following equation:

$$\dot{m}_F = \dot{Q} / HHV \tag{10}$$

Table B 9: Characteristics of fuel oil No.2 that is taken as utility in this project for process temperatures until 300°C [23].

Fuel Oil No.2 ASTM standard D396	HHV [kJ/kg]	ρ [kg/m3]	Process temperature usage	Costs
	43046	900	Until 300°C	549 [$/m3] [23]

C.2.2.4 Dowtherm A

Dowtherm A is used in a vapor phase system. The amount is calculated according to the following equation:

$$\dot{m}_F = \dot{Q} / (h_v - h_l) \tag{11}$$

Table B 10: Characteristics of Dowtherm A that is taken as utility in this project for process temperatures until 350°C

Dowtherm A ASTM standard D396	h_v [kJ/kg]	h_L [kJ/kg] at 400°C	Process temperature usage	Costs [$/ton]
	1014.8	808.7	Until 350°C	Average: 2.86 [23,31,32]

C.3 Inventory Tables

C.3.1 Background System - Collection and Sorting of PA6 Waste

Sorting datasets are explained in more detail in Section B.2.1.

Table C 2: REFERENCE SWITCH ACTIVITY - Inventory table listing activities, locations, quantities, and units for 1 kg of collected and sorted PA6 waste, based on the dataset for sorting of PA6-containing derelict fishing gear, as reported by Schneider et al. (2023) [5], with reference status of a PA6 waste fraction of 0.5. This activity serves as a SWITCH ACTIVITY input in the chemical recycling processes, where it is varied as an alternative scenario to other sorting options and serves as the base case. Activities subject to variation in the sensitivity analysis and Monte Carlo uncertainty analysis are highlighted in italics as SWITCH ACTIVITIES.

Name	Activity	Location	Quantity	Unit
unsorted Nylon 6 (burden-free)	----burden free----	DE	1.000	kg
Electricity	*SWITCH ACTIVITY: market for electricity, medium voltage* as reference activity or *electricity production, wind, <1MW turbine, onshore*	DE	0.180	kWh
Steel Waste	aluminium, in mixed metal scrap, Recycled Content cut-off	GLO	-0.148	kg
Transport 32 ton	market for transport, freight, lorry >32 metric ton, EURO4	RER	0.840	ton km
Transport 16 ton	transport, freight, lorry 7.5-16 metric ton, EURO5	RER	0.800	ton km
Transport 16 ton	market for transport, freight, lorry 7.5-16 metric ton, EURO5	RER	0.200	ton km

Table C 3: SWITCH ACTIVITY - Inventory table showing the activities, locations, quantities and units for 1kg of collected and sorted PA6 waste accorting to the dataset of mixed plastic waste sorting described by of Haupt et al. (2018) [6], with reference status of a PA6 waste fraction of 0.5. This activity itself serves as a SWITCH ACTIVITY input in the chemical recycling processes, where it is varied as an alternative scenario to the other sorting options. Activities subject to variation in the sensitivity analysis and Monte Carlo uncertainty analysis are highlighted in italics as SWITCH ACTIVITIES.

Name	Activity	Location	Quantity	Unit
unsorted Nylon 6 (burden-free)	----burden free----	DE	1.000	kg
Waste facility	waste preparation facility construction	CH	4.00E-09	unit
Carbon steel	market for steel, low-alloyed	GLO	0.011	kg
Wire drawing	wire drawing, steel	RER	0.011	kg
Diesel	market for diesel, burned in building machine	GLO	0.160	kg

Electricity	*SWITCH ACTIVITY: market for electricity, medium voltage* as reference activity or *electricity production, wind, <1MW turbine, onshore*	DE	0.075	kWh
Heat	market for heat, central or small-scale, other than natural gas	Europe without Switzerland	0.066	MJ
Transport 32 ton	market for transport, freight, lorry >32 metric ton, EURO4	RER	1.680	ton km
Transport 16 ton	transport, freight, lorry 7.5-16 metric ton, EURO5	RER	1.600	ton km
Transport 16 ton	market for transport, freight, lorry 7.5-16 metric ton, EURO5	RER	0.400	ton km

C.3.2 Background System - Prepurification of PA6 Waste

Purification datasets are explained in more detail in Section B.2.2.

Table C 4: REFERENCE ACTIVITY - Inventory table showing the activities, locations, quantities and units for 1kg of collected, sorted, and purified PA6 by the dissolution process described by Costamagna et al. (2023) [7], with reference status of a PA6 waste fraction of 0.5 and a PA6 waste yield of 0.9. This activity itself serves as a SWITCH ACTIVITY input in the chemical recycling processes, where it is varied as an alternative scenario to the other purification options. Activities subject to variation in the sensitivity analysis and Monte Carlo uncertainty analysis are highlighted in italics as SWITCH ACTIVITIES.

**Name**	**Activity**	**Location**	**Quantity**	**Unit**
Sorted PA6 waste	Collected and sorted PA6 waste (see Section C.3.1)	DE	1.111	kg
Ethylene glycol	market for ethylene glycol	GLO	0.051	kg
Water	market for water, deionised	Europe without Switzerland	3.00E-03	kg
Steam	market for heat, from steam, in chemical industry	RER	0.008	MJ
Electricity	*SWITCH ACTIVITY: market for electricity, medium voltage* as reference activity or *electricity production, wind, <1MW turbine, onshore*	DE	0.400	kWh
Waste water	market for wastewater, average	Europe without Switzerland	-3.00E-06	m3
Emitted ethylene	Ethylene in categories air	-	1.11E-06	kg

Table C 5: SWITCH ACTIVITY - Inventory table listing activities, locations, quantities, and units for 1 kg of collected, sorted, and purified PA6 waste, based on the dataset for density separation and washing of PA6-containing derelict fishing gear, as reported by Schneider et al. (2023) [5], with reference status of a PA6 waste fraction of 0.5 and a PA6 waste yield of 0.9. This activity serves as a SWITCH ACTIVITY input in the chemical recycling processes, where it is varied as an alternative scenario to other purification options and serves as the base case. Activities subject to variation in the sensitivity analysis and Monte Carlo uncertainty analysis are highlighted in italics as SWITCH ACTIVITIES.

**Name**	**Activity**	**Location**	**Quantity**	**Unit**
Sorted PA6 waste	Collected and sorted PA6 waste (see Section C.3.1)	DE	1.111	kg
Salt	sodium chloride production, powder	RER	0.188	kg
Water	market for water, deionised	Europe without Switzerland	5.111	kg
PolySepar CFX 1088	market for aluminium hydroxide	GLO	0.005	kg
PolySepar PK 1455	market for cationic resin	RER	0.000	kg
Electricity	*SWITCH ACTIVITY: market for electricity, medium voltage* as reference activity or *electricity production, wind, <1MW turbine, onshore* as switch	DE	0.374	kWh
Waste Water	market for wastewater, average	Europe without Switzerland	-0.005	m3
Inert Waste	market for inert waste	RoW	-1.120	kg
Plastic Waste	waste polyethylene, for recycling, unsorted, Recycled Content cut-off	GLO	-0.051	kg

C.3.3 Foreground System - Chemical Recycling of Collected, Sorted and Pre-Purified PA6 Waste

C.3.3.1 H_3PO_4 Process

Table C 6: Inventory table showing the activities, locations, quantities and units for the production of 1kg CL by depolymerization of PA6 using H_3PO_4. The shown quantities are based on the reference calculation but altered within the sensitivity analysis according to changes of continuous parameters given in Section B.1. Activities subject to variation in the sensitivity analysis and Monte Carlo uncertainty analysis are highlighted in italics as SWITCH ACTIVITIES.

**Category**	**Name**	**Activity**	**Location**	**Quantity**	**Unit**
Raw materials	H_3PO_4	market for phosphoric acid, fertiliser grade, without water, in 70% solution state	RER	0.630	kg

	Collected and sorted PA6 waste	*SWITCH ACTIVITY: Collected and sorted PA6 waste* (see Section C.3.1) as reference activity or *collected, sorted, and purified PA6 by the dissolution* (see Section B.2.1)	DE	1.285	kg
	Water	market for water, deionised	Europe without Switzerland	0.006	kg
Utilities	Steam	market for heat, from steam, in chemical industry	RER	5.771	MJ
	Electricity	*SWITCH ACTIVITY: market for electricity, medium voltage* as reference activity or *electricity production, wind, <1MW turbine, onshore*	DE	0.195	kWh
	Light fuel oil	heat production, light fuel oil, at boiler 100kW, non-modulating	Europe without Switzerland	2.820	MJ
	Cooling water	Water, cooling, unspecified natural origin	-	0.078	m³
Waste	Solid waste	*SWITCH ACTIVITY: treatment of hazardous waste, hazardous waste incineration* as reference activity or *treatment of waste plastic, mixture, municipal incineration*	Europe without Switzerland	0.834	kg
Process equipment	Stainless steel	market for steel, chromium steel 18/8, hot rolled	GLO	2.75E-05	kg
	Carbon steel	market for steel, low-alloyed, hot rolled	GLO	1.07E-05	kg
	Concrete	market group for concrete, normal strength	GLO	2.40E-08	m³
	Powering	market for cable, unspecified	GLO	2.19E-07	kg
	Aluminium	market for sheet rolling, aluminium	GLO	7.26E-06	kg
	Glass wool	market for glass wool mat	GLO	3.78E-06	kg
	Furnace	industrial furnace production, 1MW, oil	CH	1.22E-09	unit
	Pump	market for water pump, 22kW	GLO	1.13E-08	unit
	Conveyor belt	conveyor belt production	RER	2.03E-08	m³
chemical factory	Stainless steel	market for steel, chromium steel 18/8, hot rolled	GLO	-1.92E-05	kg
	Carbon steel	market for steel, low-alloyed, hot rolled	GLO	-3.26E-05	kg
	Concrete	market for concrete, normal strength	CH	-5.77E-09	m³
	Concrete	market for concrete, normal strength	AT	-3.17E-09	m³
	Chemical factory	chemical factory construction, organics	RER	9.61E-12	unit
transportatio n	Transport 16 ton	transport, freight, lorry 7.5-16 metric ton, EURO5	RER	1.62E-05	ton km
	Transport 16 ton	market for transport, freight, lorry 7.5-16 metric ton, EURO5	RER	6.49E-05	ton km

	Transport 32 ton	market for transport, freight, lorry >32 metric ton, EURO5	RER	3.20E-05	ton km
Emissions	Caprolactam	Cyclohexane in category Air	-	0.007	kg

C.3.3.2 HTW Process

Table C 7: Inventory table showing the activities, locations, quantities and units for the production of 1kg CL by depolymerization of PA6 using HTW. The shown quantities are based on the reference calculation but altered within the sensitivity analysis according to changes of continuous parameters given in Section B.1. Activities subject to variation in the sensitivity analysis and Monte Carlo uncertainty analysis are highlighted in italics as SWITCH ACTIVITIES.

Category	Name	Activity	Location	Quantity	Unit
Raw materials	Collected and sorted PA6 waste	*SWITCH ACTIVITY: Collected and sorted PA6 waste* (see Section C.3.1) as reference activity or *collected, sorted, and purified PA6 by the dissolution* (see Section B.2.1)	DE	1.207	kg
	Water	market for water, deionised	Europe without Switzerland	0.107	kg
	$CaOH_2$	market for lime, hydrated, packed	RER	0.004	kg
Utilities	Steam	market for heat, from steam, in chemical industry	RER	68.201	MJ
	Electricity	*SWITCH ACTIVITY: market for electricity, medium voltage* as reference activity or *electricity production, wind, <1MW turbine, onshore*	DE	0.516	kWh
	Light fuel oil	heat production, light fuel oil, at boiler 100kW, non-modulating	Europe without Switzerland	35.289	MJ
	Cooling water	Water, cooling, unspecified natural origin	-	1.308	m³
Waste	Solid waste	*SWITCH ACTIVITY: treatment of waste polyethylene terephthalate, municipal incineration* as reference activity or *treatment of waste polyethylene, municipal incineration*	CH	0.187	kg
Process equipment	Stainless steel	market for steel, chromium steel 18/8, hot rolled	GLO	1.61E-02	kg
	Carbon steel	market for steel, low-alloyed, hot rolled	GLO	2.74E-05	kg
	Concrete	market group for concrete, normal strength	GLO	1.21E-07	m³
	Powering	market for cable, unspecified	GLO	1.10E-06	kg

	Aluminium	market for sheet rolling, aluminium	GLO	2.42E-05	kg
	Glass wool	market for glass wool mat	GLO	1.75E-05	kg
	Furnace	industrial furnace production, 1MW, oil	CH	7.89E-08	unit
	Pump	market for water pump, 22kW	GLO	2.49E-06	unit
	Conveyor belt	conveyor belt production	RER	2.03E-08	m³
chemical factory	Stainless steel	market for steel, chromium steel 18/8, hot rolled	GLO	-6.08E-03	kg
	Carbon steel	market for steel, low-alloyed, hot rolled	GLO	-1.03E-02	kg
	Concrete	market for concrete, normal strength	CH	-1.83E-06	m³
	Concrete	market for concrete, normal strength	AT	-1.00E-06	m³
	Chemical factory	chemical factory construction, organics	RER	3.04E-09	unit
transportation	Transport 16 ton	transport, freight, lorry 7.5-16 metric ton, EURO5	RER	4.74E-05	ton km
	Transport 16 ton	market for transport, freight, lorry 7.5-16 metric ton, EURO5	RER	1.90E-04	ton km
	Transport 32 ton	market for transport, freight, lorry >32 metric ton, EURO5	RER	1.62E-02	ton km
Emissions	Caprolactam	Cyclohexane in category Air	-	0.017	kg

C.3.3.3 iPrOH Process

Table C 8: Inventory table showing the activities, locations, quantities and units for the production of 1kg CL by depolymerization of PA6 using iPrOH. The shown quantities are based on the reference calculation but altered within the sensitivity analysis according to changes of continuous parameters given in Section B.1. Activities subject to variation in the sensitivity analysis and Monte Carlo uncertainty analysis are highlighted in italics as SWITCH ACTIVITIES.

**Category**	**Name**	**Activity**	**Location**	**Quantity**	**Unit**
Raw materials	Collected and sorted PA6 waste	*SWITCH ACTIVITY: Collected and sorted PA6 waste* (see Section C.3.1) as reference activity or *collected, sorted, and purified PA6 by the dissolution* as switch activity within sensitivity analysis (see Section B.2.1)	DE	1.212	kg
	iPrOH	market for isopropanol	RER	0.234	kg
Utilities	Steam	market for heat, from steam, in chemical industry	RER	10.157	MJ
	Electricity	*SWITCH ACTIVITY: market for electricity, medium voltage* as reference activity or *electricity production, wind, <1MW turbine, onshore* as switch activity within sensitivity analysis	DE	0.491	kWh
	Light fuel oil	heat production, light fuel oil, at boiler 100kW, non-modulating	Europe without Switzerland	6.142	MJ
	Cooling water	Water, cooling, unspecified natural origin	-	0.211	m³
Waste	Solid waste	*SWITCH ACTIVITY: treatment of hazardous waste, hazardous waste incineration* as reference activity or *treatment of waste plastic, mixture, municipal incineration* as switch activity within sensitivity analysis	Europe without Switzerland	0.330	kg
Process equipment	Stainless steel	market for steel, chromium steel 18/8, hot rolled	GLO	1.56E-02	kg
	Carbon steel	market for steel, low-alloyed, hot rolled	GLO	1.54E-05	kg
	Concrete	market group for concrete, normal strength	GLO	1.69E-07	m³
	Powering	market for cable, unspecified	GLO	1.54E-06	kg
	Aluminium	market for sheet rolling, aluminium	GLO	3.36E-05	kg
	Glass wool	market for glass wool mat	GLO	2.24E-05	kg
	Furnace	industrial furnace production, 1MW, oil	CH	1.11E-08	unit
	Pump	market for water pump, 22kW	GLO	2.40E-06	unit
	Conveyor belt	conveyor belt production	RER	2.03E-08	m³
chemical factory	Stainless steel	market for steel, chromium steel 18/8, hot rolled	GLO	-5.90E-03	kg

	Carbon steel	market for steel, low-alloyed, hot rolled	GLO	-1.00E-02	kg
	Concrete	market for concrete, normal strength	CH	-1.77E-06	m³
transportation	Transport 16 ton	transport, freight, lorry 7.5-16 metric ton, EURO5	RER	3.31E-05	ton km
	Transport 16 ton	market for transport, freight, lorry 7.5-16 metric ton, EURO5	RER	1.32E-04	ton km
	Transport 32 ton	market for transport, freight, lorry >32 metric ton, EURO5	RER	1.59E-02	ton km
Emissions	Caprolactam	Cyclohexane in category Air	-	0.001	kg
	Isopropanol	2-Propanol	-	0.128	kg

C.3.3.4 NaOH Process

Table C 9: Inventory table showing the activities, locations, quantities and units for the production of 1kg CL by depolymerization of PA6 using NaOH. The shown quantities are based on the reference calculation but altered within the sensitivity analysis according to changes of continuous parameters given in Section B.1. Activities subject to variation in the sensitivity analysis and Monte Carlo uncertainty analysis are highlighted in italics as SWITCH ACTIVITIES.

Category	Name	Activity	Location	Quantity	Unit
Raw materials	Sorted PA6 waste	*SWITCH ACTIVITY: Collected and sorted PA6 waste* (see Section C.3.1) as reference activity or *collected, sorted, and purified PA6 by the dissolution* (see Section B.2.1)	DE	1.106	kg
	NaOH	market for neutralising agent, sodium hydroxide-equivalent	GLO	0.011	kg
Utilities	Steam	market for heat, from steam, in chemical industry	RER	0.682	MJ
	Electricity	*SWITCH ACTIVITY: market for electricity, medium voltage* as reference activity or *electricity production, wind, <1MW turbine, onshore*	DE	0.384	kWh
	Light fuel oil	heat production, light fuel oil, at boiler 100kW, non-modulating	Europe without Switzerland	0.731	MJ
	Cooling water	Water, cooling, unspecified natural origin	-	0.014	m³

Waste	Solid waste	treatment of hazardous waste, hazardous waste incineration	CH	0.105	kg
Process equipment	Stainless steel	market for steel, chromium steel 18/8, hot rolled	GLO	1.49E-05	kg
	Carbon steel	market for steel, low-alloyed, hot rolled	GLO	2.37E-06	kg
	Concrete	market group for concrete, normal strength	GLO	3.15E-09	m³
	Powering	market for cable, unspecified	GLO	2.87E-08	kg
	Aluminium	market for sheet rolling, aluminium	GLO	7.61E-07	kg
	Glass wool	market for glass wool mat	GLO	3.25E-07	kg
	Pump	market for water pump, 22kW	GLO	5.56E-09	unit
	Conveyor belt	conveyor belt production	RER	1.02E-08	m³
chemical factory	Stainless steel	market for steel, chromium steel 18/8, hot rolled	GLO	-8.81E-06	kg
	Carbon steel	market for steel, low-alloyed, hot rolled	GLO	-1.50E-05	kg
	Concrete	market for concrete, normal strength	CH	-2.65E-09	m³
transportation	Transport 16 ton	transport, freight, lorry 7.5-16 metric ton, EURO5	RER	3.50E-06	ton km
	Transport 16 ton	market for transport, freight, lorry 7.5-16 metric ton, EURO5	RER	1.40E-05	ton km
	Transport 32 ton	market for transport, freight, lorry >32 metric ton, EURO5	RER	1.44E-05	ton km
Emissions	Caprolactam	Cyclohexane in category Air	-	0.001	kg

D Results and Interpretations

D.1 Environmental Impacts

Table D 1: Environmental impacts (reference case) for the four chemical recycling pathways for Nylon 6 to caprolactam, shown across all impact categories.

	GWP [kg CO_2-eq]	FET [CTUe]	EP [kg P-eq]	HTC [CTUh]	HTNC [CTUh]	IR [kBq U235-eq]	LU [-]	ADP [kg Sb-eq]	WDP [m^3 world-eq deprived]
H_3PO_4	4.46	42.36	1.49E-03	2.01E-09	2.85E-08	0.24	27.65	1.34E-05	1.86
HTW	13.04	48.74	2.28E-03	4.22E-09	9.02E-08	0.45	21.73	6.93E-05	0.76
iPrOH	4.42	18.66	1.52E-03	2.40E-09	6.30E-08	0.27	13.84	6.53E-05	0.58
NaOH	1.46	9.77	8.70E-04	5.30E-10	1.04E-08	0.14	5.18	3.99E-06	0.23

Table D 2: Environmental impacts (lower bound) for the four chemical recycling pathways for Nylon 6 to caprolactam, shown across all impact categories.

	GWP [kg CO_2-eq]	FET [CTUe]	EP [kg P-eq]	HTC [CTUh]	HTNC [CTUh]	IR [kBq U235-eq]	LU [-]	ADP [kg Sb-eq]	WDP [m^3 world-eq deprived]
H_3PO_4	3.86	40.52	9.50E-04	1.90E-09	2.40E-08	0.16	26.54	1.19E-05	1.73
HTW	8.12	44.92	1.18E-03	3.41E-09	7.03E-08	0.29	18.07	5.49E-05	0.51
iPrOH	3.74	15.68	9.98E-04	2.10E-09	5.00E-08	0.17	12.08	5.19E-05	0.42
NaOH	0.97	8.44	3.45E-04	4.90E-10	7.18E-09	0.05	4.49	2.80E-06	0.11

Table D 3: Environmental impacts (upper bound) for the four chemical recycling pathways for Nylon 6 to caprolactam, shown across all impact categories.

	GWP [kg CO_2-eq]	FET [CTUe]	EP [kg P-eq]	HTC [CTUh]	HTNC [CTUh]	IR [kBq U235-eq]	LU [-]	ADP [kg Sb-eq]	WDP [m^3 world-eq deprived]
H_3PO_4	4.54	44.15	1.52E-03	2.61E-09	3.24E-08	0.24	32.90	2.08E-05	1.89
HTW	13.30	50.90	2.31E-03	5.15E-09	1.08E-07	0.45	27.13	9.62E-05	0.81
iPrOH	4.75	21.37	1.58E-03	3.31E-09	8.06E-08	0.28	19.57	9.19E-05	0.63
NaOH	2.01	12.31	9.51E-04	1.15E-09	1.51E-08	0.16	10.17	1.06E-05	0.27

D.2 Impacts for different LCIA Methods: EF 3.1, ReCiPe 2016, TRACI 2.1

Table D 4 shows midpoint LCIA results for the four PA6 to CL routes across EF 3.1, ReCiPe 2016 (H), and TRACI 2.1. Across EF 3.1, ReCiPe 2016 midpoint, and TRACI 2.1, the qualitative ranking of routes is stable: NaOH consistently shows the lowest impacts, HTW mostly the highest, and H_3PO_4 and iPrOH fall in between, occasionally swapping places by category. Notably, for all categories where H_3PO_4 has the highest impacts under EF 3.1, it also shows the highest relative impacts among processes when evaluated with ReCiPe.

While relative rankings are generally robust, absolute magnitudes are not cross-comparable unless the functional unit, system boundary, background database, and the same LCIA method and version are used. This follows LCA best practice: use LCIA primarily for relative comparisons within one harmonized setup, not for cross-method magnitude claims [33]. Related categories differ across methods, including their units. For example, freshwater ecotoxicity in EF 3.1 and TRACI 2.1 is reported in CTUe (comparative toxic units for ecosystems), whereas ReCiPe expresses it in kg 1,4-DCB-eq. Consequently, for these midpoint indicators, the absolute values are potentials, not direct predictions of ecological damage. They are intended to support relative comparisons within a consistent methodological frame.

Table D 4: Midpoint LCIA results for the four PA6 to CL routes across EF 3.1, ReCiPe 2016 (H), and TRACI 2.1. For each EF 3.1 category, the most closely corresponding ReCiPe/TRACI midpoint category is reported. If no direct counterpart exists, the cell is left blank. Values are per functional unit under a harmonized system boundary while units may differ by method.

**EF 3.1**									
	Climate change [kg CO_2-Eq]	Ecotoxicity: freshwater [CTUe]	Eutrophication: freshwater [kg P-Eq]	Human toxicity: carcinogenic [CTUh]	Human toxicity: non-carcinogenic [CTUh]	Ionising radiation: human health [kBq U235-Eq]	Land use [dimensionless]	Material resources: metals/minerals [kg Sb-Eq]	Water use [m^3 world eq. deprived]
Acid	4.5	42.4	1.5E-03	2.0E-09	2.8E-08	0.2	27.6	1.34E-05	1.9
HTW	13.0	48.7	2.3E-03	4.2E-09	9.0E-08	0.4	21.7	6.93E-05	0.8
iPrOH	4.4	18.7	1.5E-03	2.4E-09	6.3E-08	0.3	13.8	6.53E-05	0.6
NaOH	1.5	9.8	8.7E-04	5.3E-10	1.0E-08	0.1	5.2	3.99E-06	0.2
**ReCiPe 2016 v1.03, midpoint (H)**									
	Climate change [kg CO_2-Eq]	Ecotoxicity: freshwater [kg 1,4-DCB-Eq]	Eutrophication: freshwater [kg P-Eq]	Human toxicity: carcinogenic [kg 1,4-DCB-Eq]	Human toxicity: non-carcinogenic [kg 1,4-DCB-Eq]	Ionising radiation [kBq Co-60-Eq]	Land use [$m^2 \cdot a$ crop-Eq]	Mineral resource scarcity [kg Cu-Eq]	Water consumption [m^3]

Acid	4.5	1.3E-02	1.7E-04	7.6E-02	1.0	1.5E-02	0.17	0.08	4.2E-02
HTW	13.3	1.2E-02	2.5E-04	1.2E-01	2.2	2.8E-02	0.14	0.10	1.8E-02
iPrOH	4.5	1.1E-02	1.5E-04	9.2E-02	1.7	1.7E-02	0.09	0.06	1.4E-02
NaOH	1.5	3.0E-03	9.0E-05	1.8E-02	0.3	8.8E-03	0.03	0.02	5.3E-03
**TRACI v2.1**									
	Climate change [kg CO_2-Eq]	Ecotoxicity: freshwater [CTUe]	Eutrophication [kg N-Eq]	Human toxicity: carcinogenic [CTUh]	Human toxicity: non-carcinogenic [CTUh]				
Acid	4.4	5.4	3.9E-03	1.0E-07	5.5E-07				
HTW	12.9	7.6	3.1E-03	1.4E-07	9.5E-07				
iPrOH	4.4	7.2	2.5E-03	1.0E-07	6.4E-07				
NaOH	1.4	1.6	8.4E-04	2.4E-08	1.3E-07				

D.3 Probability Distributions

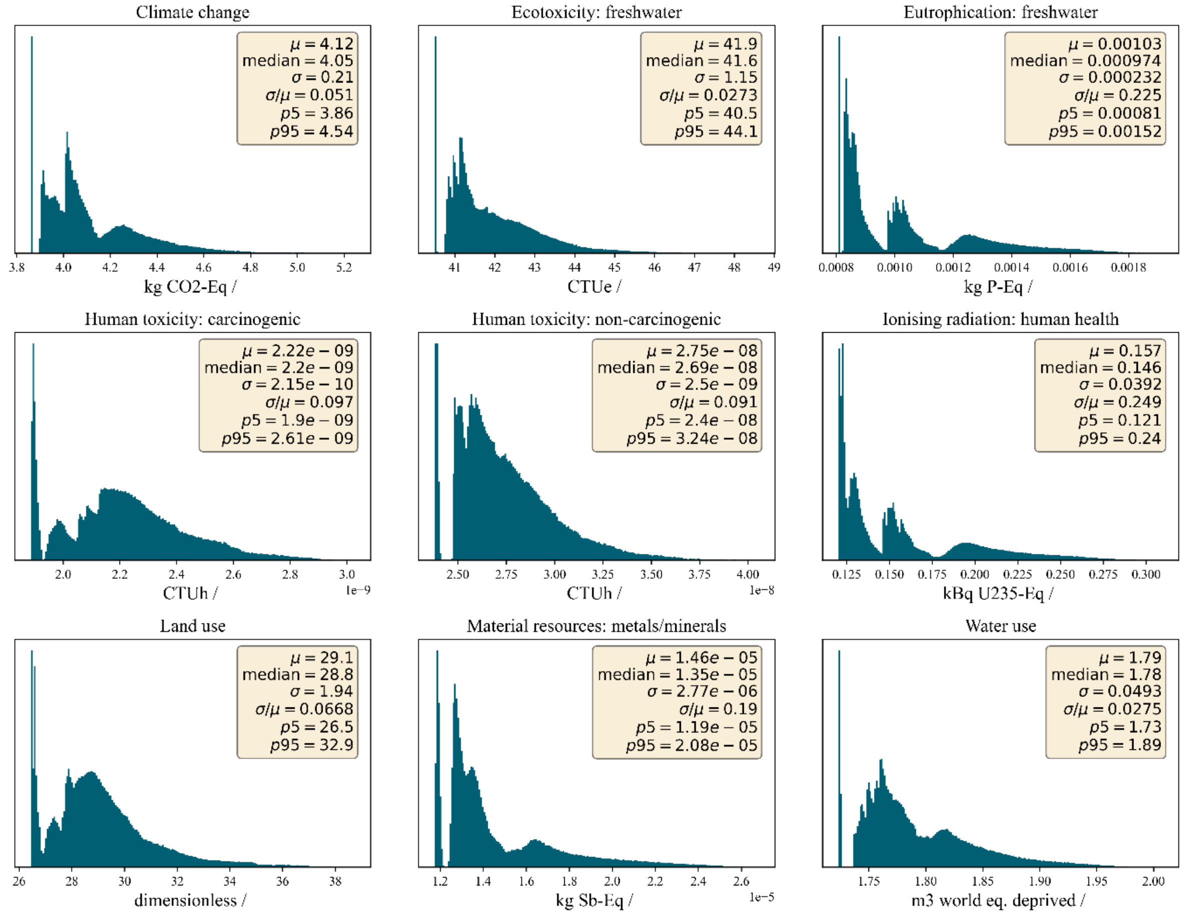

Figure D 1: Distributions of impact category results for the simulated process, showing mean (μ), median, standard deviation (σ), coefficient of variation (σ/μ), and 5th (p5) and 95th (p95) percentiles across climate change, ecotoxicity, eutrophication, human toxicity (carcinogenic and non-carcinogenic), ionizing radiation, land use, material resource depletion, and water use indicators for the depolymerization of PA6 using H_3PO_4 based on varying the following parameters outlined in section B.1: operating years, electricity source, PA6 waste fraction, PA6 sorting dataset, prepurification inclusion or exclusion, PA6 prepurification dataset, PA6 prepurification yield.

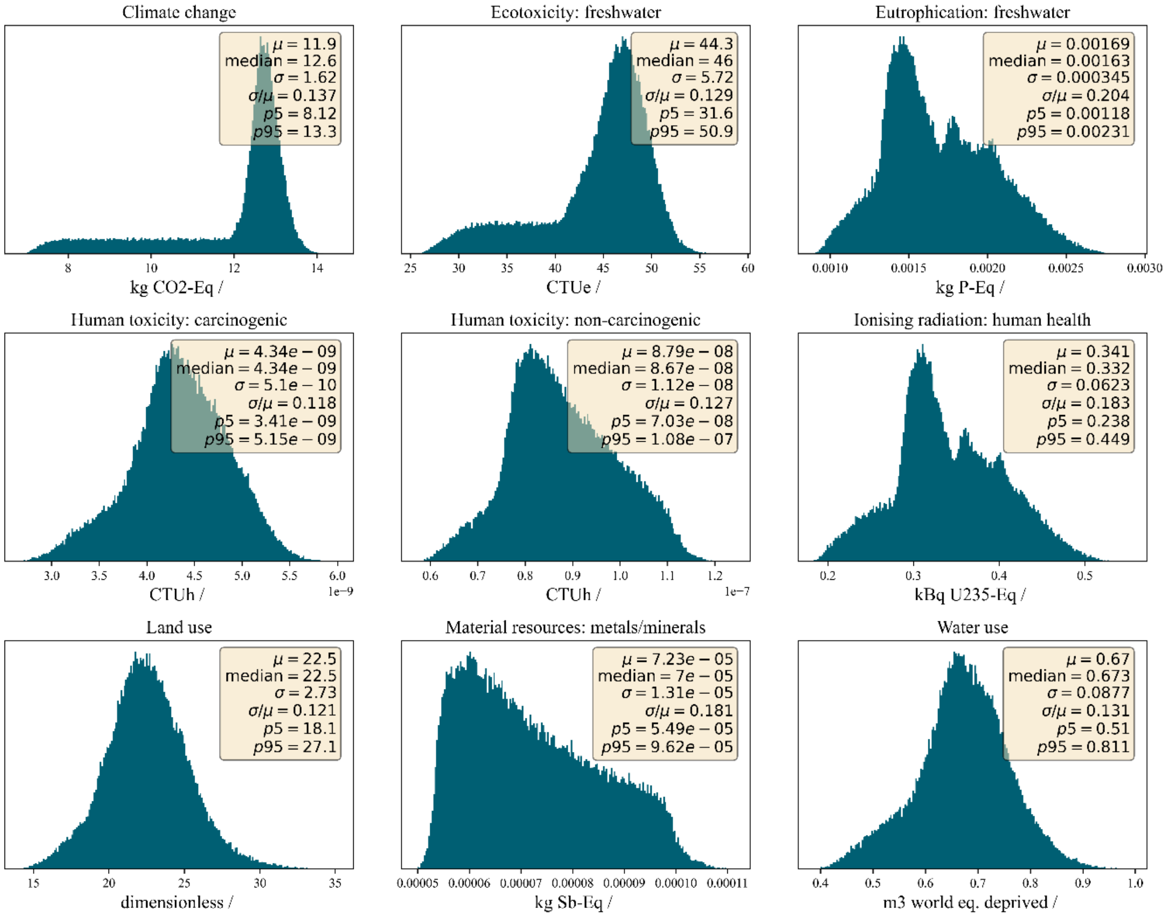

Figure D 2: Distributions of impact category results for the simulated process, showing mean (μ), median, standard deviation (σ), coefficient of variation (σ/μ), and 5th (p5) and 95th (p95) percentiles across climate change, ecotoxicity, eutrophication, human toxicity (carcinogenic and non-carcinogenic), ionizing radiation, land use, material resource depletion, and water use indicators for the depolymerization of PA6 using HTW based on varying the following parameters outlined in section C.4: operating years, electricity source, PA6 waste fraction, PA6 sorting dataset, prepurification inclusion or exclusion, PA6 prepurification dataset, PA6 prepurification yield, water amount, temperature, conversion, waste treatment dataset.

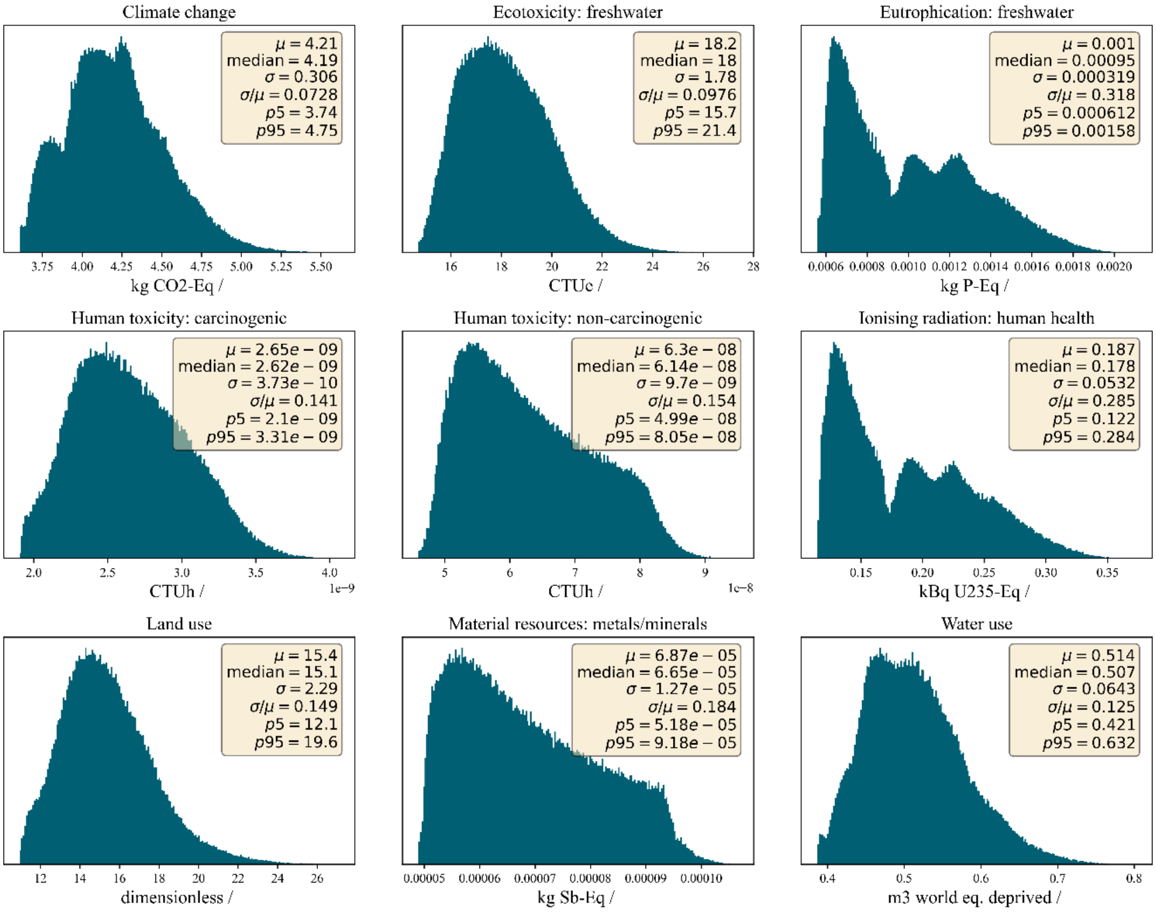

Figure D 3 Distributions of impact category results for the simulated process, showing mean (μ), median, standard deviation (σ), coefficient of variation (σ/μ), and 5th (p5) and 95th (p95) percentiles across climate change, ecotoxicity, eutrophication, human toxicity (carcinogenic and non-carcinogenic), ionizing radiation, land use, material resource depletion, and water use indicators for the depolymerization of PA6 using iPrOH based on varying the following parameters outlined in section B.1: operating years, electricity source, PA6 waste fraction, PA6 sorting dataset, prepurification inclusion or exclusion, PA6 prepurification dataset, PA6 prepurification yield, waste treatment dataset.

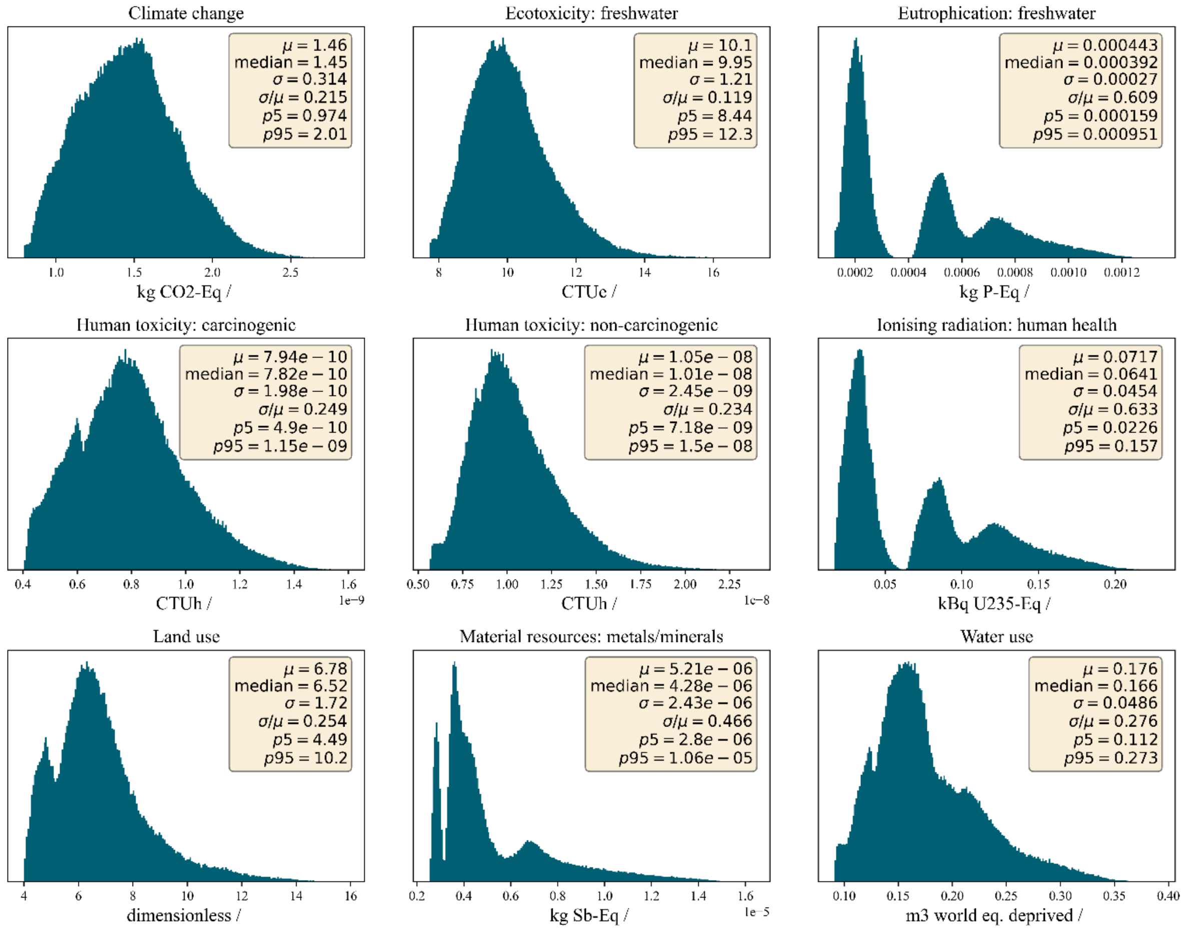

Figure D 4: Distributions of impact category results for the simulated process, showing mean (μ), median, standard deviation (σ), coefficient of variation (σ/μ), and 5th (p5) and 95th (p95) percentiles across climate change, ecotoxicity, eutrophication, human toxicity (carcinogenic and non-carcinogenic), ionizing radiation, land use, material resource depletion, and water use indicators for the depolymerization of PA6 using NaOH based on varying the following parameters outlined in section B.1: operating years, electricity source, PA6 waste fraction, PA6 sorting dataset, prepurification inclusion or exclusion, PA6 prepurification dataset, PA6 prepurification yield.

D.4 Violin Charts

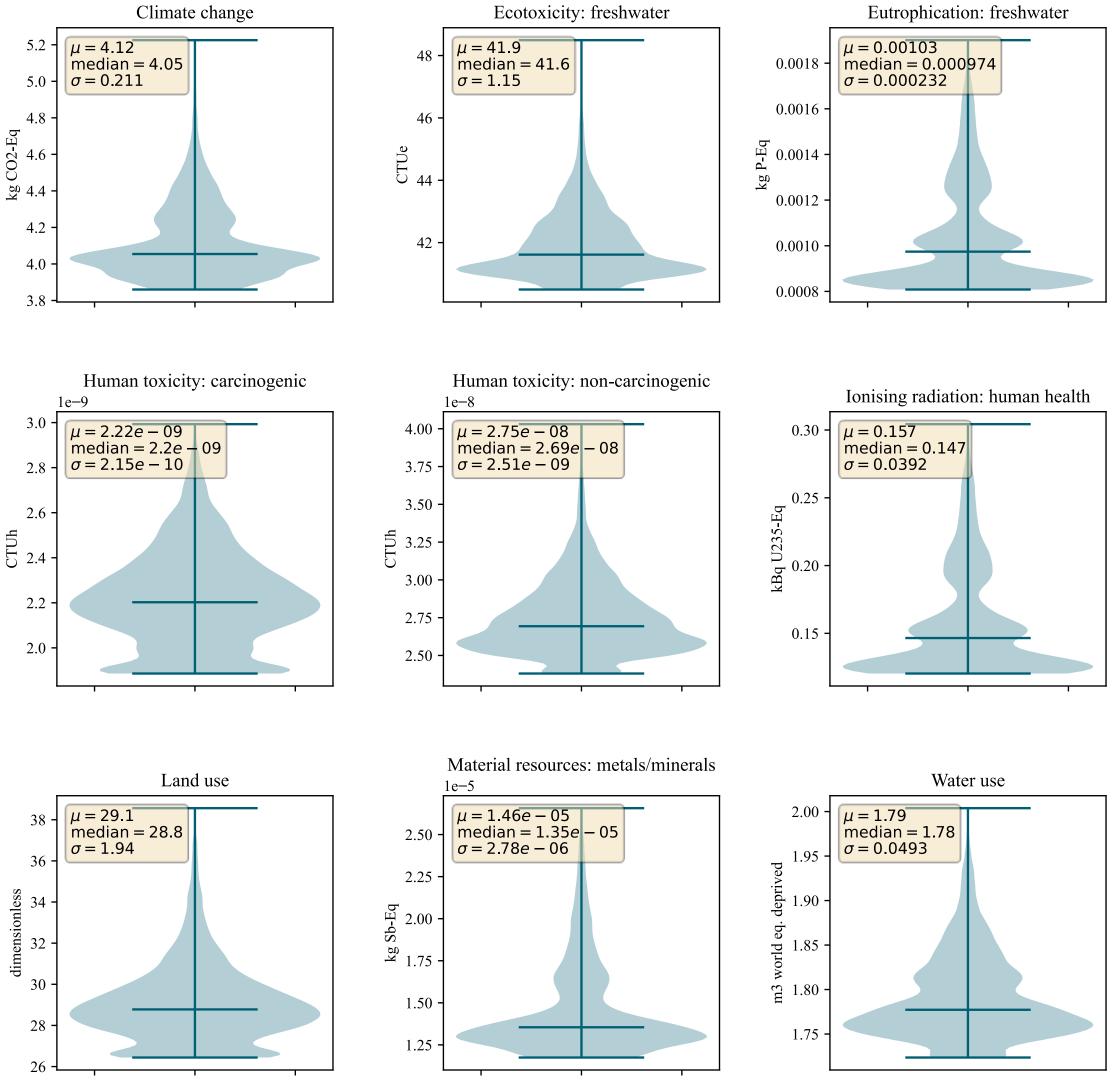

Figure D 5: Violin plot of impact category results for the simulated process, showing mean (μ), median, standard deviation (σ), coefficient of variation (σ/μ), and 5th (p5) and 95th (p95) percentiles across climate change, ecotoxicity, eutrophication, human toxicity (carcinogenic and non-carcinogenic), ionizing radiation, land use, material resource depletion, and water use indicators for the depolymerization of PA6 using H_3PO_4 based on varying the following parameters outlined in section B.1: operating years, electricity source, PA6 waste fraction, PA6 sorting dataset, prepurification inclusion or exclusion, PA6 prepurification dataset, PA6 prepurification yield.

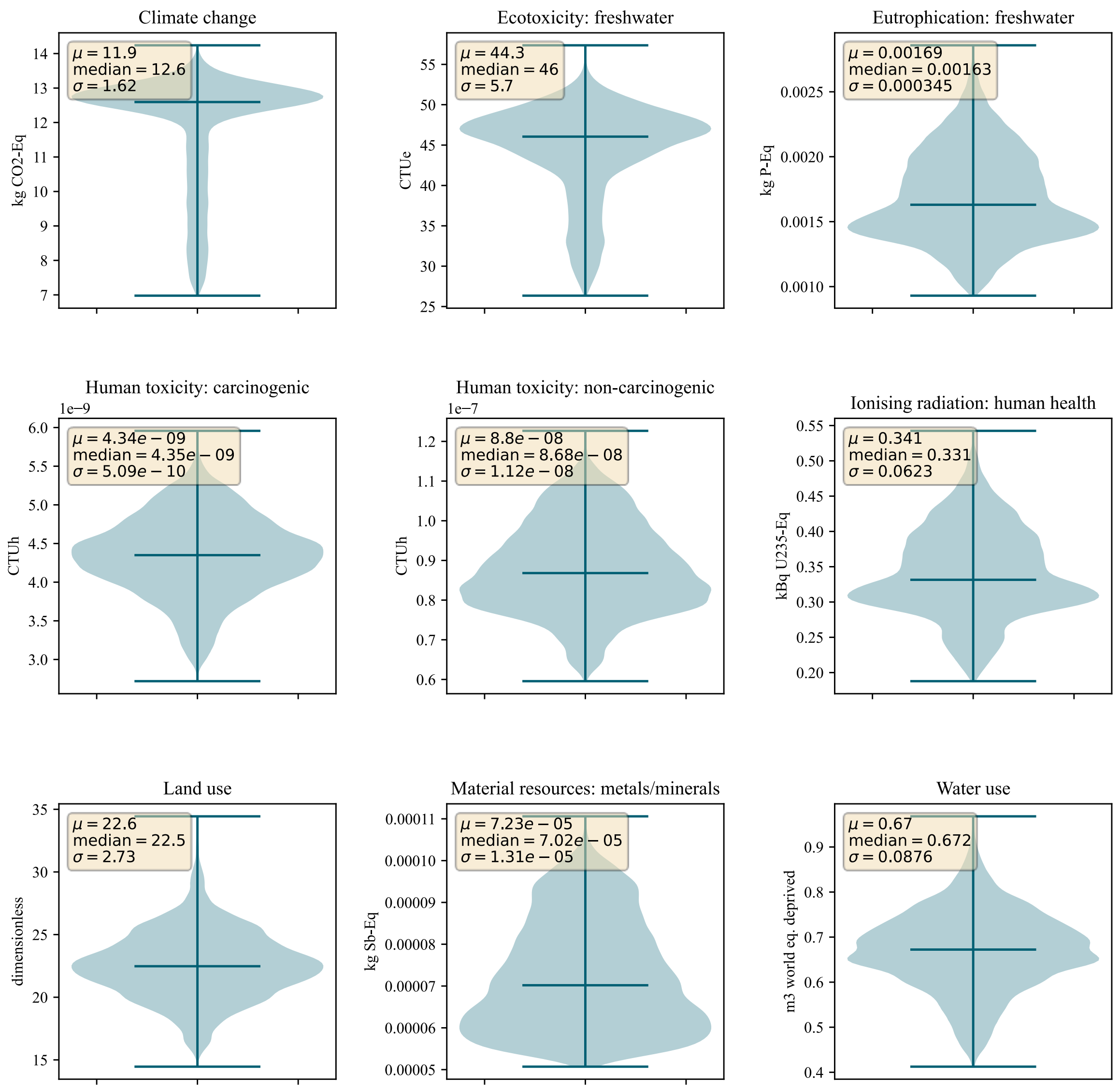

Figure D 6: Violin plot of impact category results for the simulated process, showing mean (μ), median, standard deviation (σ), coefficient of variation (σ/μ), and 5th (p5) and 95th (p95) percentiles across climate change, ecotoxicity, eutrophication, human toxicity (carcinogenic and non-carcinogenic), ionizing radiation, land use, material resource depletion, and water use indicators for the depolymerization of PA6 using HTW based on varying the following parameters outlined in section C.4: operating years, electricity source, PA6 waste fraction, PA6 sorting dataset, prepurification inclusion or exclusion, PA6 prepurification dataset, PA6 prepurification yield, water amount, temperature, conversion, waste treatment dataset.

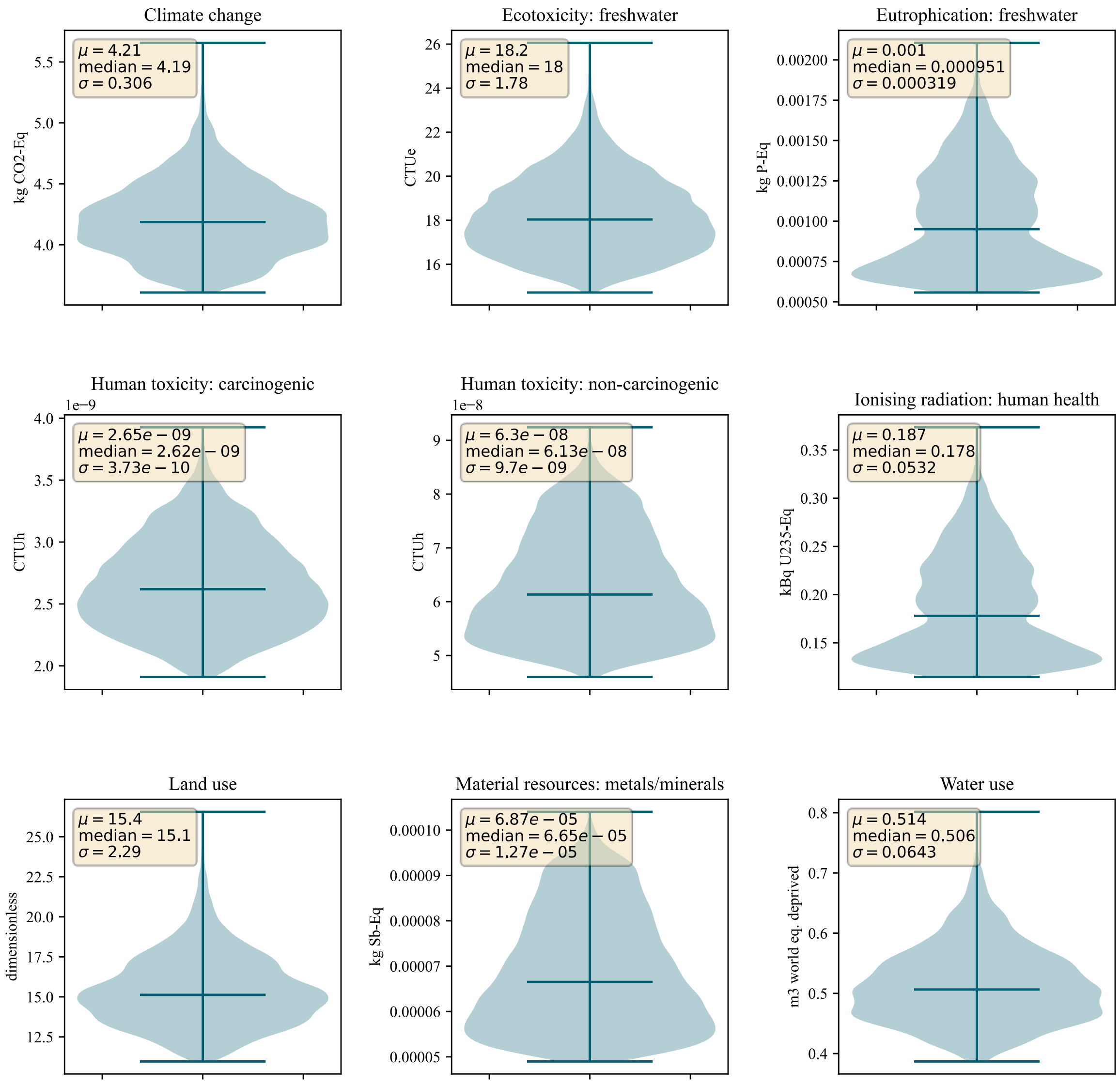

Figure D 7: Violin plot of impact category results for the simulated process, showing mean (μ), median, standard deviation (σ), coefficient of variation (σ/μ), and 5th (p5) and 95th (p95) percentiles across climate change, ecotoxicity, eutrophication, human toxicity (carcinogenic and non-carcinogenic), ionizing radiation, land use, material resource depletion, and water use indicators for the depolymerization of PA6 using iPrOH based on varying the following parameters outlined in section B.1: operating years, electricity source, PA6 waste fraction, PA6 sorting dataset, prepurification inclusion or exclusion, PA6 prepurification dataset, PA6 prepurification yield, waste treatment dataset.

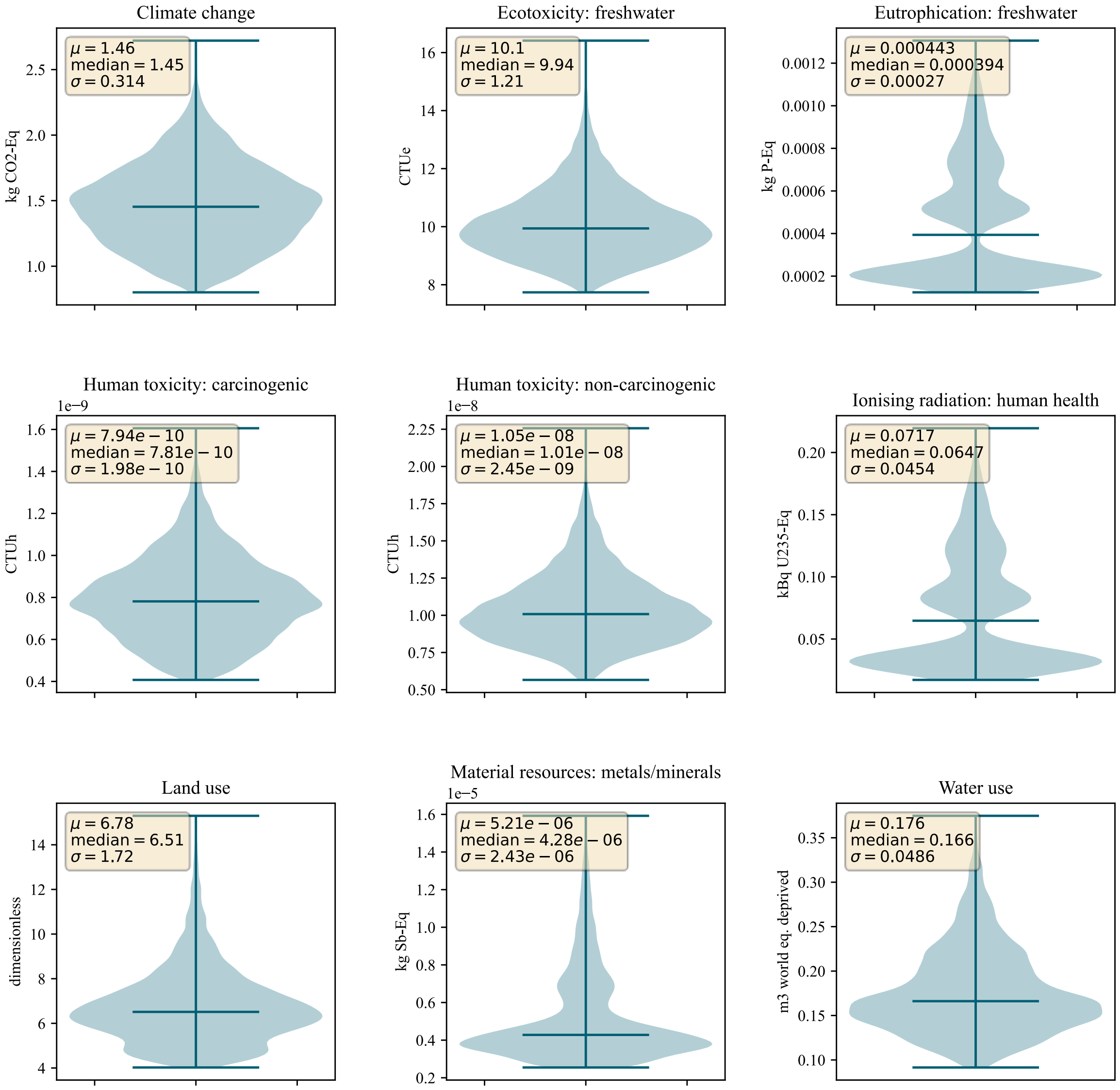

Figure D 8: Violin plot of impact category results for the simulated process, showing mean (μ), median, standard deviation (σ), coefficient of variation (σ/μ), and 5th (p5) and 95th (p95) percentiles across climate change, ecotoxicity, eutrophication, human toxicity (carcinogenic and non-carcinogenic), ionizing radiation, land use, material resource depletion, and water use indicators for the depolymerization of PA6 using NaOH based on varying the following parameters outlined in section B.1: operating years, electricity source, PA6 waste fraction, PA6 sorting dataset, prepurification inclusion or exclusion, PA6 prepurification dataset, PA6 prepurification yield.

D.5 Surrogate GWP Equations as Functions of Key Parameters

This section presents the surrogate equations used to estimate GWP for each chemical recycling process investigated. Each equation expresses GWP as a function of key process parameters. These surrogate models enable rapid scenario analysis and comparison between processes, as they allow for direct calculation of GWP without rerunning the full process simulation.

D.5.1 H_3PO_4 Process

Reference setting: CF = 0.9, years = 15, amt_{H3PO4} = 0.26638 kg/s, $PA6_{wastefrac}$ = 0.5, $PA6_{purifyield}$ = 0.990

GWP calculated using average German electricity, with PA6 input waste sorting and PA6 prepurification (dissolution) and hazardous waste treatment of process waste (reference: 4.46 kg CO_2 Eq.):

$$GWP[kgCO_2Eq.] = \frac{CF \cdot PA6_{purifyield} \cdot PA6_{wastefrac} \cdot years \cdot (7.14737 \cdot \dot{m}_{H3PO4} + 1.3424) + CF \cdot years \cdot (0.00114936 \cdot \dot{m}_{H3PO4} + 1.28481) \cdot \left(0.234696 \cdot PA6_{purifyield} \cdot PA6_{wastefrac} + 0.0866844 \cdot PA6_{wastefrac} + 0.174502\right)}{CF \cdot PA6_{purifyield} \cdot PA6_{wastefrac} \cdot years} + \frac{PA6_{purifyield} \cdot PA6_{wastefrac} \cdot (0.101258 \cdot CF \cdot years \cdot (0.0620547 \cdot \dot{m}_{H3PO4} + 0.50124) + 6.65266 \cdot 10^{-5} \cdot CF \cdot years \cdot (4624.11 \cdot \dot{m}_{H3PO4} + 2272.58) + 0.0187913)}{CF \cdot PA6_{purifyield} \cdot PA6_{wastefrac} \cdot years}$$

GWP calculated using wind electricity, with PA6 input waste sorting and PA6 prepurification (dissolution) and hazardous waste treatment of process waste (reference: 4.020 kg CO_2 Eq.):

$$GWP[kgCO_2Eq.] = \frac{CF \cdot PA6_{purifyield} \cdot PA6_{wastefrac} \cdot years \cdot (6.88124 \cdot \dot{m}_{H3PO4} + 1.32515) + CF \cdot years \cdot (0.00114936 \cdot \dot{m}_{H3PO4} + 1.28481) \cdot \left(0.234696 \cdot PA6_{purifyield} \cdot PA6_{wastefrac} + 0.0866844 \cdot PA6_{wastefrac} + 0.0523653\right)}{CF \cdot PA6_{purifyield} \cdot PA6_{wastefrac} \cdot years} + \frac{PA6_{purifyield} \cdot PA6_{wastefrac} \cdot (0.101258 \cdot CF \cdot years \cdot (0.0620547 \cdot \dot{m}_{H3PO4} + 0.50124) + 6.65266 \cdot 10^{-5} \cdot CF \cdot years \cdot (4624.11 \cdot \dot{m}_{H3PO4} + 2272.58) + 0.0187913)}{CF \cdot PA6_{purifyield} \cdot PA6_{wastefrac} \cdot years}$$

GWP calculated using average German electricity, without PA6 prepurification (only sorting) and hazardous waste treatment of process waste (reference: 4.054 kg CO_2 Eq.):

$$GWP[kg\ CO_2Eq.] = \frac{CF \cdot PA6_{purifyield} \cdot PA6_{wastefrac} \cdot years \cdot (7.14737 \cdot \dot{m}_{H3PO4} + 1.3424) + CF \cdot years \cdot (0.00114936 \cdot \dot{m}_{H3PO4} + 1.28481) \cdot \left(0.234696 \cdot PA6_{purifyield} \cdot PA6_{wastefrac} + PA6_{purifyield} \cdot \left(0.0866844 \cdot PA6_{wastefrac} + 0.0423478\right)\right) +}{CF \cdot PA6_{purifyield} \cdot PA6_{wastefrac} \cdot years}$$
$$\frac{PA6_{purifyield} \cdot PA6_{wastefrac} \cdot (0.101258 \cdot CF \cdot years \cdot (0.0620547 \cdot \dot{m}_{H3PO4} + 0.50124) + 6.65266 \cdot 10^{-5} \cdot CF \cdot years \cdot (4624.11 \cdot \dot{m}_{H3PO4} + 2272.58) + 0.0187913)}{CF \cdot PA6_{purifyield} \cdot PA6_{wastefrac} \cdot years}$$

GWP calculated using wind electricity, without PA6 prepurification (only sorting) and hazardous waste treatment of process waste (reference: 3.862 kg CO_2 Eq.):

$$GWP[kgCO_2Eq.] = \frac{CF \cdot PA6_{purifyield} \cdot PA6_{wastefrac} \cdot years \cdot (6.88124 \cdot \dot{m}_{H3PO4} + 1.32515) + CF \cdot years \cdot (0.00114936 \cdot \dot{m}_{H3PO4} + 1.28481) \cdot \left(0.234696 \cdot PA6_{purifyield} \cdot PA6_{wastefrac} + PA6_{purifyield} \cdot (0.0866844 \cdot PA6_{wastefrac} + 0.00167777)\right)}{CF \cdot PA6_{purifyield} \cdot PA6_{wastefrac} \cdot years} +$$

$$\frac{PA6_{purifyield} \cdot PA6_{wastefrac} \cdot (0.101258 \cdot CF \cdot years \cdot (0.0620547 \cdot \dot{m}_{H3PO4} + 0.50124) + 6.65266 \cdot 10^{-5} \cdot CF \cdot years \cdot (4624.11 \cdot \dot{m}_{H3PO4} + 2272.58) + 0.0187913)}{CF \cdot PA6_{purifyield} \cdot PA6_{wastefrac} \cdot years}$$

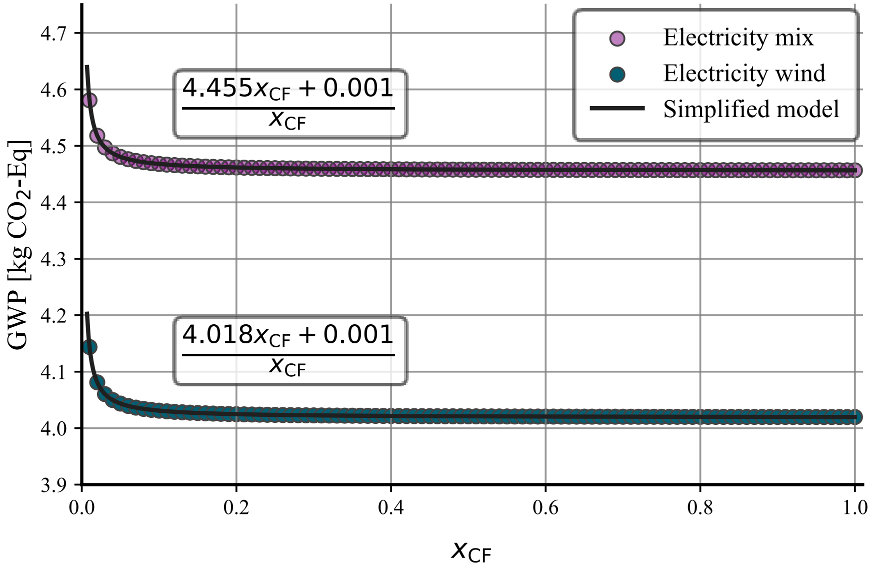

Figure D 9: GWP as a function of process capacity factor (x_{CF}) for PA6 re-monomerization with two electricity supply scenarios: average German electricity mix and wind electricity. Data points represent GWP results from the parametric LCA model at varying capacity factors, assuming reference conditions: H_3PO_4 input of 0.266 kg/s, 15 years operating time, PA6 waste purification (dissolution), hazardous process waste treatment, PA6 waste fraction of 0.5, and PA6 waste purification yield of 99%. Solid lines correspond to the surrogate equations (shown in the plot boxes) derived for each scenario. Results illustrate the effect of electricity source and plant utilization on climate impacts.

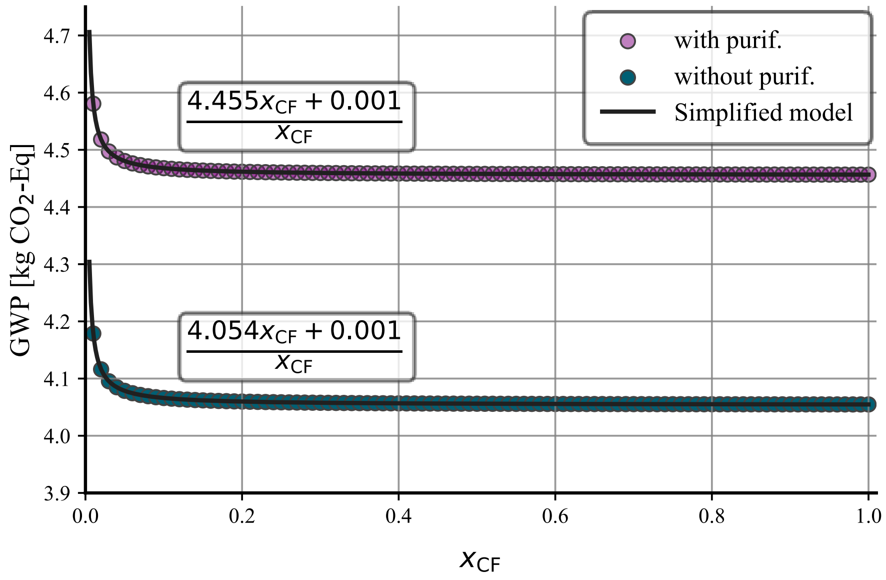

Figure D 10: GWP as a function of process capacity factor (x_{CF}) for PA6 re-monomerization with two PA6 waste prepurification scenarios: with and without waste purification. Data points represent GWP results from the parametric LCA model at varying capacity factors, assuming reference conditions: H_3PO_4 input of 0.266 kg/s, 15 years operating time, German electricity mix, hazardous process waste treatment, PA6 waste fraction of 0.5, and PA6 waste purification yield of 99%. Solid lines correspond to the surrogate equations (displayed in the plot boxes) derived for each scenario. Results illustrate the effect of PA6 waste pre-purification and plant utilization on climate impacts.

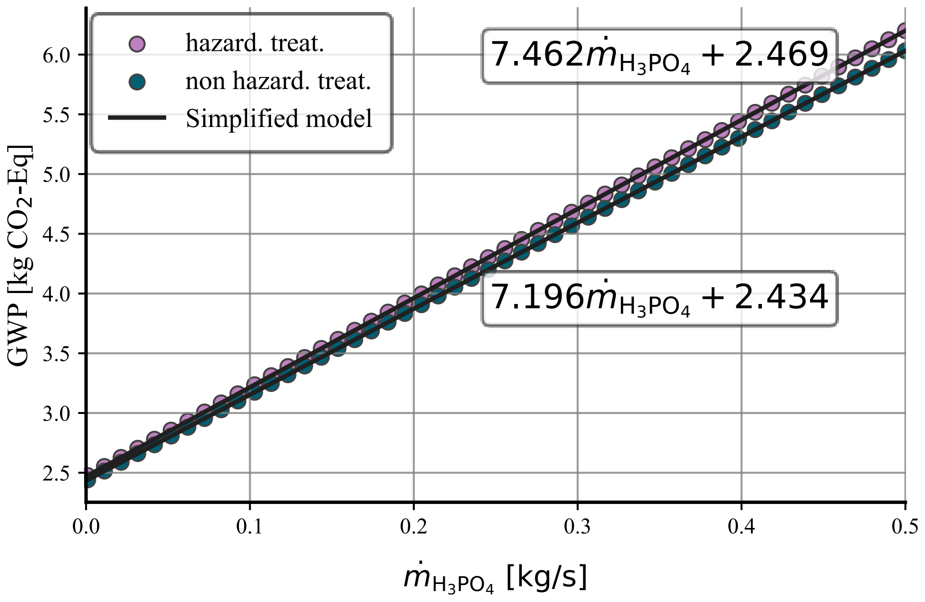

Figure D 11: GWP as a function of the phosphoric acid mass flow rate for PA6 re-monomerization, comparing two process waste treatment scenarios: hazardous waste treatment and non-hazardous waste treatment. Data points represent GWP results from the parametric LCA model at varying H_3PO_4 mass flow rates, assuming 15 years operating time, German electricity mix, full PA6 waste prepurification (dissolution), PA6 waste fraction of 0.5, and PA6 waste purification yield of 99%. Solid lines correspond to the surrogate equations (displayed in the plot boxes) derived for each scenario. The results demonstrate the influence of phosphoric acid usage and waste treatment strategy on the overall climate impact of the process.

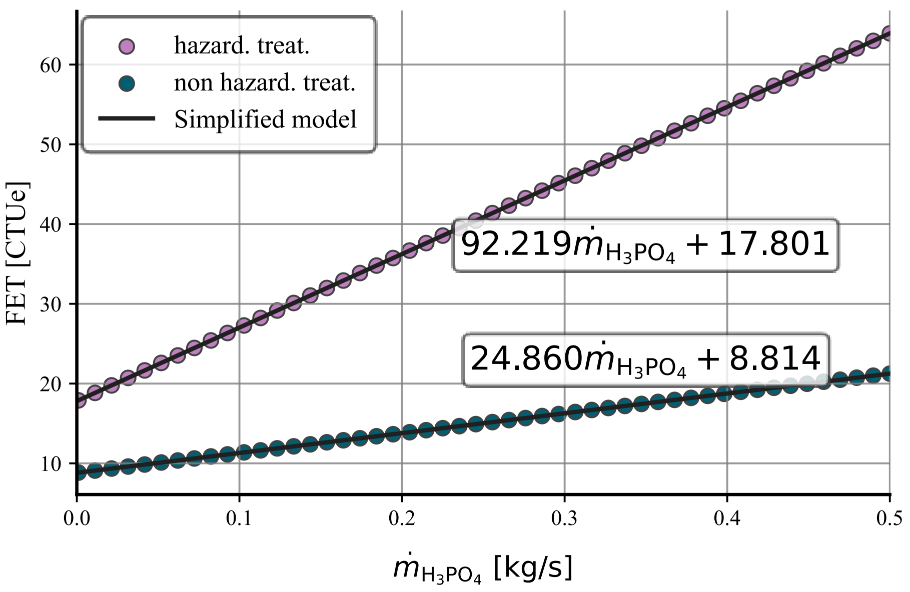

Figure D 12: Freshwater ecotoxicity (FET) as a function of the phosphoric acid mass flow rate for PA6 re-monomerization, comparing hazardous and non-hazardous waste treatment scenarios. Data points show FET results from the parametric LCA model at varying H_3PO_4 mass flow rates, assuming 15 years operating time, German electricity mix, full PA6 waste prepurification (dissolution), PA6 waste fraction of 0.5, and PA6 waste purification yield of 99%. Solid lines represent the corresponding surrogate equations (displayed in the plot boxes) for each scenario. The results illustrate the substantial influence of both phosphoric acid usage and waste treatment strategy on freshwater ecotoxicity impacts.

D.5.2 HTW Process

Reference setting: CF = 0.9, years = 15, m_{HTW} = 14.968 kg/s (corresponding to HTW:PA6 ratio of 30:1), conv = 0.89, $PA6_{wastefrac}$ = 0.5, $PA6_{purifyield}$ = 0.990

GWP calculated using average German electricity, with PA6 input waste sorting and PA6 prepurification (dissolution), process waste treatment as PET and reactor temperature at 345°C (reference: 13.053 kg CO_2-Eq.):

$$GWP[kg\ CO_2\ Eq.] = \frac{-2.03\,CF\,PA_{6,\text{purifyield}}PA_{6,\text{wastefrac}}years(0.22\,conv - 0.383) + PA_{6,\text{purifyield}}PA_{6,\text{wastefrac}}(-CF\,years(10.5\,conv - 20.7) + 6.01)}{CF\,PA_{6,\text{purifyield}}PA_{6,\text{wastefrac}}years} +$$

$$\frac{years\left(0.00333\,CF\,PA_{6,\text{purifyield}}PA_{6,\text{wastefrac}} - CF(1.47\,conv - 2.52)\left(0.235\,PA_{6,\text{purifyield}}PA_{6,\text{wastefrac}} + 0.0867\,PA_{6,\text{wastefrac}} + 0.174\right) + PA_{6,\text{purifyield}}PA_{6,\text{wastefrac}}(3.78\times10^{-5}CF + 5.13\times10^{-7}m_{HTW})\right)}{CF\,PA_{6,\text{purifyield}}PA_{6,\text{wastefrac}}years}$$

GWP calculated using wind electricity, with PA6 input waste sorting and PA6 prepurification (dissolution), process waste treatment as PET and reactor temperature at 345°C (reference: 12.404 kg CO_2-Eq.):

$$GWP[kg\ CO_2\ Eq.] = \frac{-2.03\,CF\,PA_{6,\text{purifyield}}PA_{6,\text{wastefrac}}years(0.22\,conv - 0.383) + PA_{6,\text{purifyield}}PA_{6,\text{wastefrac}}(-CF\,years(10.3\,conv - 20.2) + 6.01)}{CF\,PA_{6,\text{purifyield}}PA_{6,\text{wastefrac}}years} +$$

$$\frac{years\left(0.00333\,CF\,PA_{6,\text{purifyield}}PA_{6,\text{wastefrac}} - CF(1.47\,conv - 2.52)\left(0.235\,PA_{6,\text{purifyield}}PA_{6,\text{wastefrac}} + 0.0867\,PA_{6,\text{wastefrac}} + 0.0524\right) + PA_{6,\text{purifyield}}PA_{6,\text{wastefrac}}(3.78\times10^{-5}CF + 5.13\times10^{-7}m_{HTW})\right)}{CF\,PA_{6,\text{purifyield}}PA_{6,\text{wastefrac}}years}$$

GWP calculated using average German electricity, without PA6 input prepurification (only sorting), process waste treatment as PET and reactor temperature at 345°C (reference: 12.676 kg CO_2-Eq.):

$$GWP[kg\ CO_2\ Eq.] = \frac{-2.03\,CF\,PA_{6,\text{purifyield}}PA_{6,\text{wastefrac}}years(0.22\,conv - 0.383) + PA_{6,\text{purifyield}}PA_{6,\text{wastefrac}}(-CF\,years(10.5\,conv - 20.7) + 6.01)}{CF\,PA_{6,\text{purifyield}}PA_{6,\text{wastefrac}}years} +$$

$$\frac{years\left(0.00333\,CF\,PA_{6,\text{purifyield}}PA_{6,\text{wastefrac}} - CF(1.47\,conv - 2.52)\left(0.235\,PA_{6,\text{purifyield}}PA_{6,\text{wastefrac}} + PA_{6,\text{purifyield}}\left(0.0867\,PA_{6,\text{wastefrac}} + 0.0424\right)\right) + PA_{6,\text{purifyield}}PA_{6,\text{wastefrac}}(3.78\times10^{-5}CF + 5.13\times10^{-7}\dot{m}_{HTW})\right)}{CF\,PA_{6,\text{purifyield}}PA_{6,\text{wastefrac}}years}$$

GWP calculated using wind electricity, without PA6 input prepurification (only sorting), process waste treatment as PET and reactor temperature at 345°C (reference: 12.255 kg CO_2-Eq.):

$$GWP[kg\ CO_2\ Eq.] = \frac{-2.03\, CF\, PA_{6,\mathrm{purifyield}} PA_{6,\mathrm{wastefrac}} years(0.22\, conv - 0.383) + PA_{6,\mathrm{purifyield}} PA_{6,\mathrm{wastefrac}}(-CF\, years(10.3\, conv - 20.2) + 6.01)}{CF\, PA_{6,\mathrm{purifyield}} PA_{6,\mathrm{wastefrac}} years} +$$

$$\frac{years\left(0.00333\, CF\, PA_{6,\mathrm{purifyield}} PA_{6,\mathrm{wastefrac}} - CF(1.47\, conv - 2.52)\left(0.235\, PA_{6,\mathrm{purifyield}} PA_{6,\mathrm{wastefrac}} + PA_{6,\mathrm{purifyield}}\left(0.0867\, PA_{6,\mathrm{wastefrac}} + 0.00168\right)\right) + PA_{6,\mathrm{purifyield}} PA_{6,\mathrm{wastefrac}}(3.78 \times 10^{-5} CF + 5.13 \times 10^{-7} \dot{m}_{HTW})\right)}{CF\, PA_{6,\mathrm{purifyield}} PA_{6,\mathrm{wastefrac}} years}$$

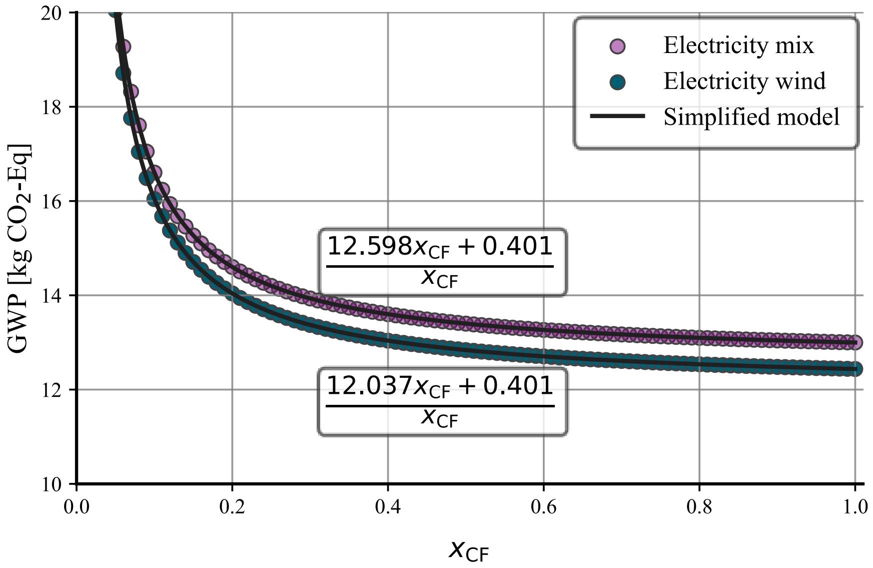

Figure D 13: GWP as a function of process capacity factor (x_{CF}) for PA6 re-monomerization with two electricity supply scenarios: average German electricity mix and wind electricity. Data points represent GWP results from the parametric LCA model at varying capacity factors, assuming reference conditions: HTW input of 14.95 kg/s, conversion of 0.89, reactor temperature of 345 °C, 15 years operating time, PA6 waste purification (dissolution), PA6 waste fraction of 0.5, and PA6 waste purification yield of 99%. Solid lines correspond to the surrogate equations (shown in the plot boxes) derived for each scenario. Results illustrate the effect of electricity source and plant utilization on climate impacts.

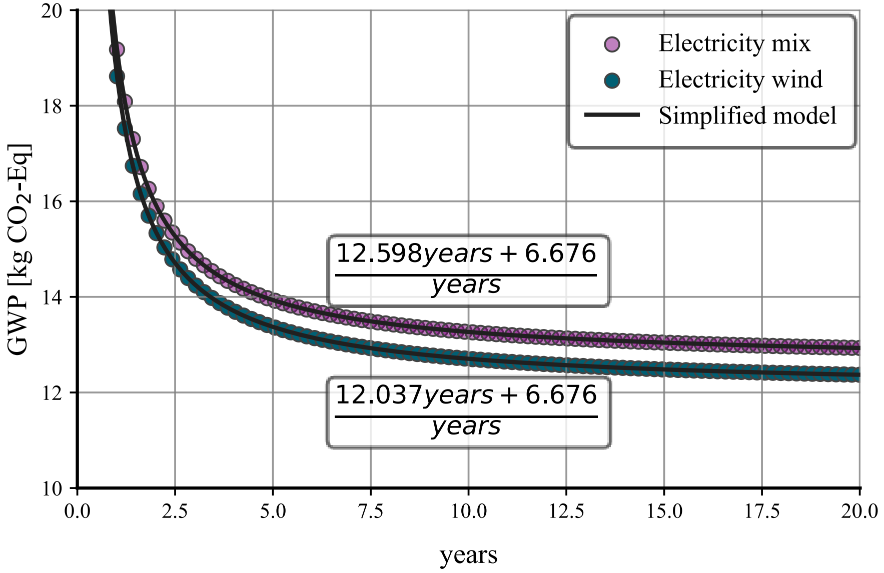

Figure D 14: GWP as a function of operating years for PA6 re-monomerization with two electricity supply scenarios: average German electricity mix and wind electricity. Data points represent GWP results from the parametric LCA model at varying capacity factors, assuming reference conditions: HTW input of 14.95 kg/s, conversion of 0.89, reactor temperature of 345 °C, a capacity factor of 0.9, PA6 waste purification (dissolution), PA6 waste fraction of 0.5, and PA6 waste purification yield of 99%. Solid lines correspond to the surrogate equations (displayed in the plot boxes) derived for each scenario. Results illustrate the effect of PA6 waste pre-purification and operating years on climate impacts.

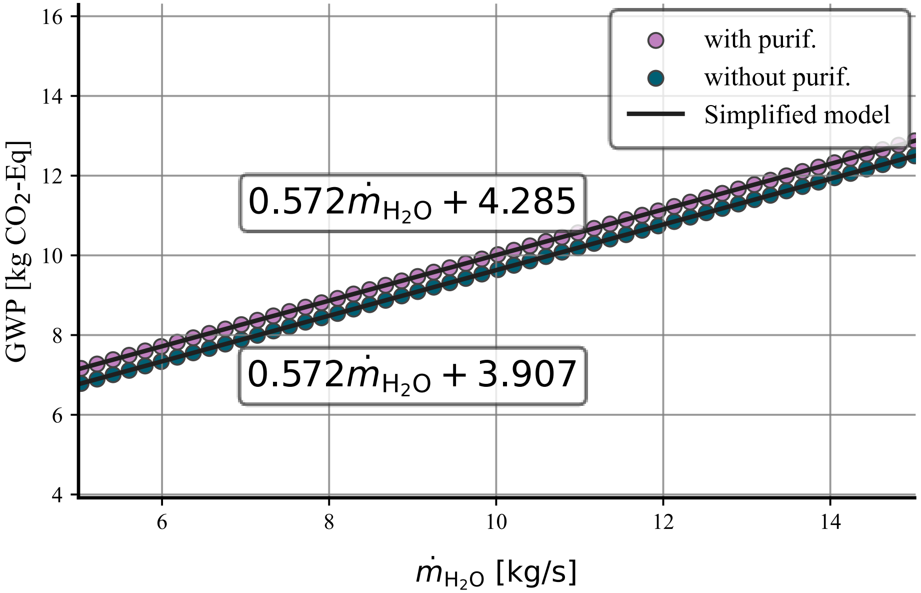

Figure D 15: GWP as a function of water mass flow rate for PA6 re-monomerization, comparing two scenarios: with and without PA6 waste purification. Data points represent GWP results from the parametric LCA model at varying water flow rates, assuming reference conditions: HTW input of 14.95 kg/s, conversion of 0.89, reactor temperature of 345 °C, 15 years operating time, a capacity factor of 0.9, German electricity mix, PA6 waste fraction of 0.5, and PA6 waste purification yield of 99%. Solid lines correspond to the surrogate equations (displayed in the plot boxes) derived for each scenario. The results demonstrate the influence of water usage and waste purification strategy on the climate impact of the process.

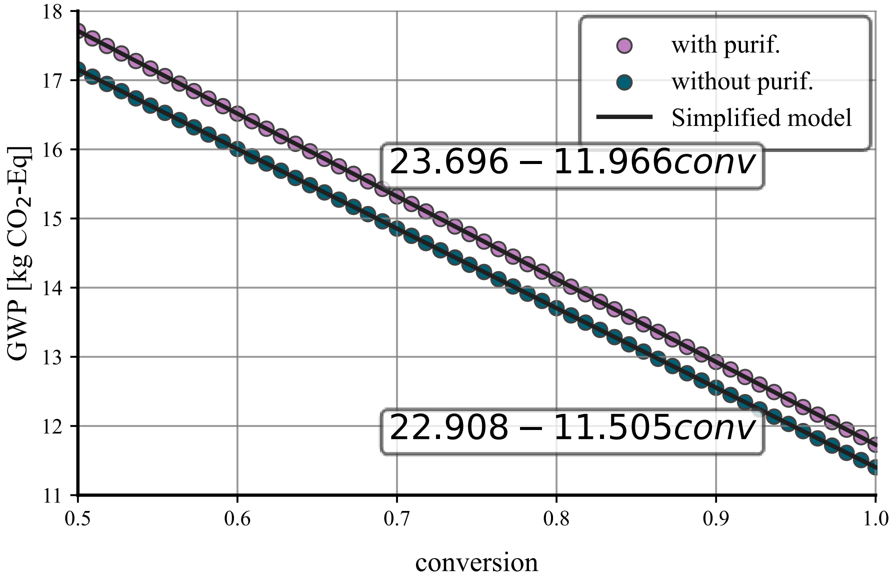

Figure D 16: GWP as a function of PA6 conversion for PA6 re-monomerization, comparing scenarios with and without PA6 waste purification. Data points represent GWP results from the parametric LCA model at varying conversion rates, assuming reference conditions: HTW input of 14.95 kg/s, reactor temperature of 345 °C, a capacity factor of 0.9, 15 years operating time, German electricity mix, PA6 waste fraction of 0.5, and PA6 waste purification yield of 99%. Solid lines correspond to the surrogate equations (displayed in the plot boxes) derived for each scenario. The results demonstrate the effect of process conversion and waste purification on the climate impact of the process.

D.5.3 iPrOH Process

Reference setting: CF = 0.9, years = 15, amt_{iPrOH} = 6.69 kg/s, $PA6_{wastefrac}$ = 0.5, $PA6_{purifyield}$ = 0.990

GWP calculated using average German electricity, with PA6 input waste sorting and PA6 prepurification (dissolution) and waste treatment of process waste (reference: 4.41 kg CO_2 Eq.):

$$GWP[kg\ CO_2\ Eq.] = \frac{CF \cdot PA6_{purifyield} \cdot PA6_{wastefrac} \cdot years \cdot (0.0154 \cdot \dot{m_{iPrOH}} + 2.68) + 1.22 \cdot CF \cdot years \cdot \left(0.0867 \cdot PA6_{wastefrac} + 0.174\right) + PA6_{purifyield} \cdot PA6_{wastefrac} \cdot (0.385 \cdot CF \cdot years - 1.49 \cdot 10^{-7} \cdot \dot{m_{iPrOH}} \cdot years \cdot (130.0 \cdot \dot{m_{iPrOH}} - 1.66 \cdot 10^4) + 8.43)}{CF \cdot PA6_{purifyield} \cdot PA6_{wastefrac} \cdot years}$$

GWP calculated using wind electricity, with PA6 input waste sorting and PA6 prepurification (dissolution) and waste treatment of process waste (reference: 3.867 kg CO_2 Eq.):

$$GWP\ [kg\ CO_2\ Eq.] = \frac{CF \cdot PA6_{purifyield} \cdot PA6_{wastefrac} \cdot years \cdot (0.0154 \cdot \dot{m_{iPrOH}} + 2.47) + 1.22 \cdot CF \cdot years \cdot \left(0.0867 \cdot PA6_{wastefrac} + 0.0524\right) + PA6_{purifyield} \cdot PA6_{wastefrac} \cdot (0.385 \cdot CF \cdot years - 1.49 \cdot 10^{-7} \cdot \dot{m_{iPrOH}} \cdot years \cdot (130.0 \cdot \dot{m_{iPrOH}} - 1.66 \cdot 10^4) + 8.43)}{CF \cdot PA6_{purifyield} \cdot PA6_{wastefrac} \cdot years}$$

GWP calculated using average German electricity, without PA6 waste input treatment (only sorting) and hazardous waste treatment of process waste (reference: 4.038 kg CO_2 Eq.):

$$GWP\ [kg\ CO_2\ Eq.] = \frac{CF \cdot PA6_{purifyield} \cdot PA6_{wastefrac} \cdot years \cdot (0.0154 \cdot \dot{m_{iPrOH}} + 2.68) + 1.22 \cdot CF \cdot PA6_{purifyield} \cdot years \cdot \left(0.0867 \cdot PA6_{wastefrac} + 0.0424\right)}{CF \cdot PA6_{purifyield} \cdot PA6_{wastefrac} \cdot years} + \frac{PA6_{purifyield} \cdot PA6_{wastefrac} \cdot (0.385 \cdot CF \cdot years - 1.49 \cdot 10^{-7} \cdot \dot{m_{iPrOH}} \cdot years \cdot (130.0 \cdot \dot{m_{iPrOH}} - 1.66 \cdot 10^4) + 8.43)}{CF \cdot PA6_{purifyield} \cdot PA6_{wastefrac} \cdot years}$$

GWP calculated using wind electricity, without PA6 waste input treatment (only sorting) and hazardous waste treatment of process waste (reference: 3.719 kg CO_2 Eq.):

$$GWP\ [kg\ CO_2\ Eq.] = \frac{CF \cdot PA6_{purifyield} \cdot PA6_{wastefrac} \cdot years \cdot (0.0154 \cdot \dot{m_{iPrOH}} + 2.47) + 1.22 \cdot CF \cdot PA6_{purifyield} \cdot years \cdot \left(0.0867 \cdot PA6_{wastefrac} + 0.00168\right)}{CF \cdot PA6_{purifyield} \cdot PA6_{wastefrac} \cdot years} + \frac{PA6_{purifyield} \cdot PA6_{wastefrac} \cdot (0.385 \cdot CF \cdot years - 1.49 \cdot 10^{-7} \cdot \dot{m_{iPrOH}} \cdot years \cdot (130.0 \cdot \dot{m_{iPrOH}} - 1.66 \cdot 10^4) + 8.43)}{CF \cdot PA6_{purifyield} \cdot PA6_{wastefrac} \cdot years}$$

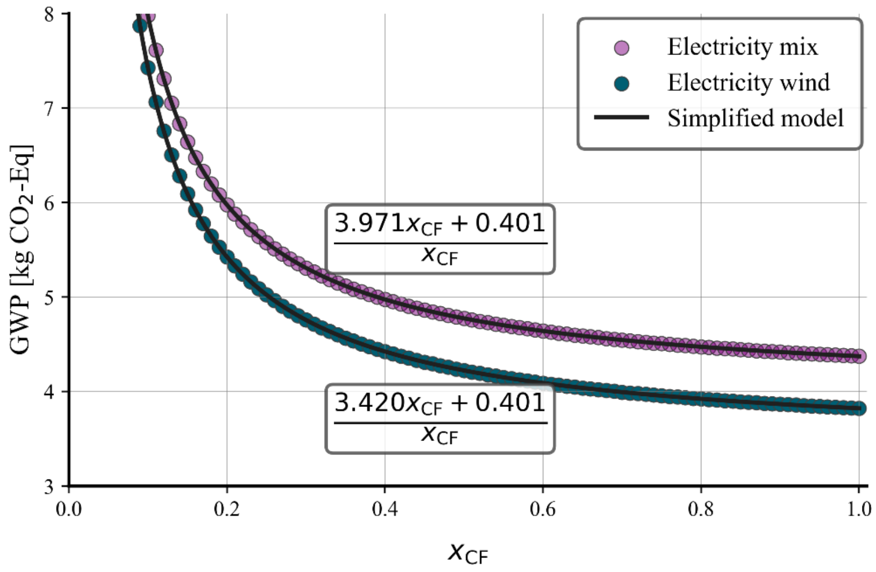

Figure D 17: GWP as a function of process capacity factor (x_{CF}) for PA6 re-monomerization with two electricity supply scenarios: average German electricity mix and wind electricity. Data points represent GWP results from the parametric LCA model at varying capacity factors, assuming the reference conditions iPrOH input of 6.69 kg/s, 15 years operating time, PA6 waste purification (dissolution), PA6 waste fraction of 0.5, and PA6 waste purification yield of 99%. Solid lines correspond to the surrogate equations (shown in the plot boxes) derived for each scenario. Results illustrate the effect of electricity source and plant utilization on climate impacts.

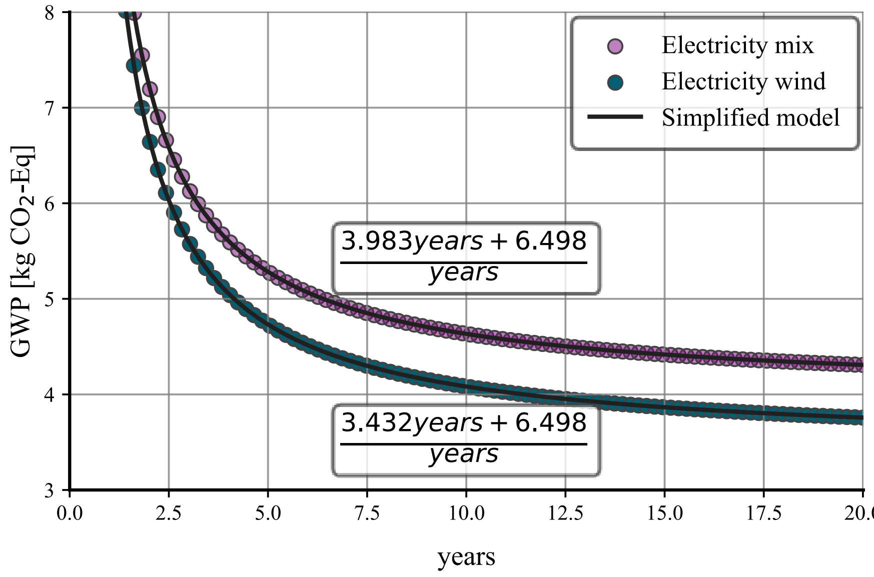

Figure D 18: GWP as a function of operating years for PA6 re-monomerization with two electricity supply scenarios: average German electricity mix and wind electricity. Data points represent GWP results from the parametric LCA model at varying number of operating years, assuming the reference conditions iPrOH input of 6.69 kg/s, 15 years operating time, PA6 waste purification (dissolution), PA6 waste fraction of 0.5, and PA6 waste purification yield of 99%. Solid lines correspond to the surrogate equations (displayed in the plot boxes) derived for each scenario. Results illustrate the effect of electricity source and operating time on climate impacts.

D.5.4 NaOH Process

Reference setting: CF = 0.9, years = 15, $CL_{recovery}$ = 0.9967, $PA6_{wastefrac}$ = 0.5, $PA6_{purifyield}$ = 0.990

GWP calculated using average German electricity, with PA6 input waste sorting and PA6 prepurification (dissolution), and process waste treatment as hazardous waste (reference: 1.47 kg CO_2-Eq.):

$$GWP[kg\ CO_2\ Eq.] = \frac{-CF \cdot PA6_{purifyield} \cdot PA6_{wastefrac} \cdot years \cdot (23.3 \cdot CL_{recovery} - 23.9) - CF \cdot years \cdot (1.13 \cdot CL_{recovery} - 2.24) \cdot (0.235 \cdot PA6_{purifyield} \cdot PA6_{wastefrac} + 0.0867 \cdot PA6_{wastefrac} + 0.174) + 0.00847 \cdot PA6_{purifyield} \cdot PA6_{wastefrac}}{CF \cdot PA6_{purifyield} \cdot PA6_{wastefrac} \cdot years}$$

GWP calculated using wind electricity, with PA6 input waste sorting and PA6 prepurification (dissolution), process waste treatment as hazardous waste (reference: 0.972 kg CO_2-Eq.):

$$GWP\ [kg\ CO_2\ Eq.] = \frac{-CF \cdot PA6_{purifyield} \cdot PA6_{wastefrac} \cdot years \cdot (22.9 \cdot CL_{recovery} - 23.3) - CF \cdot years \cdot (1.13 \cdot CL_{recovery} - 2.24) \cdot (0.235 \cdot PA6_{purifyield} \cdot PA6_{wastefrac} + 0.0867 \cdot PA6_{wastefrac} + 0.0524) + 0.00847 \cdot PA6_{purifyield} \cdot PA6_{wastefrac}}{CF \cdot PA6_{purifyield} \cdot PA6_{wastefrac} \cdot years}$$

GWP calculated using average German electricity, without PA6 input waste prepurification (only sorting), process waste treatment as hazardous waste (reference: 1.127 kg CO_2-Eq.):

$$GWP[kg\ CO_2\ Eq.] = \frac{-CF \cdot PA6_{purifyield} \cdot PA6_{wastefrac} \cdot years \cdot (23.3 \cdot CL_{recovery} - 23.9) - CF \cdot years \cdot (1.13 \cdot CL_{recovery} - 2.24) \cdot \left(0.235 \cdot PA6_{purifyield} \cdot PA6_{wastefrac} + PA6_{purifyield} \cdot (0.0867 \cdot PA6_{wastefrac} + 0.0424)\right) + 0.00847 \cdot PA6_{purifyield} \cdot PA6_{wastefrac}}{CF \cdot PA6_{purifyield} \cdot PA6_{wastefrac} \cdot years}$$

GWP calculated using wind electricity, without PA6 input waste prepurification (only sorting), process waste treatment as hazardous waste (reference: 0.835 kg CO_2-Eq.):

$$GWP[kg\ CO_2\ Eq.] = \frac{-CF \cdot PA6_{purifyield} \cdot PA6_{wastefrac} \cdot years \cdot (22.9 \cdot CL_{recovery} - 23.3) - CF \cdot years \cdot (1.13 \cdot CL_{recovery} - 2.24) \cdot \left(0.235 \cdot PA6_{purifyield} \cdot PA6_{wastefrac} + PA6_{purifyield} \cdot (0.0867 \cdot PA6_{wastefrac} + 0.00168)\right) + 0.00847 \cdot PA6_{purifyield} \cdot PA6_{wastefrac}}{CF \cdot PA6_{purifyield} \cdot PA6_{wastefrac} \cdot years}$$

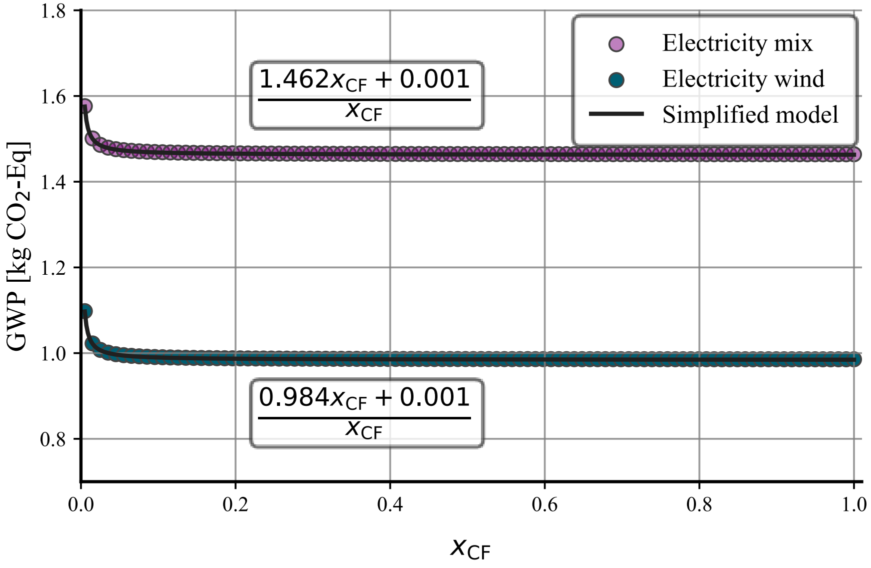

Figure D 19: GWP as a function of process capacity factor (x_{CF}) for PA6 re-monomerization with two electricity supply scenarios: average German electricity mix and wind electricity. Data points represent GWP results from the parametric LCA model at varying capacity factors, assuming the reference conditions: 15 years operating time, PA6 waste purification (dissolution), PA6 waste fraction of 0.5, PA6 waste purification yield of 99% and CL recovery of 99.7%. Solid lines correspond to the surrogate equations (shown in the plot boxes) derived for each scenario. Results illustrate the effect of electricity source and plant utilization on climate impacts.

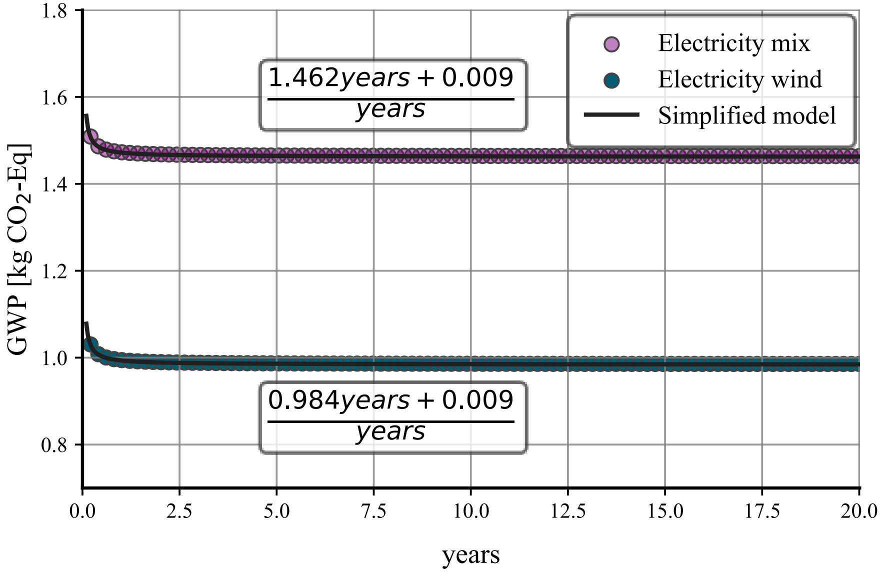

Figure D 20: GWP as a function of operating years for PA6 re-monomerization with two electricity supply scenarios: average German electricity mix and wind electricity. Data points represent GWP results from the parametric LCA model at varying number of operating years, assuming the reference iPrOH input, a capacity factor of 0.9, PA6 waste purification (dissolution), PA6 waste fraction of 0.5, PA6 waste purification yield of 99% and CL recovery of 99.7%. Solid lines correspond to the surrogate equations (displayed in the plot boxes) derived for each scenario. Results illustrate the effect of electricity source and operating time on climate impacts.

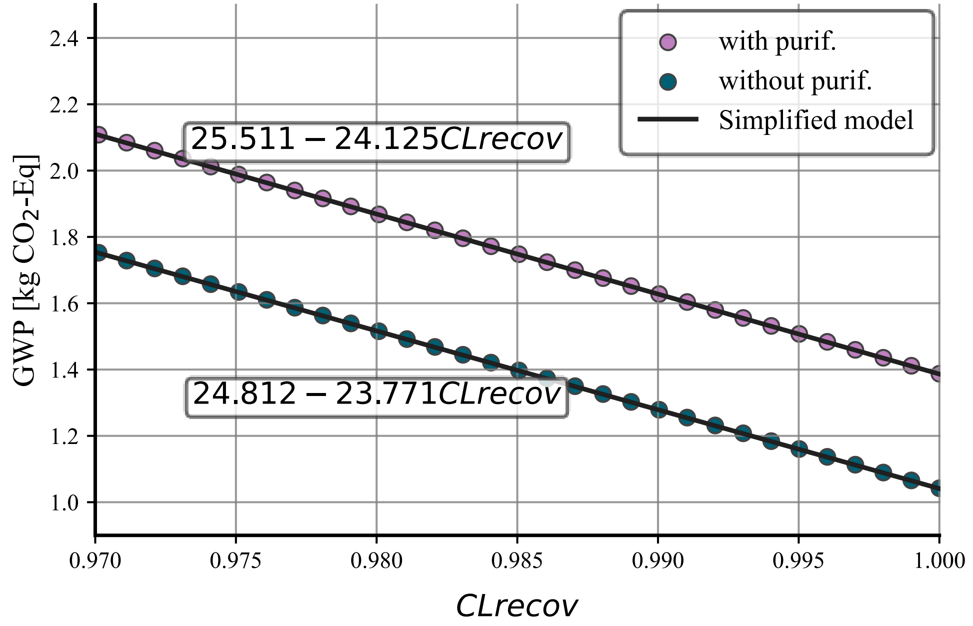

Figure D 21: GWP as a function of CL recovery for PA6 re-monomerization comparing two scenarios: with and without PA6 waste purification. Data points represent GWP results from the parametric LCA model at CL recovery, assuming reference conditions: capacity factor of 0.9, 15 years operating time, PA6 waste purification (dissolution), PA6 waste fraction of 0.5, PA6 waste purification yield of 99% and CL recovery of 99.7%. Solid lines correspond to the surrogate equations (displayed in the plot boxes) derived for each scenario. The results demonstrate the influence of CL recovery and waste purification strategy on the climate impact of the process.

D.6 Absolute Evaluation of GWP against a Carbon Budget

Table D 5: Annual GWP emissions of the four different chemical recycling pathways, calculated based on predicted PA6 production volumes, assuming that all produced PA6 will ultimately enter as waste for the chemical recycling.

$\mathbf{GWP_{CL,annual}}(t)$ **[Mt CO_2 Eq / year]** $GWP_{CL,annual}(t) = GWP_{CL} * \alpha_{PA6toCL} * M_{PA6}(t)$		**H_3PO_4**	**HTW**	**iPrOH**	**NaOH**
**2025**	**Minimum**	9.54	20.05	9.24	2.41
	**Reference**	11.01	35.09	11.31	4.17
	**Maximum**	11.22	32.85	11.74	4.98
**2030**	**Minimum**	10.49	22.03	10.15	2.64
	**Reference**	12.10	38.55	12.43	4.58
	**Maximum**	12.33	36.10	12.90	5.47
**2050**	**Minimum**	15.28	32.10	14.79	3.85
	**Reference**	17.63	56.17	18.11	6.68
	**Maximum**	17.96	52.60	18.80	7.97

When divided by the annual carbon budget for CL production ($CB_{CLforPA6}$), the resulting ASR provides a unitless benchmark to assess whether the chemical recycling process remains within the carbon budget constraints defined by the 1.5°C climate target.

Table D 6: Absolute Sustainability Ratios (ASR) for the four different chemical recycling pathways across various years.

$\mathbf{ASR_{CLforPA6}}$ **[-]** $ASR_{CLforPA6} = \frac{GWP_{CL,annual}(2050)}{CB_{CLforPA6}(2050)}$		***H_3PO_4***	***HTW***	***iPrOH***	***NaOH***
**2025**	**Minimum**	4.79	10.07	4.64	1.21
	**Reference**	5.53	17.62	5.68	2.09

	**Maximum**	5.63	16.50	5.90	2.50
**2030**	**Minimum**	7.64	22.67	7.39	1.93
	**Reference**	8.81	28.07	9.05	3.34
	**Maximum**	8.98	37.15	9.40	3.98
**2050**	**Minimum**	59.37	124.73	57.46	14.97
	**Reference**	68.49	218.25	70.38	25.94
	**Maximum**	69.79	204.36	73.04	30.95

The GWP targets required to achieve an ASR of 1, thereby ensuring alignment with the carbon budget for CL production, were calculated for different years and presented in Table D 7.

Table D 7: GWP targets required to achieve an ASR of 1 across various years.

**GWP_{target} [kg CO_2 Eq / kg CL]**	
2025	0.607
2030	0.381
2050	0.049

E Methodology

E.1 Variable Mixed Plastic Waste Modeling

In line with established LCA practice, we distinguish between the background system (processes not under direct operational control) and the foreground system (simulated in detail using Aspen Plus). Prepurification steps, including sorting, and pre-purification via density separation and dissolution, were included in the background system using literature-based datasets, while the foreground system focuses on the chemical depolymerization stage and assumes purified PA6 input. This approach allows for isolation and fair comparison of the recycling chemistries under harmonized conditions, without excluding upstream burdens from the system boundaries.

Sorting activities are modeled using a dataset for the sorting and shredding of fishing gear containing PA6 (used as the reference), along with proxy datasets of PE, PET, and mixed plastic waste. Importantly, we did not rely on a single dataset; rather, we systematically varied these scenarios in both the impact probability distributions and error bars around a reference status. Further details on the sorting datasets can be found in Supplement Section B.2.1.

Regarding the prepurification, we include density separation with washing, based on industrial data for PA6 fishing gear (Schneider et al., [5]) and dissolution-based separation (Costamagna et al., [7]). Both scenarios capture the full environmental burdens, including utilities, energy use, and material handling, based on the entire mass processed, not only the target material. Although these steps yield valuable co-products (e.g., pure PE), we assign no credit for avoided PE production, in line with the LCA cut-off approach, resulting in a conservative modeling choice. Both options are now varied in the sensitivity and Monte Carlo analyses to capture uncertainty related to real-world PA6 feedstock.

We not only varied the prepurification datasets, but also the PA6 content in the input waste, modeled as a continuous parameter ranging from 0.3 to 0.9 and the PA6 waste pre-purification yield, varied between 0.85 and 0.99 (Supplement Table B.1, manuscript Figure 1).

E.2 Life Cycle Assessment

E.2.1 Goal and Scope Definition

Table E 1: Explanations, arguments, and justifications for the methodological choices, including the LCA approach, scope and system boundaries.

Name	Choice	Reasons / Argumentation
Functional unit	1 kg CL with at least 99.9 wt% purity	· follows the product-centric approach recommended by Reuter et al. (2013) [34] and Schrijvers et al. (2021) [35]
LCA Approach	Attributional LCA	· Focused on quantifying life cycle impacts of CL production, not system-wide decisions like substitution of fossil-based production.<br>· Market and industrial production data for consequential LCA is unavailable.<br>· Assumed that processes will have large-scale consequences on the installed capacity of the background system
Scope	Gate (Nylon 6 waste) to factory gate (CL)	· Downstream use and end-of-life treatment of CL not relevant for meaningful insights as they are identical across the four processes to be compared
Geographical scope	Priority order: First Germany, then Europe without Switzerland then Europe including Switzerland (RER)	· High concentration of LCA studies and datasets in Europe [17]<br>· Industrial relevance as being large market in Europe for plastic production [36]<br>· In cases where German-specific data is unavailable, the priority cascade to ensure the closest approximation to German conditions
Transportation	1000 km transport by lorry within Europe	· To address the transportation of goods within Europe to the German site such that regional logistical activities are adequately represented

Allocation Approach	Cut-off method	· Explicit, well-justified, and relevant to decision-makers while providing reproducible and generalizable results according to Ekvall et all (2021) comparing 12 different allocation methods [37]<br>· Most widely used approach in recycling studies [35,38,39]<br>· Assigning impacts from the previous life cycle to the recycling process can result in the recycled product having higher overall impacts, even when the recycling process itself has lower emissions<br>· Lack of industrial pricing for PA6 waste and absence of a justified factor prevent using methods like APOS for impact distribution between primary and recycling life cycles [38]
System boundaries - PA6 waste input	No exact waste composition is specified (treated as a pure PA6 stream in simulations), but impacts of pre-processing steps are estimated, including collection, sorting, transportation of PA6 waste from consumers, and optional dissolution treatment.	· Fair and consistent comparison between four different pathways as variations in PA6 conversion efficiency directly affect PA6 flow rates and, consequently, the overall collection and sorting impacts<br>· Enables a fair distinction from linear pathways (e.g., incineration), as collection and sorting efforts are higher for chemical recycling processes<br>· Aligns with EPD recommendation which define boundary at the point where the material has its lowest market value [37]<br>· Consistent with cut off approach in ecoinvent where the boundary is set at the point of recyclable material production [13]<br>· Variability in PA6 waste composition across regions, as highlighted by Davidson et al. (2021) [40], makes specifying a single composition impractical, ensuring broader applicability and comparability.<br>· Optional dissolution is included as collection and sorting processes may not consistently achieve the purity required for chemical recycling processes.
System boundaries - process waste streams treatment	The full impacts of waste treatment are considered, with no credits applied in cases of multifunctionality	· According to "polluter pays" principle [13]<br>· Can lead to double counting of recycling benefits<br>· Crediting the system with avoided impacts from energy production elsewhere [41] is inconsistent with the attributional LCA's goal of reflecting current conditions rather than hypothetical market dynamics [39,42]

E.2.2 Description of Impact Categories

Table E 2: mid-point indicators with abbreviations, units, descriptions, and robustness classes

Name	Abbreviation	Description	Unit	Robustness [43]	Reference
Global warming potential	GWP	converts greenhouse-gas emissions to CO_2-equivalents (kg CO_2-eq) using the 100-year global warming potential (GWP100), which compares the time-integrated radiative forcing from a 1-kg pulse emission of each gas to that from 1 kg of CO_2. Characterization factors reflect gas-specific radiative efficiency and atmospheric lifetime.	kg CO_2-eq	1	[44]
Freshwater ecotoxicity	FET	estimates the potential impact of chemical emissions on freshwater organisms (e.g., algae, invertebrates, fish) due to their persistence, mobility, and toxicity. It is based on the USEtox framework, which combines how substances move in the environment (fate), how organisms come into contact with them (exposure), and how harmful they are (effects), using generic, non site-specific settings. CTUe expresses the modeled potentially affected fraction of species, integrated over time and water volume, per kilogram emitted. It is a relative screening indicator, not a measured concentration, a site-specific risk level, or a count of species actually harmed.	CTUe	3	[45]
Freshwater eutrophication potential	EP	estimates how nutrient emissions can over-fertilize rivers and lakes, triggering algal blooms, oxygen depletion, and ecosystem degradation. In EF 3.1, EP focuses on phosphorus because P is generally the limiting nutrient in European freshwaters. Characterization factors are derived from the CARMEN model, which simulates how nutrients from point and diffuse sources travel through soils, groundwater, and river networks across European catchments. EF 3.1 uses regionally averaged European factors, so the indicator is region-specific rather than globally transferable. EP is a relative potential, not a measured concentration or a prediction of an actual bloom.	kg P-eq	2	[46]
Carcinogenic human toxicity	HTC	estimates the potential increase in cancer cases in the population from a chemical emitted to air, water, or soil. EF 3.1 bases HTC on the USEtox® 2.1 framework, which combines how substances move and persist in the environment (fate), how people take them in (exposure via air, water, and food), and how harmful they are (cancer dose response). Results are reported in CTUh, interpreted as a modeled increase in population cancer cases-equivalent per kilogram emitted. This is a screening-level indicator intended for relative comparisons, not a measured risk at a specific place or time.	CTUh	3	[46]

Non-carcinogenic human toxicity	HTNC	estimates the potential increase in non-cancer health impacts (e.g., developmental, neurological, organ effects) from a chemical emitted to air, water, or soil. EF 3.1 bases HTNC on the USEtox framework (fate-exposure-effect) with generic, non-site-specific settings. Results are reported in CTUh, interpreted as a modeled increase in population non-cancer cases-equivalent per kilogram emitted. This is a screening, comparative indicator rather than a measured risk at a specific place or time. Metals are included, but their results carry higher uncertainty due to speciation and background-level issues.	CTUh	3	[46]
Ionizing radiation on human health	IR	estimates the potential human-health impact from routine (non-accidental) releases of radionuclides over the life cycle (e.g., uranium mining, fuel fabrication, power generation, waste handling). EF 3.1 models how airborne and waterborne radionuclides disperse and lead to population exposure under generic, non-site-specific conditions. Results are reported in kBq U-235-eq, which express each radionuclide's potential to cause human exposure relative to the same activity of uranium-235.	kBq U235-eq	2	[47]
Land use	LU	estimates the potential loss of soil functions caused by land occupation (use over time) and transformation (change of land type). EF 3.1 builds on a modified LANCA® v2.5 soil - quality approach. It models changes in four functions - erosion resistance, mechanical filtration, groundwater recharge, and biotic production - relative to a natural reference state. For each land use activity, the characterization factor represents the deficit in these functions relative to a natural reference state (usually undisturbed soil). These sub-indicators are normalized and aggregated into a single dimensionless soil-quality index ("points"), so results reflect quality loss, not area alone.	(-)	3	[48]
Resource use, minerals and metals -Abiotic depletion potential *	ADP	estimates how much extracting minerals and metals today contributes to long-term scarcity and potential loss of future availability for society. In EF 3.1, ADP follows the CML approach: characterization factors are calculated as a function of how much of the resource is left (the chosen reserve metric) and how fast it is extracted. Each mineral or metal is assigned a characterization factor (CF) that expresses how strongly its extraction contributes to future scarcity. Results are expressed relative to antimony (Sb).	kg Sb-eq	3	[49]
Water use - Water deprivation potential *	WDP	estimates the potential deprivation of other users (humans or aquatic ecosystems) caused by consumptive freshwater use (water that is evaporated, incorporated, or not returned locally). EF 3.1 applies the AWARE method, which computes a regional characterization factor from the available water remaining in a watershed after human and environmental flow needs are met. Scarcer regions get higher factors. Results are	m^3 world-eq deprived	3	[50]

		then reported as m³ world-eq deprived e.g., 5 m³ world-eq means that consuming 1 m³ there is five times more scarcity-critical than the global average.<br>This is not a physical volume of process water but a scarcity-weighted indicator that can vary strongly by region and database assumptions. Results should therefore be used for relative comparison within this study (which process stresses water more) and not as absolute water-use figures.			

* The results of this environmental impact indicator shall be used with care as the uncertainties of the results are high and as there is limited experience with the indicator.

E.2.3 Choice of Impact Categories

In accordance with the European Commission's Product Environmental Footprint (PEF) methodology, the selection of the most relevant impact categories should be based on normalized and weighted results. The recommended approach identifies categories that cumulatively contribute at least 80% to the total environmental impact, ensuring a focus on the most significant environmental burdens. The methodology mandates that at least three impact categories must be selected as relevant, and once identified, they cannot be removed. This structured approach is designed to enhance comparability and decision-making in LCA, ensuring that key environmental impacts are adequately addressed [51].

For the processes, all 16 impact categories from the EF 3.1 method were normalized using the normalization factors provided by Zampori and Pant (2019) [51] per capita, and the top 80% contributors were selected. Using this method, the following impact categories were identified as relevant for at least one of the four processes: HTC, HTNC, GWP, LU, and FEP. Selecting these five categories ensured that for all four processes, more than 80% of total impacts were covered. Additionally, impact categories FET, IR, ADP and WDP were chosen following the recommendations of Gibon and Hahn Menacho (2023) [52].

The following table gives further justification for why the following impact categories were not chosen.

Table E 3: Some impact categories that were excluded and reason for their exclusion

Impact category	Reason for exclusion
Ozone depletion potential	· Ozone-depleting substances and refrigerants or aerosol propellants are not used<br>· Impact category is often excluded [53]<br>· Results are largely driven by background datasets; due to the Montreal Protocol phase-out, outcomes are sensitive to data age rather than current foreground emissions, so this category has limited informative value here and should be de-prioritized in comparisons [43]
Particulate matter	· No combustion at large scale such as in power plants<br>· Utilities used in the processes effect both, GWP and particulate matter similiarly, and GWP is included in the analysis. As they are correlated for these utilities, including particulate matter does not give new insights
Terrestrial eutrophication	· Key nutrient pollution impacts are already accounted for by EP category<br>· Terrestrial eutrophication is most relevant for processes with direct ammonia or nitrogen oxide emissions, which are more common in agriculture, fertilizer production, and livestock farming.
Marine Eutrophication	· Processes do not involve direct nutrient loading into marine environments<br>· Impact is already addressed in terms of nutrient emissions through EP, and separating marine eutrophication does not provide additional insights
Resource use, fossils	· driven using fossil fuels and feedstocks and thus often shows a similar tendency as the climate change indicator

E.3 Absolute Evaluation of GWP against a Carbon Budget

To contextualize the environmental performance of the chemical recycling processes examined in this study, the respective GWP results were compared against an annual carbon budget specifically allocated to CL production for PA6 polymerization. This approach followed the concept of the absolute sustainability ratio (ASR), where environmental impacts are evaluated relative to a defined environmental space [54]. Previous studies have applied similar ASR-based methods to assess whether industrial activities remain within their fair share of global environmental limits [55].

This evaluation aimed to determine whether the environmental impacts of chemical recycling processes align with the allocated carbon budget under a 1.5°C climate scenario, using a ASR as a unitless benchmark. An ASR > 1 indicates that CL production exceeds its allocated carbon budget, while ASR ≤ 1 suggests that production aligns with the 1.5°C scenario.

E.3.1 General Approach

The starting point for this annual carbon budget was based on the Intergovernmental Panel on Climate Change (IPCC) Sixth Assessment Report (AR6) [44], which defines a global carbon budget - the maximum allowable CO_2 emissions required to limit global warming to 1.5°C. Annualized by another research group, we then systematically downscaled this annual carbon budget in three key steps to derive the annual carbon budget specifically allocated to CL production for PA6 polymerization $CB_{CLforPA6}$.

1. According to Bachmann et al. (2023) [56], the plastic industry is responsible for approximately 1.1% of global carbon emissions. Therefore, 1.1% of the global carbon budget was allocated to the plastics sector.
2. Within this plastic-sector budget, the specific share allocated to PA6 production was determined based on its projected global production volume ($M_{PA6}(t)$) relative to the total projected plastic production volume ($\sum_i M_{Plastics}$) in 2050.
3. The carbon budget allocated to PA6 production ($CB_{CLforPA6}$) was further refined to represent only the share of emissions associated with CL production for PA6 polymerization. We did this using an emission based allocation factor (β_{GWP}). This was calculated as the GWP of fossil-based CL production divided by the total GWP from CL-to-PA6 polymerization and PA6 end-of-life treatment, thereby approximating CL's relative contribution to the overall life cycle emissions. A conversion factor ($\alpha_{CLtoPA6}$) was applied to adjust for mass-based inefficiencies and stoichiometry between CL input and PA6 output.

E.3.2 Assumptions

This analysis is based on several key assumptions, each of which is justified by established practices in LCA, climate policy, or industry data. The allocation of the plastics-sector carbon budget to PA6 was carried out on the basis of projected annual production volumes. Specifically, the share attributed to PA6 was assumed to be proportional to its predicted output relative to total plastics production. This mass-based approach is standard in LCA and industry reporting, as it offers a straightforward and transparent means of distributing impacts among materials with broadly similar use profiles. While this method does not capture differences in product function or lifespan, it is justified by its prevalence in literature and its practicality in scenarios with limited functional data.

To the best of our knowledge, there is no scientific consensus on the most appropriate method for allocating shares of a global carbon budget across different industrial processes, such as allocation applied to downscale the global carbon budget for CL production dedicated to PA6 polymerization. Estimating this fraction is challenging due to multiple stages in PA6's life cycle - CL synthesis, polymerization, and end-of-life treatment - as well as variations in emissions, resource use, technology, and process efficiencies. In the absence of explicit data, an approximate allocation factor (β, expressed in kg PA6 / kg CL) was derived using GWP ratios, calculated as the emissions associated with the commercial fossil-based CL production divided by the sum of emissions from the commercial CL-to-PA6 polymerization process and the PA6 EoL treatment [57]. Results showed that even when using another allocation factor for downscaling the global carbon budget for CL production dedicated to PA6 polymerization, the results that ASR cannot be below 1 remain solid. This approach assumes fossil-based production serves as the benchmark due to its well-documented and industrial dominance.

Since the vast majority of CL produced goes into PA6 production, it is both reasonable and conservative to allocate the full caprolactam carbon budget to PA6 production for the purposes of this study.

E.3.3 Formulas

The following table provides an overview of the calculation procedure, including the parameters and variables used, along with their meanings, units, values, and the corresponding references from which the data was sourced.

Table D 8: Overview of the calculation procedure, including the parameters and variables used, along with their meanings, units, values, and the corresponding references from which the data was sourced.

Expression	Meaning	Unit	Value 2050	Value 2030	Value 2025	Reference / Calculation
**CB(t)**	Global annual carbon budget for year t, representing the maximum allowable CO_2 emissions to stay within a 1.5°C warming scenario.	Mt CO_2-eq / year	8487.46	31555.84	42029.34	[44,55]
$\mathbf{F_{plastic}}$	Fraction of the global carbon budget allocated to the plastic industry.	/	0.01	0.01	0.01	[56]
$\mathbf{M_{PA6}(t)}$	Predicted annual production volume of PA6 in year t.	Mt / year	5.10	3.50	3.19	[56], for 2025: $M_t = M_0 \cdot (1+r)^{(t-t_0)}$ with r as annual growth rate given by [56]
$\sum_i \mathbf{M_{Plastics}}$	Total predicted annual production capacity of all plastic types in year t.	Mt / year	1418.10	678.20	566.86	[56], for 2025: $M_t = M_0 \cdot (1+r)^{(t-t_0)}$ with r as annual growth rate given by [56]
$\mathbf{GWP_{fossilCLProduction}}$	Global warming potential per kg of CL produced from fossil-based feedstock.	kg CO_2 Eq / kg CL	6.91	6.91	6.91	[58]
$\mathbf{GWP_{fossilPA6Production}}$	Global warming potential per kg of polyamide PA6 produced from fossil-based feedstock.	kg CO_2 Eq / kg PA6	9.28	9.28	9.28	Ecoinvent dataset

β_{GWP}	Allocation factor representing the fraction of the PA6 carbon budget attributable to CL production, derived from GWP ratios.	kg PA6 / kg CL	0.74	0.74	0.74	$\frac{GWP_{fossilCLProduction}}{GWP_{fossilPA6Production}}$
$\alpha_{CLtoPA6}$	Mass-based conversion factor representing the mass ratio of PA6 produced per unit of CL used.	kg PA6 / kg CL	0.97	0.97	0.97	[57]
$CB_{CLforPA6}$	Annual carbon budget allocated specifically to CL production for PA6 polymerization.	Mt CO_2 Eq / year	0.25	1.34	1.99	$CB(2050) \cdot 0.011 \cdot \frac{M_{PA6}(t)}{\sum_i M_{Plastics}} \cdot \frac{\beta_{GWP}}{\alpha_{CLtoPA6}}$
$\alpha_{PA6toCL}$	Mass yield of CL produced per unit of PA6 input through chemical recycling	kg CL / kg	see results	see results	see results	see simulated chemical recycling processes
$GWP_{CL,annual}(t)$	Annual GWP emissions of CL produced via the respective chemical recycling pathway needed to meet estimated PA6 production volumes	Mt CO_2 Eq / year	see results	see results	see results	$GWP_{CL} * \alpha_{PA6toCL} * M_{PA6}(t)$
$ASR_{CLforPA6}$	Absolute sustainability ratio	-	see results	see results	see results	$\frac{GWP_{CL,annual}(2050)}{CB_{CLforPA6}(2050)}$

Table E 4: Mass yield of CL produced per unit of PA6 input for the four different chemical recycling pathways.

	H_3PO_4	HTW	iPrOH	NaOH
$\alpha_{PA6toCL}$	0.78	0.84	0.84	0.90

E.4 Process Level

Next to the assumptions mentioned in the methods section of the paper, the following assumptions or heuristics have been used for all processes:

- Mass or heat transfer limitations have not been considered, since the CSTRs operate at relatively high CL yield, such that high viscosities as in polymerization processes are not expected. For a relative comparison, this assumption is valid. However, small inaccuracies could occur because of deviations between partial vapor pressures and saturation pressures due to mass transfer barriers.
- The reaction scheme was strongly simplified with the assumption of first order degradation kinetics in PA6 for all processes, because kinetic models have not been established yet for PA6 depolymerisation. However, as literature shows, first order kinetics seem to fit well in a first estimate and is accurate enough for a relative comparison [59–63].
- Valves were neglected as equipment.

E.4.1 Near-Optimal Temperature Approaches, Pressure Drops and Temperature Changes

- Near-optimal minimum temperature approaches in a heat exchanger:
  - 5°C for below ambient
  - 10°C for ambient to 150°C
  - 20°C for above 150°C
- Heat exchanger pressure drops:
  - 35 kPa for liquid streams without phase change
  - 20 kPa for vapour streams without phase change
  - Neglected for condensing and boiling streams
- Pipeline and valve pressure drops were neglected.
- Temperature change across pump for liquids was neglected.

E.4.2 Heat Transfer Coefficients

- The heat transfer coefficients in W/m2/°C were chosen according to heuristics as follows [23]:
  - water – liquid: 850
  - liquid – liquid: 280
  - gas – gas: 30
  - reboiler: 1140
  - water – water: 1140
  - steam condensing: 3000
  - liquid – condensing organic vapour: 850

F Abbreviations & Nomenclature

ACA	Aminocaproic acid
ADP	abiotic depletion potential
alpha	mass based conversion factor
APOS	allocation at the point of substitution
ASR	absolute sustainability ratio
beta	allocation factor
CB(t)	carbon budget per year
CL	caprolactam
CW	cooling water
EF	Environmental Footprint
EP	freshwater eutrophication potential
EPD	Environmental Product Declarations
FET	freshwater ecotoxicity
GWP	global warming potential
H_3PO_4	phosphoric acid
HDPE	high-density polyethylene
HP	high-pressure steam
HTC	carcinogenic human toxicity
HTNC	non-carcinogenic human toxicity
HTW	high-temperature water
IPCC	Intergovernmental Panel on Climate Change
iPrOH	isopropanol
IR	ionizing radiation on human health
ISO	International Organization for Standardization
LCA	life cycle assessment
LCIA	Life Cycle Impact Assessment
LDPE	low-density polyethylene
LP	low-pressure steam
LU	land use
M	predicted production volume
MP	medium-pressure steam
NaOH	sodium hydroxide
NRTL	Non-Random Two-Liquid
OIL	No. 2 fuel oil
PA6	polyamide 6
PE	polyethylene

PET	polyethylene terephthalate
PP	polypropylene
PS	polystyrene
TEA	techno-economic assessment
VLE	vapor-liquid equilibrium
WDP	water deprivation potential

References

(1) Rwei, S.-P.; Ranganathan, P.; Lee, Y.-H. Isothermal Crystallization Kinetics Study of Fully Aliphatic PA6 Copolyamides: Effect of Novel Long-Chain Polyamide Salt as a Comonomer. *Polymers* **2019**, *11* (3). DOI: 10.3390/polym11030472. Published Online: Mar. 12, 2019.

(2) Brandrup, J.; Immergut, E. H.; Grulke, E. A.; Abe, A.; Bloch, D. R. *Polymer handbook*; Wiley New York, 1999.

(3) Gaur, U.; Lau, S.; Wunderlich, B. B.; Wunderlich, B. Heat Capacity and Other Thermodynamic Properties of Linear Macromolecules. VIII. Polyesters and Polyamides. *Journal of Physical and Chemical Reference Data* **1983**, *12* (1), 65–89. DOI: 10.1063/1.555678.

(4) ecoinvent Association. *ecoinvent database version 3.9.1*, 2022.

(5) Schneider, F.; Parsons, S.; Clift, S.; Stolte, A.; Krüger, M.; McManus, M. Life cycle assessment (LCA) on waste management options for derelict fishing gear. *Int. J. Life Cycle Assess.* **2023**, *28* (3), 274–290. DOI: 10.1007/s11367-022-02132-y.

(6) Haupt, M.; Kägi, T.; Hellweg, S. Life cycle inventories of waste management processes. *Data in brief* **2018**, *19*, 1441–1457. DOI: 10.1016/j.dib.2018.05.067. Published Online: May. 19, 2018.

(7) Costamagna, M.; Massaccesi, B. M.; Mazzucco, D.; Baricco, M.; Rizzi, P. Environmental assessment of the recycling process for polyamides - Polyethylene multilayer packaging films. *Sustain. Mater. Technol.* **2023**, *35*, e00562. DOI: 10.1016/j.susmat.2022.e00562.

(8) Hu, H.; Xu, Q.; Sun, L.; Zhu, R.; Gao, T.; He, Y.; Ma, B.; Yu, J.; Wang, X. 1 Rapid Hydrolysis of Waste and Scrap PA6 Textiles to ε-Caprolactam. *ACS Applied Polymer Materials* **2022**, *5* (1), 751–763. DOI: 10.1021/acsapm.2c01744.

(9) Wang, W.; Meng, L.; Huang, Y. Hydrolytic degradation of monomer casting nylon in subcritical water. *Polym. Degrad. Stab.* **2014**, *110*, 312–317. DOI: 10.1016/j.polymdegradstab.2014.09.014.

(10) Iwaya, T.; Sasaki, M.; Goto, M. Kinetic analysis for hydrothermal depolymerization of nylon 6. *Polymer Degradation and Stability* **2006**, *91* (9), 1989–1995. DOI: 10.1016/j.polymdegradstab.2006.02.009.

(11) Darzi, R.; Dubowski, Y.; Posmanik, R. Hydrothermal processing of polyethylene-terephthalate and nylon-6 mixture as a plastic waste upcycling treatment: A comprehensive multi-phase analysis. *Waste management (New York, N.Y.)* **2022**, *143*, 223–231. DOI: 10.1016/j.wasman.2022.03.002. Published Online: Mar. 9, 2022.

(12) Hu, H.; Xu, Q.; Sun, L.; Zhu, R.; Gao, T.; He, Y.; Ma, B.; Yu, J.; Wang, X. Rapid Hydrolysis of Waste and Scrap PA6 Textiles to ε-Caprolactam. *ACS Appl. Polym. Mater.* **2023**, *5* (1), 751–763. DOI: 10.1021/acsapm.2c01744.

(13) Wernet, G.; Bauer, C.; Steubing, B.; Reinhard, J.; Moreno-Ruiz, E.; Weidema, B. The ecoinvent database version 3 (part I): overview and methodology. *Int. J. Life Cycle Assess.* **2016**, *21* (9), 1218–1230. DOI: 10.1007/s11367-016-1087-8.

(14) Doka, G. Updates to Life Cycle Inventories of Municipal Waste Incineration. *Zürich, Switzerland* **2013**.

(15) Tonsi, G.; Maesani, C.; Alini, S.; Ortenzi, M. A.; Pirola, C. Nylon Recycling Processes: a Brief Overview. *Chemical Engineering Transactions* **2023**, *100*, 727–732.

(16) T. J. Jenczewsld; L. Crescentini; R. E. Mayer. Monomer Recovery From Multi-Component Materials (US005656757A).

(17) Beyer, C.; Richter, F. Selective depolymerisation of polyamide 6 to produce caprolactam from mixtures of caprolactam-containing polymers and polyurethane-containing polymers, in particular polyurethane block copolymers (WO 2022/129022), 2021.

(18) Hu, H.; Zhu, R.; Sun, L.; Gao, S.; Ma, B.; Gao, T.; He, Y.; Yu, J.; Wang, X. Synchronous decolorization and depolymerization of colored waste and scrap PA6 textiles containing diverse dyes to recover regenerated ε-caprolactam. *J. Anal. Appl. Pyrolysis* **2023**, *176*, 106240. DOI: 10.1016/j.jaap.2023.106240.

(19) Kamimura, A.; Sugimoto, T.; Kaiso, K. Method of depolymerizing polyamides and Method of manufacturing polyamide monomers (EP 1 801 101 B1).

(20) Seider, W. D.; Seader, J. D.; Lewin, D. R. *Product and Process Design Principles: Synthesis, Analysis and Design, 3rd Edition*; Wiley New York, 2008.

(21) Castellanos-Beltran, I. J.; Medeiros, F. G. M. de; Bensebaa, F.; Vasconcelos, B. R. de. Novel bottom-up methodology to build the lifecycle inventory of unit operations: the impact of macroscopic components. *Int. J. Life Cycle Assess.* **2023**, *28* (6), 669–683. DOI: 10.1007/s11367-023-02165-x.

(22) Minor, A.-J. *Python Aspen Plus Connected Model for the Calculation of Equipment Costs*. https://github.com/A-JMinor/Python-Aspen-Plus-Connected-Model-for-the-Calculation-of-Equipment-Costs (accessed 2023-04-18).
(23) Turton, R.; Bailie, R. C.; Whiting, W. B.; Shaeiwitz, J. A. *Analysis, synthesis and design of chemical processes*; Pearson Education, 2008.
(24) Baaqel, H.; Hallett, J. P.; Guillén-Gosálbez, G.; Chachuat, B. Sustainability Assessment of Alternative Synthesis Routes to Aprotic Ionic Liquids: The Case of 1-Butyl-3-methylimidazolium Tetrafluoroborate for Fuel Desulfurization. *ACS Sustainable Chemistry & Engineering* **2022**, *10* (1), 323–331. DOI: 10.1021/acssuschemeng.1c06188.
(25) Hischier, R.; Hellweg, S.; Capello, C.; Primas, A. Establishing Life Cycle Inventories of Chemicals Based on Differing Data Availability (9 pp). *The International Journal of Life Cycle Assessment* **2005**, *10* (1), 59–67. DOI: 10.1065/lca2004.10.181.7.
(26) Tosun, A.; Konak, G. Development of a model estimating energy consumption values of primary and secondary crushers. *Arabian Journal of Geosciences* **2015**, *8* (2), 1133–1144. DOI: 10.1007/s12517-013-1260-3.
(27) *Extruder Electricity*. https://www.ptonline.com/articles/how-much-horsepower-do-you-need (accessed 2023-03-18).
(28) *Extruder Electricity*. https://calculator.academy/extruder-power-calculator/.
(29) Engineering Toolbox. *Utility characterisation*. www.engineeringtoolbox.com (accessed 2020-11-10).
(30) Pérez-Uresti, S. I.; Martín, M.; Jiménez-Gutiérrez, A. Estimation of renewable-based steam costs. *Applied Energy* **2019**, *250*, 1120–1131. DOI: 10.1016/j.apenergy.2019.04.189.
(31) Chung, Y.-H.; Peng, T.-H.; Lee, H.-Y.; Chen, C.-L.; Chien, I.-L. Design and Control of Reactive Distillation System for Esterification of Levulinic Acid and n -Butanol. *Industrial & Engineering Chemistry Research* **2015**, *54* (13), 3341–3354. DOI: 10.1021/ie500660h.
(32) Dimian, A. C.; Bezedea, N. I.; Bildea, C. S. Novel Two-Stage Process for Manufacturing Butadiene from Ethanol. *Industrial & Engineering Chemistry Research* **2021**, *60* (23), 8475–8492. DOI: 10.1021/acs.iecr.1c00958.
(33) Guinée, J. B.; Koning, A. de; Heijungs, R. Life cycle assessment-based Absolute Environmental Sustainability Assessment is also relative. *J of Industrial Ecology* **2022**, *26* (3), 673–682. DOI: 10.1111/jiec.13260.
(34) Reuter, M.; Hudson, C.; Schaik, A.; Heiskanen, K.; Meskers, C.; Hagelüken, C.; Goessling-Reisemann, S. *Metal Recycling - Opportunities, Limits, Infrastructure*, 2013.
(35) Schrijvers, D. L.; Loubet, P.; Sonnemann, G. "Allocation at the point of substitution" applied to recycled rare earth elements: what can we learn? *Int. J. Life Cycle Assess.* **2021**, *26* (7), 1403–1416. DOI: 10.1007/s11367-021-01884-3.
(36) Bendix, P.; Achenbach, H.; Weißhaupt, P.; Eckert, D.; Oehme, I.; Berg, H. Circular economy for durable products and materials: the recycling of plastic building products in Germany—status quo, potentials and recommendations. *Journal of Material Cycles and Waste Management* **2022**, *24* (4), 1432–1443. DOI: 10.1007/s10163-022-01406-9.
(37) Ekvall, T.; Albertsson, G. S.; Jelse, K. *Modeling recycling in life cycle assessment*. https://www.diva-portal.org/smash/record.jsf?pid=diva2:1549446 (accessed 2024-12-01).
(38) Schrijvers, D. L.; Loubet, P.; Sonnemann, G. Developing a systematic framework for consistent allocation in LCA. *Int. J. Life Cycle Assess.* **2016**, *21* (7), 976–993. DOI: 10.1007/s11367-016-1063-3.
(39) Nordelöf, A.; Poulikidou, S.; Chordia, M.; Bitencourt de Oliveira, F.; Tivander, J.; Arvidsson, R. Methodological Approaches to End-Of-Life Modelling in Life Cycle Assessments of Lithium-Ion Batteries. *Batteries* **2019**, *5* (3), 51. DOI: 10.3390/batteries5030051.
(40) Davidson, M. G.; Furlong, R. A.; McManus, M. C. Developments in the life cycle assessment of chemical recycling of plastic waste – A review. *J. Cleaner Prod.* **2021**, *293*, 126163. DOI: 10.1016/j.jclepro.2021.126163.
(41) Schwarz, A.; Ferjan, Š.; Kunst, J. Life cycle assessment of advanced grade PLA product with novel end-of-life treatment through depolymerization. *Sci. Total Environ* **2023**, *905*, 167020. DOI: 10.1016/j.scitotenv.2023.167020. Published Online: Sep. 13, 2023.
(42) Laurent, A.; Clavreul, J.; Bernstad, A.; Bakas, I.; Niero, M.; Gentil, E.; Christensen, T. H.; Hauschild, M. Z. Review of LCA studies of solid waste management systems--part II: methodological guidance for a better practice. *Waste Manag.* **2014**, *34* (3), 589–606. DOI: 10.1016/j.wasman.2013.12.004. Published Online: Dec. 31, 2013.
(43) Jungbluth, N. Description of life cycle impact assessment methods: Supplementary information for tenders. *ESU-Services: Schaffhausen, Switzerland* **2024**.
(44) Lee, H.; Calvin, K.; Dasgupta, D.; Krinner, G.; Mukherji, A.; Thorne, P. W.; Trisos, C.; Romero, J.; Aldunce, P.; Barrett, K.; Blanco, G.; Cheung, W. W.; Connors, S.; Denton, F.; Diongue-Niang, A.; Dodman, D.; Garschagen,

M.; Geden, O.; Hayward, B.; Jones, C.; Jotzo, F.; Krug, T.; Lasco, R.; Lee, Y.-Y.; Masson-Delmotte, V.; Meinshausen, M.; Mintenbeck, K.; Mokssit, A.; Otto, F. E.; Pathak, M.; Pirani, A.; Poloczanska, E.; Pörtner, H.-O.; Revi, A.; Roberts, D. C.; Roy, J.; Ruane, A. C.; Skea, J.; Shukla, P. R.; Slade, R.; Slangen, A.; Sokona, Y.; Sörensson, A. A.; Tignor, M.; van Vuuren, D.; Wei, Y.-M.; Winkler, H.; Zhai, P.; Zommers, Z.; Hourcade, J.-C.; Johnson, F. X.; Pachauri, S.; Simpson, N. P.; Singh, C.; Thomas, A.; Totin, E.; Arias, P.; Bustamante, M.; Elgizouli, I.; Flato, G.; Howden, M.; Méndez-Vallejo, C.; Pereira, J. J.; Pichs-Madruga, R.; Rose, S. K.; Saheb, Y.; Sánchez Rodríguez, R.; Ürge-Vorsatz, D.; Xiao, C.; Yassaa, N.; Alegría, A.; Armour, K.; Bednar-Friedl, B.; Blok, K.; Cissé, G.; Dentener, F.; Eriksen, S.; Fischer, E.; Garner, G.; Guivarch, C.; Haasnoot, M.; Hansen, G.; Hauser, M.; Hawkins, E.; Hermans, T.; Kopp, R.; Leprince-Ringuet, N.; Lewis, J.; Ley, D.; Ludden, C.; Niamir, L.; Nicholls, Z.; Some, S.; Szopa, S.; Trewin, B.; van der Wijst, K.-I.; Winter, G.; Witting, M.; Birt, A.; Ha, M.; Kim, J.; Haites, E. F.; Jung, Y.; Stavins, R.; Orendain, D. J. A.; Ignon, L.; Park, S.; Park, Y.; Reisinger, A.; Cammaramo, D.; Fischlin, A.; Fuglestvedt, J. S.; Matthews, J. R.; Péan, C. IPCC, 2023: Climate Change 2023: Synthesis Report. Contribution of Working Groups I, II and III to the Sixth Assessment Report of the Intergovernmental Panel on Climate Change [Core Writing Team, H. Lee and J. Romero (eds.)]. IPCC, Geneva, Switzerland. DOI: 10.59327/IPCC/AR6-9789291691647.

(45) Saouter, E.; Biganzoli, F.; Ceriani, L.; Versteeg, D.; Crenna, E.; Zampori, L.; Sala, S.; Pant, R. *Environmental footprint: update of life cycle impact assessment methods-ecotoxicity freshwater, human toxicity cancer, and non-cancer*; Publications Office of the European Union Luxembourg, 2020.

(46) Struijs, J.; Beusen, A.; van Jaarsveld, H.; Huijbregts, M. A.; Goedkoop, M.; Heijungs, R.; Schryver, A. de; van Zelm, R. ReCiPe 2008 A life cycle impact assessment method which comprises harmonised category indicators at the midpoint and the endpoint level. *Report I: Characterisation factors* **2009**, 59–67.

(47) Frischknecht, R.; Braunschweig, A.; Hofstetter, P.; Suter, P. Human health damages due to ionising radiation in life cycle impact assessment. *Environmental Impact Assessment Review* **2000**, *20* (2), 159–189. DOI: 10.1016/S0195-9255(99)00042-6.

(48) Laurentiis, V. de; Secchi, M.; Bos, U.; Horn, R.; Laurent, A.; Sala, S. Soil quality index: Exploring options for a comprehensive assessment of land use impacts in LCA. *Journal of Cleaner Production* **2019**, *215*, 63–74. DOI: 10.1016/j.jclepro.2018.12.238.

(49) van Oers, L.; Guinée, J. The Abiotic Depletion Potential: Background, Updates, and Future. *Resources* **2016**, *5* (1), 16. DOI: 10.3390/resources5010016.

(50) Boulay, A.-M.; Bare, J.; Benini, L.; Berger, M.; Lathuillière, M. J.; Manzardo, A.; Margni, M.; Motoshita, M.; Núñez, M.; Pastor, A. V. The WULCA consensus characterization model for water scarcity footprints: assessing impacts of water consumption based on available water remaining (AWARE). *Int J Life Cycle Assess* **2018**, *23* (2), 368–378.

(51) Zampori, L.; Pant, R. Suggestions for updating the Product Environmental Footprint (PEF) method. *Publications Office of the European Union: Luxembourg* **2019**, *201910*, 424613.

(52) Gibon, T.; Hahn Menacho, Á. Parametric Life Cycle Assessment of Nuclear Power for Simplified Models. *Environ. Sci. Technol.* **2023**, *57* (38), 14194–14205. DOI: 10.1021/acs.est.3c03190. Published Online: Sep. 12, 2023.

(53) van den Oever, A. E.; Puricelli, S.; Costa, D.; Thonemann, N.; Lavigne Philippot, M.; Messagie, M. Revisiting the challenges of ozone depletion in life cycle assessment. *Cleaner Environmental Systems* **2024**, *13*, 100196. DOI: 10.1016/j.cesys.2024.100196.

(54) Hjalsted, A. W.; Laurent, A.; Andersen, M. M.; Olsen, K. H.; Ryberg, M.; Hauschild, M. Sharing the safe operating space: Exploring ethical allocation principles to operationalize the planetary boundaries and assess absolute sustainability at individual and industrial sector levels. *J. Ind. Ecol.* **2021**, *25* (1), 6–19. DOI: 10.1111/jiec.13050.

(55) Heide, M.; Hauschild, M. Z.; Ryberg, M. Reflecting the importance of human needs fulfilment in absolute sustainability assessments: Development of a sharing principle. *J. Ind. Ecol.* **2023**, *27* (4), 1151–1164. DOI: 10.1111/jiec.13405.

(56) Bachmann, M.; Zibunas, C.; Hartmann, J.; Tulus, V.; Suh, S.; Guillén-Gosálbez, G.; Bardow, A. Towards circular plastics within planetary boundaries. *Nat. Sustain.* **2023**, *6* (5), 599–610. DOI: 10.1038/s41893-022-01054-9.

(57) Herps, H. H. T. Modelling and Comparative Assessment of Polyamide-6 Manufacturing towards a Sustainable Chemical Industry **2020.** DOI: 10.33540/61.

(58) Hong, J.; Xu, X. Environmental impact assessment of caprolactam production – a case study in China. *J. Cleaner Prod.* **2012**, *27*, 103–108. DOI: 10.1016/j.jclepro.2011.12.037.

(59) Khuntia, S. P.; Gadgeel, A.; Mestry, S.; Mhaske, S. T. Organo-sulfonic acid catalyzed degradation kinetics and thermodynamic studies of nylon-6 by hydrothermal method. *Polymers for Advanced Technologies* **2022**, *33* (1), 411–426. DOI: 10.1002/pat.5526.
(60) Wang, W.; Meng, L.; Leng, K.; Huang, Y. Hydrolysis of waste monomer casting nylon catalyzed by solid acids. *Polymer Degradation and Stability* **2017**, *136*, 112–120. DOI: 10.1016/j.polymdegradstab.2016.12.017.
(61) Wang, W.; Meng, L.; Yu, J.; Xie, F.; Huang, Y. Enhanced hydrothermal conversion of caprolactam from waste monomer casting polyamide over H-Beta zeolite and its mechanism. *Journal of Analytical and Applied Pyrolysis* **2017**, *125*, 218–226. DOI: 10.1016/j.jaap.2017.03.020.
(62) Chen, J.; Li, Z.; Jin, L.; Ni, P.; Liu, G.; He, H.; Zhang, J.; Dong, J.; Ruan, R. Catalytic hydrothermal depolymerization of nylon 6. *J Mater Cycles Waste Manag* **2010**, *12* (4), 321–325. DOI: 10.1007/s10163-010-0304-y.
(63) Chaabani, C.; Weiss-Hortala, E.; Soudais, Y. Impact of Solvolysis Process on Both Depolymerization Kinetics of Nylon 6 and Recycling Carbon Fibers from Waste Composite. *Waste and Biomass Valorization* **2017**, *8* (8), 2853–2865. DOI: 10.1007/s12649-017-9901-5.
